# *De novo* genome assemblies of butterflies

**DOI:** 10.1093/gigascience/giab041

**Published:** 2021-06-02

**Authors:** Emily A Ellis, Caroline G Storer, Akito Y Kawahara

**Affiliations:** McGuire Center for Lepidoptera and Biodiversity, Florida Museum of Natural History, University of Florida, 3215 Hull Road, Gainesville, FL 32611–2710, USA; McGuire Center for Lepidoptera and Biodiversity, Florida Museum of Natural History, University of Florida, 3215 Hull Road, Gainesville, FL 32611–2710, USA; McGuire Center for Lepidoptera and Biodiversity, Florida Museum of Natural History, University of Florida, 3215 Hull Road, Gainesville, FL 32611–2710, USA

**Keywords:** accessibility, genomics, life sciences, open data, Papilionoidea

## Abstract

**Background:**

The availability of thousands of genomes has enabled new advancements in biology. However, many genomes have not been investigated for their quality. Here we examine quality trends in a taxonomically diverse and well-known group, butterflies (Papilionoidea), and provide draft, *de novo* assemblies for all available butterfly genomes. Owing to massive genome sequencing investment and taxonomic curation, this is an excellent group to explore genome quality.

**Findings:**

We provide *de novo* assemblies for all 822 available butterfly genomes and interpret their quality in terms of completeness and continuity. We identify the 50 highest quality genomes across butterflies and conclude that the ringlet, *Aphantopus hyperantus*, has the highest quality genome. Our post-processing of draft genome assemblies identified 118 butterfly genomes that should not be reused owing to contamination or extremely low quality. However, many draft genomes are of high utility, especially because permissibility of low-quality genomes is dependent on the objective of the study. Our assemblies will serve as a key resource for papilionid genomics, especially for researchers without computational resources.

**Conclusions:**

Quality metrics and assemblies are typically presented with annotated genome accessions but rarely with *de novo* genomes. We recommend that studies presenting genome sequences provide the assembly and some metrics of quality because quality will significantly affect downstream results. Transparency in quality metrics is needed to improve the field of genome science and encourage data reuse.

## Introduction

The explosion of available genomes across the Tree of Life has created entirely new fields of science and is changing how we investigate long-standing questions in biology. Studies of gene family evolution and gene mutation have expanded from single genes to mapping the architecture of entire genomes. Macroevolutionary studies using genomic data are now regularly being generated at impressive scales, e.g., complete class [1], continent [2], and spanning up to 500 million years [3]. As the scope of questions addressed with genomic data continues to expand, determining the effect of read length and genome completeness on results is vital. One metric that is often applied to assembled genomes is the N50 score, a weighted median statistic of contig continuity that describes the distribution of contig lengths. The N50 value indicates that half of the assembly is contained in contigs or scaffolds equal to or larger than the value. Assemblies with low N50s are more fragmented and have contigs or scaffolds with less overlap with one another. Completeness of a draft assembly can also be assessed using BUSCO scores [4]. This measure uses a taxonomically informed set of “core” protein-coding orthologs that are theoretically present in a given taxon to evaluate genomic completeness. BUSCO may detect both haplotypes sequenced from diploid tissue with adequate genome coverage. However, high heterozygosity can lead to more fragmented assemblies (low N50), potentially reducing the number of complete protein-coding genes recovered. These scores can be influenced by biological variation through natural variation in chromosome length or lineage-wide loss of core orthologs, but also by systematic error, as in poor sequencing depth [4]. Genomes may be of low quality in terms of continuity, completeness, or a combination of these 2 metrics. Understanding how genomes with low-quality metrics affect downstream analyses is critical.

Here, we provide draft *de novo* genome assemblies and quality metrics for butterflies that will be useful for studying Lepidoptera evolution, gene discovery, and genomics. To understand how genome quality varies across taxa, we examine genome assembly quality in this exemplar group of organisms that has >935 published genomes. Additionally, we explore potential uses of these data, bearing in mind their draft nature, and discuss the state of butterfly genomics in light of genome quality.

Gene family evolution and mutation holds immense potential in uncovering the mechanisms behind rapid functional adaptation and potential subsequent speciation [5, 6], and significant progress is being made in this area with the inclusion of genomic data [3]. *De novo* genome assemblies allow for the discovery of novel genes with important ecological implications. For example, genes and gene duplications associated with plant detoxification can be identified [7]. Additionally, expansions of a particular gene copy can be indicative of functional adaptation (e.g., [8, 9]). However, inaccurate assessment of gene copy number will lead to false interpretations. Denton et al. [10] document a pattern of gene misassembly and false gene duplication rates in draft genomes, with gene number either overestimated or underestimated in 40% of all gene families. The mechanism of such error is closely tied to N50, such that when genes are fragmented (low N50), multiple contigs are assembled into non-biological contigs [10]. These types of errors will present as misidentification of gene duplication and loss, as well as non-biological mutations. Gene family evolution and mutation holds immense potential in uncovering the mechanisms behind rapid functional adaptation and potential subsequent speciation [5, 6], and significant progress is being made in this area with the inclusion of genomic data [3]. Including sequences of known identity to identify regions of sequencing artefacts or incorrect annotation and implementing assembly error estimation [11] may mitigate these challenges.

Phylogenetic studies stand to gain enormous taxonomic ground into the 2020s, primarily owing to the explosion of low-coverage genomes that are particularly well suited for phylogenetic studies. Taxonomic coverage in phylogenetic studies is increasing exponentially with the ability to sequence genomes from historical or museum specimens. Advances in both cost and quality of sequencing, as well as the ability to sequence DNA from degraded museum samples [12–15], allow researchers to now produce phylogenies including all extant, and even extinct species in a taxonomic group [16]. Stringency standards for including genomes in phylogenetic studies are not well established, and poor-quality genomes can produce erroneous assemblies of genes of interest [10], as detailed above. Furthermore, quality scores that highlight the completeness of a genome may serve an important quality control step for the inclusion of genomes in phylogenies, and we recommend that researchers prioritize this quality metric for phylogenetic inference. A more complete genome suggests that the sample possesses common and complete protein-coding genes, and thus it is more likely to include the researcher's set of orthologs. By assessing genome completeness, future systematic error due to taxa with low matrix occupancy may be avoided [17].

Here, we provide 822 draft de novo genome assemblies and quality metrics for a taxonomically diverse, well known group, butterflies, that will be useful for studies on their evolution, gene discovery, and genomics. We explore potential uses of these data, bearing in mind their draft nature, and discuss the state of butterfly genomics in light of genome quality.

## Methods

We obtained all published genome assemblies and genomic reads of butterflies (Lepidoptera: Papilionoidea) from NCBI [18] and LepBase [19] databases as of 1 July 2020. In the case of NCBI genome assemblies, we searched using the taxonomy database (keywords “Papilionoidea” and “papilionoid”) for the latest assemblies. When multiple were available, we selected the most recently submitted assembly (as of 1 July 2020; see Supplementary Table S1). We also searched the SRA database [18] and published literature for available paired-end, whole-body, whole shotgun genome sequences of papilionoid species [13, 20–31] (search terms butterfly genome; papilionoid genome; butterfly shotgun genome; searches concluded on 1 July 2020).

We trimmed reads using TrimGalore [32] requiring a quality score of 20 and read length of 30. We assembled reads using SPAdes (SPAdes, RRID:SCR_000131) v3.13 [33] using paired reads and allowing values of K to vary based on read length. For the majority of the *de novo* genomes, 32 CPUs and 128 GB of memory were sufficient. Forty genomes required additional memory; we ran these genomes with 24 threads with 720 GB of memory, potentially due to deeper sequencing or greater genomic complexity.

Following assembly, we performed several post-processing steps to ensure sequence integrity. First, we identified and removed contigs composed of <200 bp using SeqTK (Seqtk, RRID:SCR_018927) [34]. We scanned for evidence of vector contamination using VecScreen (VecScreen, RRID:SCR_016577) [35] and removed affected contigs. Then, we used the NCBI contaminant screening database to identify common contaminants, such as from fungi or bacteria, and removed those contaminant sequences.

To assess assembly quality, we first used assembly-stats [36] to quantify scaffold N50 for each cleaned, contaminant-free assembly. This measure estimates the contiguity of assembly contigs and describes the contig length of half of the genome; i.e., 50% of the genome includes contigs greater than or equal to this length. We also used BUSCO (BUSCO, RRID:SCR_015008) v3.02 [4] to determine the presence of a set of 1,658 core insect single-copy genes (version 9) that are highly conserved across insects and give an approximation of the completeness of the assembly. Herein, we evaluate only the BUSCO Complete score, which requires each of the 1,658 core ortholog genes in the assembly to include both start and stop codons. For the full BUSCO score report, see Supplemental Tables S1–S3. A custom script, filter_seqs_by_NCBI.py (Supplementary File 1), was created to automate NCBI required edits. This script uses the text feedback file from NCBI and will be useful for researchers willing to make their assemblies available on NCBI.

## Results

We assembled 873 papilionoid genomes using raw reads from the NCBI SRA database and downloaded 62 pre-assembled genomes from the NCBI Assembly database [18]. These 935 butterfly samples with genomic data represent 665 unique species because some species have multiple subspecies sequenced or have replicate genomes (Supplementary Table S1). We did not attempt to combine genomic reads from multiple conspecific individuals because this will artificially increase heterozygosity and inevitably affect assembly quality [37]. All genomes assembled for this study (Supplementary Table S1) are available for download through the TPA Database (BioProject PRJNA606954) and quality statistics calculated for each genome are listed in Supplementary Table S1.

**Figure 1:**
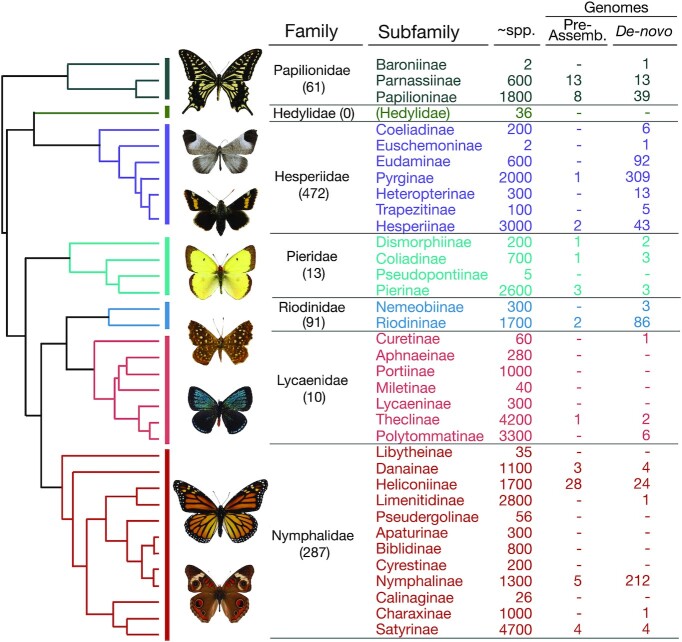
Pre-assembled and *de novo* assembled genomes for each butterfly and subfamily shown on a topological sketch of [38]. Species-richness estimates and topology are presented for comparison only.

Pre-assembled genomes from NCBI and LepBase span 6 butterfly families and 12 subfamilies; our *de novo* assembled genomes represent 6 families and 24 subfamilies (Fig. 1). The only family for which no public genomic data are available is the Hedylidae, a family with only 36 described neotropical species [39]. Hesperiidae has the greatest number of species with available genomic data (472), more than half of which are in subfamily Pyrginae (310), largely due to research by Grishin and colleagues [13, 20–27, 30, 31] (Fig. 1). The Nymphalidae, the most species-rich family of butterflies, have 287 genomes available, and 210 of these genomes are in the genus *Junonia* (Fig. 1). The Lycaenidae have comparatively few genomes available (10), given its high species richness (Fig. 1).

**Figure 2:**
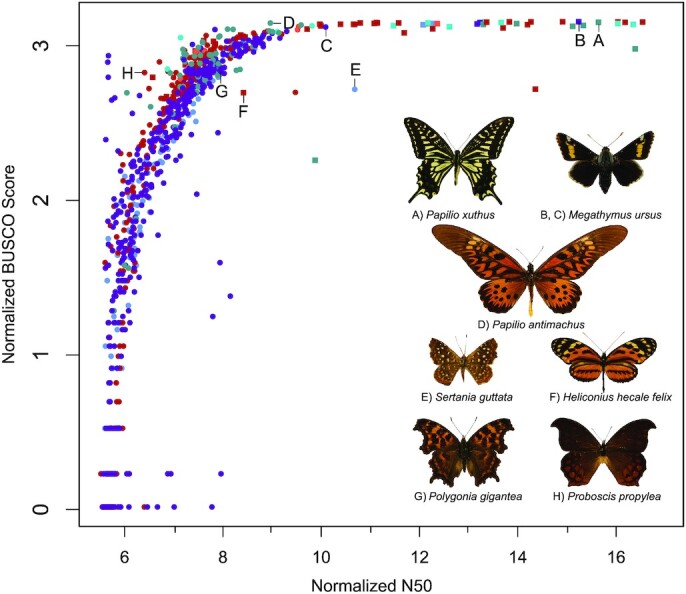
Natural log–normalized N50 and BUSCO scores plotted for both pre-assembled (squares) and *de novo* (circles) genome assemblies. Colors denote taxonomic family designation, as in Fig. 1. Letters correspond to inset images of representative species.

The metrics that we used revealed large variance in genome assembly quality. N50 and BUSCO scores were often similar, such that the highest quality genomes typically had both high N50 and high BUSCO scores, although this was not always the case (Fig. 2). These quality statistics measure 2 different aspects of quality and should be used in conjunction because length distribution may not be associated with gene content [4]. Pre-assembled genomes downloaded from NCBI and LepBase on average had high quality scores (Supplementary Table S2, Fig. 2) (scaffold N50 = 1,706,589 bp; BUSCO = 81.2%). Of these, 5 *Heliconius* genomes (*H. doris,H. hecuba flava, H. hierax, H. wallacei, and H. xanthocles*) have notably lower mean quality scores (N50 = 996.6 bp; BUSCO = 33.66%). The *H. hierax* genome (GCA_900068475.1) had the lowest quality measures of the pre-assembled genomes that we investigated (N50 = 916 bp; BUSCO = 30.5). The satyrine *Aphantopus hyperantus* (GCA_902806685.1) had the highest quality scores of all genomes investigated (N50 = 15,230,192 bp; BUSCO = 97.8%).

Quality scores varied widely among the draft *de novo* genome assemblies (Fig. 2). In 51 cases, we found that assemblies comprised only short (<200 bp) fragments and contaminants. In these cases, we removed the assembly and report the N50 score as zero (Supplementary Tables S1 and S3). N50 ranged from 249 bp in *Junonia evarete nigrosuffusa* (SRR10765819; Nymphalidae) to 43,550 bp in *Sertania guttata guttata* (Fig. 2E; SRR10158585; Riodinidae). One hundred seven *de novo* genomes resulted in a BUSCO score of 0% (Supplementary Tables S1 and S3), meaning that these genomes recovered none of the core insect orthologs. Seven had BUSCO scores of ≥90%, with the greatest BUSCO score (96.4%) from *Papilio antimachus* (Fig. 2D; SRR8954523 [29]). The mean quality scores of the *de novo* genomes were low (N50 = 15,650 bp; BUSCO = 28.25%; excluding zero values). *Proboscis propylea* (Fig. 2H) had a greater than average BUSCO score, but low N50 (N50 = 605 bp; BUSCO = 45.3%). In an effort to identify the best exemplar genome for each major butterfly lineage, we present the highest quality genomes per subfamily (Table 1). Table 2 summarizes the 50 highest quality butterfly *denovo* and preassembled genomes, regardless of taxonomy.

**Table 1:** Highest quality genomes by butterfly subfamily, according to N50 and BUSCO scores

Taxonomy	Organism	Accession ID	N50 (bp)	BUSCO 3 (C%)
Hesperiidae; Coeliadinae	*Choaspes benjaminii*	SRR7174556	2,532	87.3
Hesperiidae; Eudaminae	*Phocides pigmalion*	SRR7174453	9,497	76.7
Hesperiidae; Hesperiinae	*Megathymus ursus*	GCA_003671415.1	4,153,133	98.3
Hesperiidae; Heteropterinae	*Dalla quadristriga*	SRR9330377	4,259	69.8
Hesperiidae; Pyrginae	*Cecropterus lyciades*	GCA_002930495.1	558,064	97.3
Hesperiidae; Trapezitinae	*Toxidia parvulus*	SRR9330370	932	21.7
Lycaenidae; Curetinae	*Curetis bulis*	SRR10158559	1,108	28.3
Lycaenidae; Polyommatinae	*Cyclargus thomasi*	SRR6727422	13,909	91.3
Lycaenidae; Theclinae	*Calycopis cecrops*	GCA_001625245.1	233,537	95.5
Nymphalidae; Charaxinae	*Charaxes varanes*	SRR5175869	1,531	49.5
Nymphalidae; Danainae	*Danaus plexippus*	GCA_009731565.1	9,209,872	93.9
Nymphalidae; Heliconiinae	*Heliconius erato*	LepBase_Heliconius_erato_demophoon_v1	10,688,973	97.4
Nymphalidae; Limentidinae	*Limenitis arthemis*	SRR1504973	631	12.6
Nymphalidae; Morphinae	*Taenaris catops*	GCA_009936525.1	1,720,500	35.2
Nymphalidae; Nymphalinae	*Vanessa tameamea*	GCA_002938995.1	2,988,984	98.3
Nymphalidae; Satyrinae	*Aphantopus hyperantus*	GCA_902806685.1	15,230,192	97.8
Papilionidae; Baroniinae	*Baronia brevicornis*	SRR8954515	1,886	59.0
Papilionidae; Papilioninae	*Papilio xuthus*	GCA_000836235.1	6,198,915	97.6
Papilionidae; Parnassiinae	*Sericinus montela*	SRR8954536	3,584	59.4
Pieridae; Coliadinae	*Zerene cesonia*	GCA_012273895.1	9,214,832	95.6
Pieridae; Dismorphiinae	*Leptidea sinapis*	GCA_900199415.2	857,189	97.2
Pieridae; Pierinae	*Pieris napi*	LepBase_Pieris_napi_v1.1	12,597,868	94.4
Riodinidae; Nemeobiinae	*Euselasia chrysippe*	SRR10158562	1,806	30.3
Riodinidae; Riodininae	*Calephelis nemesis*	GCA_002245505.1	206,312	95.6

C: complete.

**Table 2:** Highest 50 quality papilionoid genome assemblies, regardless of subfamily, ranked using natural log–normalized N50 and BUSCO Complete scores

Rank	Organism	Accession ID	N50 (bp)	BUSCO 3 (C%)
1	*Aphantopus hyperantus*	GCA_902806685.1	15,230,192	97.8
2	*Pieris napi*	LepBase_Pieris_napi_v1.1	12,597,868	94.4
3	*Heliconius erato*	LepBase_Heliconius_erato_demophoon_v1	10,688,973	97.4
4	*Danaus plexippus*	GCA_009731565.1	9,209,872	98.0
5	*Zerene cesonia*	GCA_012273895.1	9,214,832	95.6
6	*Papilio xuthus*	GCA_000836235.1	6,198,915	97.6
7	*Papilio bianor*	GCA_011763625.1	13,111,833	65.0
8	*Megathymus ursus*	GCA_003671415.1	4,153,133	98.3
9	*Papilio memnon*	GCA_003118415.2	4,560,862	92.9
10	*Papilio polytes*	GCA_000836215.1	3,672,263	91.8
11	*Vanessa tameamea*	GCA_002938995.1	2,988,984	98.3
12	*Junonia coenia*	LepBase_Junonia_coenia_JC_v1.0	1,571,165	98.2
13	*Danaus chryssipus*	GCA_004959915.1	1,465,393	93.9
14	*Papilio machaon*	GCA_001298355.1	1,174,287	95.5
15	*Hypolimnas misippus*	GCA_008963455.1	1,011,763	98.1
16	*Leptidea sinapsis*	GCA_900199415.2	857,189	97.2
17	*Danaus melanippus*	GCA_010014825.1	889,656	89.4
18	*Bicyclus anynana*	GCA_900239965.1	638,282	97.6
19	*Pieris rapae*	GCA_001856805.1	617,301	98.0
20	*Papilio dardanus*	GCA_013186455.1	596,599	94.3
21	*Cecropterus lyciades*	GCA_002930495.1	558,064	97.3
22	*Lerema accius*	GCA_001278395.1	525,349	95.1
23	*Phoebis sennae*	GCA_001586405.1	299,140	91.1
24	*Taenaris catops*	GCA_009936525.1	1,720,500	35.2
25	*Calycopis cecrops*	GCA_001625245.1	233,537	95.5
26	*Papilio glaucus*	GCA_000931545.1	230,841	95.5
27	*Calephelis nemesis*	GCA_002245505.1	206,312	95.6
28	*Delias pasithoe*	GCA_010014985.1	193,720	96.5
29	*Heliconius melpomene*	GCA_000313835.2	194,302	95.6
30	*Maniola jurtina*	GCA_009667785.1	212,945	88.3
31	*Calephelis virginiensis*	GCA_002245475.1	175,106	93.9
32	*Heliconius burneyi*	LepBase_Heliconius_burneyi_helico3	106,325	96.5
33	*Melitaea cinxia*	GCA_000716385.1	119,328	83.0
34	*Colias croceus*	GCA_009982905.1	95,765	92.5
35	*Heliconius hecalesia*	LepBase_Heliconius_hecalesia_helico3	68,855	96.5
36	*Heliconius demeter*	Lepbase_Heliconius_demeter_helico3	67,995	96.8
37	*Heliconius besckei*	LepBase_Heliconius_besckei_helico3	64,778	95.8
38	*Heliconius himera*	LepBase_Heliconius_himera_helico3	48,684	96.5
39	*Heliconius sara*	LepBase_Heliconius_sara_helico3	43,390	94.3
40	*Heliconius telesiphe*	LepBase_Heliconius_telesiphe_helico3	42,672	94.7
41	*Eueides tales*	LepBase_Eueides_tales_helico3	32,552	94.7
42	*Megathymus ursus*	SRR7174358	24,120	90.7
43	*Agraulis vanillae*	LepBase_Agraulis_vanillae_helico3	21,413	94.6
44	*Dryas iulia*	LepBase_Dryas_iulia_helico3	21,916	92.3
45	*Delias oraia*	SRR4341246	18,269	92.3
46	*Pararge aegeria*	GCA_900499025.1	16,525	88.0
47	*Atrophaneura dixoni*	SRR8954516	14,618	93.5
48	*Cyclargus thomasi*	SRR6727422	13,909	91.3
49	*Eumaeus atala*	SRR6727440	13,611	87.0
50	*Sertania guttata*	SRR10158585	43,550	35.1

## Discussion

High-quality genomes are required for studies that span the biological sciences, from gene family, mutation research to macroevolutionary phylogenetics and population dynamics. Our results show that available genomes vary widely in quality and taxonomic coverage. The significant variance in N50 and BUSCO scores highlights an important message: in the scientific literature, a “genome” can range from genomic fragments to fully annotated chromosomes. Large-scale genomic studies, especially those that sequence species in an entire clade or geographic region, represent great scientific feats, but if they are based on many low-quality genomes, they may not be useful for subsequent studies. We encourage peer-reviewed journals and public databases to require authors to report genome quality via N50 and BUSCO, which can be accessioned with the assembly on NCBI as Global Statistics. Doing so provides maximum transparency, reproducibility, and a holistic view of future data reuse. In this way, users can easily evaluate whether the quality of the genome is high enough to investigate gene family diversification (prioritize N50) or phylogenetic systematics (prioritize BUSCO).

Our analyses highlight the extensive variation in genome quality. Part of this discrepancy can be alleviated with changes in language. Perhaps we should begin referring to low-quality genomes, such as *J. evarete nigrosuffusa* (SRR10765819; N50 = 249 bp; BUSCO = 0.1%), as “genomic data,” as opposed to the potentially misleading term, “genome.” Next, accessioning all assemblies would save countless hours of computation time and allow for the validation of results. In addition, assemblies would also allow results (e.g., gene family evolution, sequence identification, ortholog determination) from previous studies to be validated. Accessioning should include low-coverage draft genome assemblies, which can also be deposited in the NCBI's Assembly database. These assemblies have notably lower N50 and BUSCO scores when compared to the average assembly from NCBI and LepBase [19]. Quality metrics of our *de novo* assembled genomes were, in many cases, comparable to the 5 *Heliconius* genomes that we investigated, suggesting that even low-quality genome assemblies can and should be accessioned. Including quality scores (as Global Statistics) for each draft assembly via the NCBI Assembly database (in addition to taxon-specific genome databases, such as Lepbase [19]) would provide a transparent overview of available genomes for future studies.

Genome assembly requires considerable computational resources, and assessing genome quality simply from raw file size on GenBank can be misleading. Many studies in the biological and medical sciences rely on existing genomes and their annotations (e.g., [40]). If researchers independently assemble genomes, this can lead to duplicated effort and significant time investment. Furthermore, if raw data quality is poor, assemblies likely will not be useful. In our study, we found that ≥51 of the 873 genomes that we assembled are ultimately unusable, and another 67 should be reused only with caution (Supplementary Table S1). These 118 samples produced assemblies that entirely comprised contamination or contigs <200 bp or were devoid of core insect genes, or a combination of these factors. However, it is possible that alternate assembly methods could produce a better assembly. Low N50 and low BUSCO assemblies are likely composed of fragmented genes, and, most likely, the contigs that are present are the result of very low sequence coverage. This low coverage is indicative of a high error rate and greater likelihood of incorrect sequence frame. As such, while we provide these extremely low-quality genomes, users should exercise caution in mining genes from these samples owing to the high probability of error. Reporting N50 and BUSCO, as well as genome assemblies, in manuscripts and databases promotes transparency and discourages needless computation.

Contamination has been shown to be a pervasive pattern in genome and transcriptome sequencing projects, especially those that use multiplexed sequencing approaches [41–43]. In a recent study, Allio et al. [29] found that cross-contamination accounted for 0.26% of assembly contigs. While contaminants were removed from Allio et al. [29] using CroCo [44] and thus do not affect their results, it remains unknown how much these contaminant sequences will impact future studies that reuse these genomic data. The authors did not accession genome assemblies that had contaminants removed, and contaminants remain in accessioned reads. Furthermore, it is impossible to repeat these necessary decontamination steps without detailed information regarding multiplex strategy [44]. Accessioning decontaminated assemblies to NCBI is a necessary and easy solution.

Our study reveals a significant lack of standardization and reporting across genomic studies because many do not provide genome assemblies and necessary quality metrics. Our main conclusions are that:

1. We provide draft assemblies and quality metrics for all butterfly genomes available at the time of this study (available through NCBI TPA database) (Supplementary Table S1). We synthesize these data into tables of the 50 highest quality genomes, as well as exemplar genomes for each subfamily.
2. We found that the ringlet, *Aphantopus hyperantus*, has the highest quality papilionoid genome, and that ≥51 of 873 genomes that we assembled are ultimately unusable, and another 67 should be reused only with caution. Long and contiguous reads, indicated by high N50 values, are 1 quality metric that should be reported in all studies, especially those of gene mutation, duplication, or genomic architecture.
3. Quality metrics, such as sequence length, whether sequences are contiguous, and N50 and BUSCO scores, should be reported in all studies. Phylogenetic studies are strengthened when genomes with a high completeness score, such as BUSCO, are used.
4. Researchers should provide draft assemblies in all genome publications and databases. Accessioning quality scores will enhance transparency and avoid unnecessary use of computational resources. Accessioning assemblies further promotes the FAIR principles of interoperability and reuse by limiting contaminant sequences and allowing confirmation of results.

## Data Availability

See Supplementary Tables S1–S3 for genomic read accession numbers used in this study and associated metadata. The 822 viable genome assemblies produced using SPAdes v3.13 are available in the NCBI TPA repository and can be accessed with BioProject PRJNA606954. The sequence assemblies, BUSCO files, scripts, and other supporting data underlying this article are also available via the *GigaScience* database, GigaDB [45].

## Additional Files

**Supplementary Table S1**. Sample ID, N50, BUSCO, and sequencing metadata for *de novo* assembled genomes.

**Supplementary Table S2**. Sample ID, N50, BUSCO, and sequencing metadata for pre-assembled genomes.

**Supplementary Table S3**. Sample ID, N50, BUSCO, and sequencing metadata for *de novo* genomes resulting in extremely poor quality assemblies.

**Supplementary File S1**. Filter_seqs_by_NCBI.py script used to automatically update assemblies with the feedback file from NCBI during the NCBI Accession process.

## Abbreviations

bp: base pair; BUSCO: Benchmarking Universal Single-Copy Orthologs; CPU: central processing unit; FAIR: Findability, Accessibility, Interoperability, and Reuse; NCBI: National Center for Biotechnology Information; SPAdes: St. Petersburg genome Assembler; SRA: Sequence Read Archive; TPA: third party database.

## Competing Interests

The authors declare that they have no competing interests.

## Funding

This work was funded by the National Science Foundation Grants DEB No. 1,541,500 and No. 1,557,007 to A.Y.K.

## Authors’ Contributions

A.Y.K. conceived of the study. E.A.E. performed data collection, data analysis, and produced the figures and scripts, with overall guidance from A.Y.K. All authors wrote the manuscript. C.G.S. and E.A.E. deposited the data.

## Supplementary Material

giab041_GIGA-D-20-00047_Original_Submission

giab041_GIGA-D-20-00047_Revision_1

giab041_GIGA-D-20-00047_Revision_2

giab041_GIGA-D-20-00047_Revision_3

giab041_GIGA-D-20-00047_Revision_4

giab041_Response_to_Reviewer_Comments_Original_Submission

giab041_Response_to_Reviewer_Comments_Revision_1

giab041_Response_to_Reviewer_Comments_Revision_2

giab041_Response_to_Reviewer_Comments_Revision_3

giab041_Reviewer_1_Report_Original_Submission Chris Wheat, PhD -- 3/31/2020 Reviewed

giab041_Reviewer_1_Report_Revision_1 Chris Wheat, PhD -- 8/5/2020 Reviewed

giab041_Reviewer_2_Report_Original_Submission Sujai Kumar -- 4/20/2020 Reviewed

giab041_Supplemental_Files
