# Supplementary material for: De novo genome assemblies of butterflies
Source: Gigascience. 2021 Jun 2;10(6):giab041. doi: 10.1093/gigascience/giab041 (PMC8170690; doi:10.1093/gigascience/giab041)
Supplement: giab041_GIGA-D-20-00047_Original_Submission [file giab041_giga-d-20-00047_original_submission.pdf]

GigaScience

Genome quality variation highlights the importance of transparency for data reuse

--Manuscript Draft--

**Manuscript Number:**	GIGA-D-20-00047
**Full Title:**	Genome quality variation highlights the importance of transparency for data reuse
**Article Type:**	Data Note
**Funding Information:**	National Science Foundation (DEB 1541500) — Dr. Akito Y Kawahara<br>National Science Foundation (DEB 1557007) — Dr. Akito Y Kawahara
**Abstract:**	Background<br><br>The current genomic age has led to the availability of thousands of genomes and enabled new advancements in biology. However, as the number of genomes increases, considerable attention should be given to their quality. Here we examine these trends in a taxonomically diverse and well-known group, butterflies. Due to massive genome sequencing investment and taxonomic curation, this is an excellent group to explore genome quality.Findings<br><br>We provide de-novo assemblies for all 606 available butterfly genomes, interpret their quality, and provide general guidelines for future use. These assemblies will serve as a key resource for papilionoid genomics, especially for researchers without computational resources. Using a novel quality metric, the Composite Quality Score (CQS), we identify the 50 highest quality genomes across butterflies, with Papilio xuthus as the highest quality butterfly genome. We also use this metric to report the 53 butterfly genomes that are of extremely poor quality and caution their reuse. Permissibility of low quality genomes is dependent on the objective of the study, and we discuss the potential outcomes of including low quality genomes in various future studies. Conclusions<br><br>Quality metrics are often not presented in studies that use genomic data, but should be reported as genome quality can have a significant impact on downstream results. We recommend that studies that present new genomes provide the assembly and CQS score, at minimum. Transparency in quality metrics are needed to improve the field of genome science and encourage data reuse.
**Corresponding Author:**	Emily A Ellis, Ph.D.<br>University of Florida<br>Gainesville, FL UNITED STATES
**Corresponding Author Secondary Information:**	
**Corresponding Author's Institution:**	University of Florida
**Corresponding Author's Secondary Institution:**	
**First Author:**	Emily A Ellis, Ph.D.
**First Author Secondary Information:**	
**Order of Authors:**	Emily A Ellis, Ph.D.<br>Akito Y Kawahara, PhD
**Order of Authors Secondary Information:**	
**Additional Information:**	
**Question**	**Response**
Are you submitting this manuscript to a	No

Powered by Editorial Manager® and ProduXion Manager® from Aries Systems Corporation

special series or article collection?	
**Experimental design and statistics**<br><br>Full details of the experimental design and statistical methods used should be given in the Methods section, as detailed in our Minimum Standards Reporting Checklist. Information essential to interpreting the data presented should be made available in the figure legends.<br><br>Have you included all the information requested in your manuscript?	Yes
**Resources**<br><br>A description of all resources used, including antibodies, cell lines, animals and software tools, with enough information to allow them to be uniquely identified, should be included in the Methods section. Authors are strongly encouraged to cite Research Resource Identifiers (RRIDs) for antibodies, model organisms and tools, where possible.<br><br>Have you included the information requested as detailed in our Minimum Standards Reporting Checklist?	Yes
**Availability of data and materials**<br><br>All datasets and code on which the conclusions of the paper rely must be either included in your submission or deposited in publicly available repositories (where available and ethically appropriate), referencing such data using a unique identifier in the references and in the "Availability of Data and Materials" section of your manuscript.<br><br>Have you have met the above requirement as detailed in our Minimum Standards Reporting Checklist?	Yes

Powered by Editorial Manager® and ProduXion Manager® from Aries Systems Corporation

Powered by Editorial Manager® and ProduXion Manager® from Aries Systems Corporation

Manuscript
Click here to download Manuscript 02022020_Final_GenomePaper_wref.docx

Abstract

Background: The current genomic age has led to the availability of thousands of genomes and enabled new advancements in biology. However, as the number of genomes increases, considerable attention should be given to their quality. Here we examine these trends in a taxonomically diverse and well-known group, butterflies. Due to massive genome sequencing investment and taxonomic curation, this is an excellent group to explore genome quality.

Findings: We provide *de-novo* assemblies for all 606 available butterfly genomes, interpret their quality, and provide general guidelines for future use. These assemblies will serve as a key resource for papilionoid genomics, especially for researchers without computational resources. Using a novel quality metric, the Composite Quality Score (CQS), we identify the 50 highest quality genomes across butterflies, with *Papilio xuthus* as the highest quality butterfly genome. We also use this metric to report the 53 butterfly genomes that are of extremely poor quality and caution their reuse. Permissibility of low quality genomes is dependent on the objective of the study, and we discuss the potential outcomes of including low quality genomes in various future studies.

Conclusions: Quality metrics are often not presented in studies that use genomic data, but should be reported as genome quality can have a significant impact on downstream results. We recommend that studies that present new genomes provide the assembly and CQS score, at minimum. Transparency in quality metrics are needed to improve the field of genome science and encourage data reuse.

Keywords (3-10)

Genomics, Accessibility, Open Data, Life Sciences, Papilionoidea

Introduction

The explosion of available genomes across the Tree of Life has created entirely new fields of science and is changing how we investigate long-standing questions in biology. Studies of gene family evolution and gene mutation have expanded from single genes to mapping the architecture of entire genomes. Macroevolutionary phylogenies using genomic data are now regularly being generated at impressive scales, e.g. complete Class [1], continent [2], and spanning 500 million years [3]. As the scope of questions addressed with genomic data continues to expand, determining the impact of read length and genome completeness on results is vital. One metric that is often applied to assembled genomes is an N50 score, a weighted median statistic of contig continuity that describes the distribution of contig lengths. The N50 value indicates that half of the assembly is contained in contigs or scaffolds equal to or larger than the value. Assemblies with low N50s are more fragmented and the contigs or scaffolds have less overlap with one another. Completeness of a draft assembly can also be assessed using BUSCO scores [4]. This measure uses a taxonomically informed set of core orthologs that are theoretically present in a given taxon to evaluate genomic completeness. Greater sequencing coverage should provide more complete genomes, elevating the BUSCO score. These scores can be influenced by biological variation, as in natural variation in chromosome length, or in lineage-wide loss of core orthologs, but also by systematic error, as in poor sequencing depth [4]. Genomes may be of low quality in terms of continuity, completeness, or a combination of these two metrics. Understanding how genomes with low quality metrics impact future results is of high importance. We present three examples of how the quality of genomes can influence downstream results.

First, genome quality can impact gene family analyses. Gene family evolution plays a large role in the adaptation to new environments and is hypothesized to be a primary driver of speciation, such as in plant detoxification [5]. For example, new copies of genes duplicate and subsequently produce novel functions. Expansions of a particular gene copy are often indicative

of a functional adaptation (e.g. [6,7], therefore inaccurate assessment of gene copy number will lead to false interpretations. Denton et al. [8] explored this pattern using draft genomes and found that gene number was either over- or under-estimated in 40% of all gene families. The mechanism of such error is closely tied to N50, such that when genes are fragmented (low N50), and multiple contigs are assembled into non-biological contigs [8]. These types of errors will present as misidentification of gene duplication and loss, as well as non-biological mutations. Gene duplication may also be driven by the genomic structure surrounding genes, such as transposons. Large expanses of the genome must be contiguous in order to make accurate predictions of genomic material surrounding genes. Gene family evolution and mutation holds immense potential in uncovering the mechanisms behind rapid functional adaptation and potential subsequent speciation [9,10], and significant progress is being made in this area [3]. It is important to note, however, that low-quality draft genomes are unable to provide accurate copy number and landscape information and may drastically mislead our inferences into adaptation and speciation.

A second example of how genomes with low quality can impact downstream analyses is that they can reduce mapping efficiency. Gene network analyses are used to study complex interactions between genes, combining genomes and gene expression [11]. If genes (called nodes) are missing from the genome, then gene expression data from transcriptomes cannot be mapped to the genome. Using CRISPR, researchers can target and remove specific genes and alter the proteins produced by the organism [12]. If similar sequences exist in the genome, off-target site binding may occur [13], and editing efficiency dramatically improves when genomic architecture is well known [14]. Complete genomes enable accurate target mapping and potential off-targets to be investigated.

A third example is that genome completeness can influence phylogenetic inference. Phylogenetic studies stand to gain enormous taxonomic ground into the 2020s. Taxonomic coverage in phylogenetic studies is increasing exponentially with the ability to sequence

genomes from historical or museum specimens. Advances in both cost and quality of sequencing, as well as the ability to sequence DNA from degraded museum samples [15–18] allows researchers to now produce phylogenies including all extant, and even extinct species in a taxonomic group [19]. Stringency standards for including genomes in phylogenetic studies are not well established, and poor-quality genomes can produce erroneous assemblies of genes of interest [8]. Scores that highlight the completeness of the genome may serve an important quality control step for the inclusion of genomes in phylogenies, and we recommend researchers prioritize this quality metric for phylogenetic inference. A more complete genome suggests that the sample possesses common orthologs, and thus it is more likely to include the researcher's set of orthologs. By assessing genome completeness, systematic error due to taxa with low matrix occupancy can be avoided [20].

Each example above requires quality genome assemblies to generate accurate results. In order to understand how genome quality varies across taxa, we examined genome assembly quality in an exemplar group of organisms - butterflies - that has more than 600 published genomes. We evaluate the quality of these genomes using the Composite Quality Score (CQS), a metric that incorporates both N50 and BUSCO scores. The CQS can guide future studies seeking to utilize available genomes, as both aspects of quality are known to influence results.

Methods

We obtained all previously published genome assemblies and genomic reads of butterflies (Lepidoptera: Papilionoidea) from the NCBI SRA database [21]. In the case of genome assemblies, we searched NCBI using the taxonomy database (keywords Papilionoidea and papilionoid) for the latest assemblies, selecting the most recently submitted (as of October 2019; see Table S1). We also searched the SRA database [21] and published literature for available paired-end, whole-body, whole shotgun genome sequences of papilionoid species [16,22–33] (search terms butterfly genome; papilionoid genome; butterfly shotgun genome;

searches performed during October 2019).

We trimmed reads using TrimGalore requiring a quality score of 20 and read length of 30 (https://github.com/FelixKrueger/TrimGalore). We assembled reads using SPAdes v3.13 [34] using paired reads and allowing values of K to vary based on read length. For the majority of the *de-novo* genomes, 32 threads and 128 Gb of memory were sufficient. Forty genomes required additional memory; we ran these genomes with 24 threads with 720 Gb of memory, potentially due to deeper sequencing or greater genomic complexity. To assess assembly quality, we first used assembly-stats (https://github.com/sanger-pathogens/assembly-stats) to quantify the N50 for each assembly. This measure estimates the contiguity of assembly contigs and describes the contig length of half of the genome; i.e., 50% of the genome includes contigs greater than or equal to this length. We also used BUSCO v3.02 [4] to determine the presence of a set of 1,658 core insect single-copy genes which are highly conserved across insects and give an approximation of the completeness of the assembly. Herein, we evaluate only the BUSCO Complete score, which requires each of the 1,658 core ortholog genes in the assembly to include both start and stop codons. Theoretically, these 1,658 genes should be present in every insect taxon. We tested this assumption by confirming that all genes were present in at least one butterfly genome in our study using the BUSCO output. In order evaluate overall quality score, we utilize the Composite Quality Score (CQS), which log normalizes N50 and the number of complete BUSCO genes recovered (Table 1-2, S1-2).

Results

We assembled 606 papilionoid genomes from raw reads and downloaded 35 pre-assembled genomes from the SRA database [21]. These 641 butterfly samples with genomic data represent 624 unique taxa, because some species have replicate genomes (Table S1). We did not attempt to combine genomic reads from multiple conspecific individuals, as this will artificially increase heterozygosity and inevitably impact assembly quality [35]. All genomes

assembled for this study (Table S1) are available for download through NCBI (BioProject PRJNA606954) and quality statistics calculated for each genome are listed in Table S1.

Pre-assembled genomes from GenBank spanned six butterfly families and twelve subfamilies; our *de-novo* assembled genomes represent six families and twenty-four subfamilies (Figure 1). The only family for which no public genomic data was available was the Hedylidae, a species poor family with only 36 described Neotropical species [36]. Hesperiidae had the greatest number of species with available genomes (412), over half of which are in subfamily Pyrginae (249), largely due to research by Grishin and colleagues [16,22–29,32,33] (Figure 1). The Lycaenidae and Nymphalidae, two families with the greatest number of described species, have comparatively few species with available genomes (10 and 61, respectively; Figure 1). The metrics we used revealed large variance in genome quality. N50 and BUSCO scores are often similar (Figure 2), such that the highest quality genomes typically have high N50 and BUSCO scores, although not always the case (Table 1). These quality statistics measure two different aspects of quality and should be used in conjunction, as length distribution may not be associated with gene content [4]. However, our novel metric, Composite Quality Score (CQS), incorporates both aspects of quality enables researchers to evaluate both metrics simultaneously (Table S1).

Pre-assembled genomes downloaded from NCBI generally had high quality scores (Table S2, Figure 2) (N50 = 853,859; BUSCO = 79.9%; CQS = 80.08). Of these, four *Heliconius* genomes (*H. hecuba flava, H. hierax, H. wallacei,* and *H. xanthocles*) have notably lower mean quality scores (N50 = 914.25; BUSCO = 33.85%; CQS = 43). The CQS score enabled us to determine the highest and the lowest quality assemblies because it takes into account both quality metrics. The *Heliconius hierax* (GCA_900068475.1) genome had the lowest quality measures of the pre-assembled genomes we investigated (N50 = 916; BUSCO = 30.5; CQS = 42.46). The *Papilio xuthus* assembly (Figure 2A; GCA_000836235.1 [37]) had the highest quality scores of all genomes investigated (N50 = 6,198,915; BUSCO = 97.6%; CQS = 115.56).

Quality scores varied widely among the 606 shotgun *de-novo* genome assemblies (Figure 2). N50 ranged from 147 in *Katreus holocausta* (SRR9330403; Hesperiidae) to 43,033 in *Sertania guttata guttata* (Figure 2E; SRR10158585; Riodinidae). Fifty-two *de-novo* genomes resulted in a BUSCO score of 0% (Table S1), meaning that these genomes recovered none of the core insect orthologs. Seven had BUSCO scores of 90% or greater, with the greatest BUSCO score (96.1%) from *Papilio antimachus* (Figure 2D; SRR8954523 [31]). Overall, the mean quality scores of the *de-novo* genomes were low (N50 = 1732; BUSCO = 27.79%; CQS = 35.91). *Polygonia gigantea* (Figure 2G) had a nearly average N50, but zero insect core-orthologs recovered (N50 = 1601; BUSCO = 0%; CQS = 0). *Proboscis propylea* (Figure 2H) had a greater than average BUSCO score, but low N50 (N50 = 363; BUSCO = 45.1%; CQS = 39.00). In an effort to evaluate the variation in genome quality and identify the best exemplar genome for each major butterfly lineage, we present the highest quality genomes per subfamily (Table 1). In many cases, the highest quality genome had the highest N50 and the highest BUSCO, but in some cases one genome had a high N50, but low BUSCO (see above). In these cases, we report two genomes per subfamily, as future studies may need to prioritize one quality metric over the other. Table 2 summarizes the best butterfly genomes, regardless of taxonomy, based on CQS.

Surprisingly, we found that none of the pre-assembled genomes recovered all BUSCO insect genes (complete or fragmented). To understand whether these missing BUSCO insect orthologs were perhaps caused by a lineage-wide loss, we compared missing BUSCO genes from all butterfly genomes. We found that three insect genes (GDP dissociation inhibitor, EOG090W012Q; DEAD/DEAH box helicase domain, EOG090W05DF; and Organic solute transporter subunit alpha/Transmembrane protein 184, EOG090W08A5) were missing from all pre-assembled butterfly genomes, but present in many of our *de-novo* butterfly genomes.

Discussion

High-quality genomes are required for studies that span the biological sciences, from gene family, mutation research to macroevolutionary phylogenetics and population dynamics. Our results show that available genomes vary widely in quality and taxonomic coverage. The significant variance in N50 and BUSCO scores highlight an important message: not all genomes are similar in quality. Large-scale genomic studies, especially those that sequence species in an entire clade or geographic region represent great scientific feats, but if they are based on many low-quality genomes, they may not be useful for subsequent studies. We encourage peer-reviewed journals and public databases to require authors to report genome quality via N50, BUSCO, and Complete Quality Score (CQS), which can be accessioned with the assembly on NCBI as Global Statistics. Doing so provides maximum transparency, reproducibility, and a holistic view of future data reuse. We found that four core orthologs were absent from pre-assembled genomes on NCBI, but these orthologs were present in our *de-novo* assembled genomes; therefore, we encourage researchers to utilize our assemblies. While we are unable to determine the cause of the discrepancy, it does not seem likely that these core orthologs were lost in the butterfly lineage, and that BUSCO insect orthologs are generally useful for predicting genomic completeness. Presenting both meta-data and their metadata needs to become standard practice for genome science.

Our analyses highlight the extensive variation in the quality of genomes. Part of this discrepancy may be alleviated with changes in language. Perhaps we should begin referring to low quality genomes, such as *Katreus holocausta* (SRR9330403; N50 = 147; BUSCO = 0; CQS = 0) as ‘genomic data’, as opposed to the potentially misleading term, ‘genome’. Next, accessioning all assemblies would save countless hours of computation time and allow for the validation of results. In addition, assemblies would also allow results (e.g., gene family evolution, sequence identification, ortholog determination) from previous studies to be validated. We found that four pre-assembled *Heliconius* genomes had notably lower N50 and BUSCO scores, when compared to the average pre-assembled genome downloaded from NCBI. Quality

metrics of our *de-novo* assembled genomes were, in many cases, comparable to these four *Heliconius* genomes, suggesting that even low-quality genome assemblies can and should be accessioned. These *Heliconius* genomes are useful for the field, but users are not mislead into thinking the assembly is exceptionally high quality because accessioners include Scaffold N50 in Global Statistics for transparency. Including quality scores (as Global Statistics) for each draft assembly via the NCBI Assembly Database (in addition to taxon-specific genome databases, such as Lepbase [38], would provide a transparent overview of available genomes for future studies.

Assembling genomes requires considerable computational resources, and assessing genome quality simply from raw file size on GenBank can be misleading. Many studies in the biological and medical sciences rely on existing genomes and their annotations (e.g. [39]. If researchers independently assemble genomes, this can lead to duplicated effort and potential significant time investment. Further, if initial raw data quality is poor, the assemblies may not complete. Providing N50, BUSCO, CQS, as well as assemblies, in manuscripts and databases promotes transparency and discourages needless computation.

Contamination has been shown to be a pervasive pattern in genome and transcriptome sequencing projects, especially those that use multiplexed sequencing approaches [40–42]. In a recent study, Allio et al. [31] found that cross-contamination accounted for 0.26% of assembly contigs. While contaminants were removed from the study using CroCo [43], and thus do not impact their results, it remains unknown how much these contaminant sequences will impact future reusing these genomic data. This is because the authors did not accession genome assemblies, where contaminants were removed, and contaminants remain in accessioned reads. Further, it is impossible to repeat these necessary decontamination steps without detailed information regarding multiplex strategy [43]. Accessioning decontaminated assemblies to NCBI is a necessary and easy solution.

Our study reveals an incredible gap in knowledge: many genomic studies do not provide

genome assemblies and necessary quality metrics. Our main conclusions are:

1. A new quality metric, the Composite Quality Score (CQS), based on N50 and BUSCO scores is presented.
2. Draft assemblies and quality metrics for all 641 available butterfly genomes at the time of this study (available through associated GigaDB) (Table S1), including 50 genomes with highest quality scores are listed.
3. Long and contiguous reads, indicated by high N50 values, are one quality metric important to prioritize for all studies, but especially for studies of gene mutation, duplication or genomic architecture surrounding genes of interest.
4. Phylogenetic and CRISPR studies are strengthened when genomes with a high completeness score, such as BUSCO, are used.
5. We encourage researchers to present quality scores, and provide draft assemblies, in all genome publications and databases. Accessioning quality scores will enhance transparency and avoid unnecessary use of computational resources. Accessioning assemblies further promotes the FAIR Principles of interoperability and reuse by limiting contaminant sequences and allowing results to be confirmed.

Availability of supporting data

See Tables S1, S2 for genomic read accession numbers used in this study, and associated meta-data. The 606 genome assemblies produced using SPAdes v3.13 are available in the NCBI WGS repository, BioProject PRJNA606954.

Additional files

Table S1: TableS1Genome_denovo.xls

Table S2: TableS2_PreAssembledGenomes.xlsx

Abbreviations

CQS; Composite Quality Score

SRA; Sequence Read Archive

NCBI; National Center for Biotechnology Information

WGS; Whole Genome Shotgun

Competing interests

The authors declare that they have no competing interests.

Funding

This work was funded by the National Science Foundation Grants DEB #1541500 and #1557007 to AYK.

Acknowledgements

The authors acknowledge the University of Florida Research Computing (http://researchcomputing.ufl.edu) for providing computational resources and support that have contributed to the research results reported in this publication. We are grateful to Caroline Storer and Xuan-Kun Li, who provided helpful comments. Other members of the Kawahara Lab participated in thoughtful discussions that greatly improved the quality of this manuscript. We thank Andrew Warren, Laurel Kaminsky, Anupama Priyadarshini, Victoria Tran, and the FLMNH Digitization Team for providing butterfly images.

Authors' contributions

AYK conceived of the study. EAE performed data collection, data analysis, and produced the figures, with overall guidance from AYK. EAE and AYK wrote the manuscript.

References

1. Prum RO, Berv JS, Dornburg A, Field DJ, Townsend JP, Lemmon EM, et al. A comprehensive phylogeny of birds (Aves) using targeted next-generation DNA sequencing. *Nature*. 2015;526:569–73.

2. Zhang J, Cong Q, Shen J, Opler PA, Grishin NV. Genomics of a complete butterfly continent [Internet]. Available from: http://dx.doi.org/10.1101/829887

3. Thomas GWC, Dohmen E, Hughes DST, Murali SC, Poelchau M, Glastad K, et al. Gene content evolution in the arthropods. *Genome Biol*. 2020;21:15.

4. Simão FA, Waterhouse RM, Ioannidis P, Kriventseva EV, Zdobnov EM. BUSCO: assessing genome assembly and annotation completeness with single-copy orthologs. *Bioinformatics*. 2015;31:3210–2.

5. Edger PP, Heidel-Fischer HM, Bekaert M, Rota J, Glöckner G, Platts AE, et al. The butterfly plant arms-race escalated by gene and genome duplications. *Proc Natl Acad Sci*. 2015;112:8362–6.

6. Brown CA, Murray AW, Verstrepen KJ. Rapid expansion and functional divergence of subtelomeric gene families in yeasts. *Curr Biol.* 2010;20:895–903.

7. Gouin A, Bretaudeau A, Nam K, Gimenez S, Aury J-M, Duvic B, et al. Two genomes of highly polyphagous lepidopteran pests (*Spodoptera frugiperda*, Noctuidae) with different host-plant ranges. *Sci Rep.* 2017;7:11816.

8. Denton JF, Lugo-Martinez J, Tucker AE, Schrider DR, Warren WC, Hahn MW. Extensive error in the number of genes inferred from draft genome assemblies. *PLoS Comput Biol.* 2014;10:e1003998.

9. Casacuberta E, González J. The impact of transposable elements in environmental adaptation. *Mol Ecol.* 2013;22:1503–17.

10. Bennetzen JL. Transposable element contributions to plant gene and genome evolution. *Plant Mol Biol.* 2000;42:251–69.

11. Zhang B, Horvath S. A general framework for weighted gene co-expression network analysis. *Stat Appl Genet Mol Biol*. 2005;4:e17.

12. Cong L, Ran FA, Cox D, Lin S, Barretto R, Habib N, et al. Multiplex genome engineering using CRISPR/Cas systems. *Science*. 2013;339:819–23.

13. Xu W, Fu W, Zhu P, Li Z, Wang C, Wang C, et al. Comprehensive analysis of CRISPR/Cas9-mediated mutagenesis in by genome-wide sequencing. *Int J Mol Sci.* 2019;20:4125.

14. Chakrabarti AM, Henser-Brownhill T, Monserrat J, Poetsch AR, Luscombe NM, Scaffidi P. Target-specific precision of CRISPR-mediated genome editing. *Mol Cell.* 2019;73:699–713.e6.

15. Burrell AS, Disotell TR, Bergey CM. The use of museum specimens with high-throughput DNA sequencers. *J Hum Evol.* 2015;79:35–44.

16. Zhang J, Cong Q, Shen J, Brockmann E, Grishin NV. Genomes reveal drastic and recurrent phenotypic divergence in firetip skipper butterflies (Hesperiidae: Pyrrhopyginae). *Proc Biol Sci.* 2019;286:20190609.

17. Gilbert MTP, Moore W, Melchior L, Worobey M. DNA extraction from dry museum beetles without conferring external morphological damage. *PLoS One.* 2007;2:e272.

18. St Laurent RA, Mielke CGC, Herbin D, Dexter KM, Kawahara AY. A new target capture phylogeny elucidates the systematics and evolution of wing coupling in sack- bearer moths.

Syst Entomol. 2020;3:17.

19. Parham JF, Stuart BL, Bour R, Fritz U. Evolutionary distinctiveness of the extinct Yunnan box turtle (*Cuora yunnanensis*) revealed by DNA from an old museum specimen. *Proc Biol Sci.* 2004;271 Suppl 6:S391–4.

20. Sanderson MJ, McMahon MM, Steel M. Phylogenomics with incomplete taxon coverage: the limits to inference. *BMC Evol Biol.* 2010;10:155.

21. Leinonen R, Sugawara H, Shumway M, International Nucleotide Sequence Database Collaboration. The sequence read archive. *Nucleic Acids Res.* 2011;39:D19–21.

22. Zhang J, Cong Q, Shen J, Brockmann E, Grishin NV. Three new subfamilies of skipper butterflies (Lepidoptera, Hesperiidae). *Zookeys*. 2019;861:91–105.

23. Zhang J, Shen J, Cong Q, Grishin NV. Genomic analysis of the tribe Emesidini (Lepidoptera: Riodinidae). *Zootaxa*. 2019;4668:475–88.

24. Li W, Cong Q, Shen J, Zhang J, Hallwachs W, Janzen DH, et al. Genomes of skipper butterflies reveal extensive convergence of wing patterns. *Proc Natl Acad Sci U S A.* 2019;116:6232–7.

25. Cong Q, Shen J, Borek D, Robbins RK, Otwinowski Z, Grishin NV. Complete genomes of Hairstreak butterflies, their speciation, and nucleo-mitochondrial incongruence. *Sci Rep.* 2016;6:24863.

26. Cong Q, Li W, Borek D, Otwinowski Z, Grishin NV. The Bear Giant-Skipper genome suggests genetic adaptations to living inside yucca roots. *Mol Genet Genomics.* 2019;294:211–26.

27. Cong Q, Shen J, Li W, Borek D, Otwinowski Z, Grishin NV. The first complete genomes of

Metalmarks and the classification of butterfly families. *Genomics*. 2017;109:485–93.

28. Shen J, Cong Q, Borek D, Otwinowski Z, Grishin NV. Complete genome of *Achalarus lyciades*, The first representative of the Eudaminae subfamily of skippers. *Current Genomics.* 2017;18:366–74.

29. Shen J, Cong Q, Kinch LN, Borek D, Otwinowski Z, Grishin NV. Complete genome of *Pieris rapae*, a resilient alien, a cabbage pest, and a source of anti-cancer proteins. *F1000Research.* 2016;5:2631.

30. VanKuren NW, Massardo D, Nallu S, Kronforst MR. Butterfly mimicry polymorphisms highlight phylogenetic limits of gene reuse in the evolution of diverse adaptations. *Molecular Biology and Evolution.* 2019;36:2842–53.

31. Allio R, Scornavacca C, Benoit N, Clamens A-L, Sperling FAH, Condamine FL. Whole genome shotgun phylogenomics resolves the pattern and timing of swallowtail butterfly evolution. *Syst Biol*. 2019;69:38–60.

32. Cong Q, Shen J, Warren AD, Borek D, Otwinowski Z, Grishin NV. Speciation in Cloudless Sulphurs Gleaned from Complete Genomes. *Genome Biol Evol.* 2016;8:915–31.

33. Cong Q, Borek D, Otwinowski Z, Grishin NV. Skipper genome sheds light on unique phenotypic traits and phylogeny. *BMC Genomics*. 2015;16:639.

34. Bankevich A, Nurk S, Antipov D, Gurevich AA, Dvorkin M, Kulikov AS, et al. SPAdes: a new genome assembly algorithm and its applications to single-cell sequencing. *J Comput Biol.* 2012;19:455–77.

35. Kajitani R, Toshimoto K, Noguchi H, Toyoda A, Ogura Y, Okuno M, et al. Efficient de novo assembly of highly heterozygous genomes from whole-genome shotgun short reads. *Genome*

Res. 2014;24:1384–95.

36. Kawahara AY, Breinholt JW, Espeland M, Storer C, Plotkin D, Dexter KM, et al. Phylogenetics of moth-like butterflies (Papilionoidea: Hedylidae) based on a new 13-locus target capture probe set. *Mol Phylogenet Evol.* 2018;127:600–5.

37. Nishikawa H, Iijima T, Kajitani R, Yamaguchi J, Ando T, Suzuki Y, et al. A genetic mechanism for female-limited Batesian mimicry in *Papilio* butterfly. *Nat Genet*. 2015;47:405–9.

38. Challi RJ, Kumar S, Dasmahapatra KK, Jiggins CD, Blaxter M. Lepbase: the Lepidopteran genome database [Internet]. Available from: http://dx.doi.org/10.1101/056994

39. Venter JC, Adams MD, Myers EW, Li PW, Mural RJ, Sutton GG, et al. The sequence of the human genome. *Science.* 2001;291:1304–51.

40. Ballenghien M, Faivre N, Galtier N. Patterns of cross-contamination in a multispecies population genomic project: detection, quantification, impact, and solutions. *BMC Biology.* 2017;15:e25.

41. Jun G, Flickinger M, Hetrick KN, Romm JM, Doheny KF, Abecasis GR, et al. Detecting and estimating contamination of human DNA samples in sequencing and array-based genotype data. *Am J Hum Genet.* 2012;91:839–48.

42. Merchant S, Wood DE, Salzberg SL. Unexpected cross-species contamination in genome sequencing projects. *PeerJ.* 2014;2:e675.

43. Simion P, Belkhir K, François C, Veyssier J, Rink JC, Manuel M, et al. A software tool “CroCo” detects pervasive cross-species contamination in next generation sequencing data. *BMC Biol.* 2018;16:e28.

Tables 1 and 2 with captions below.

Taxonomy	Organism	Run	N50	BUSCO	CQS
Hesperiidae; Coeliadinae	Burara striata	SRR7174555	2096, n = 41313	C:90.6%[S:90.1%,D:0.5%],F:6.6%,M:2.8%	55.94088862
Hesperiidae; Eudaminae	Phocides pigmalion okeechobee	SRR7174453	9473, n = 12886	C:76.8%[S:76.5%,D:0.3%],F:19.1%,M:4.1%	65.46135929
Hesperiidae; Euschemoninae	Euschemon rafflesia rafflesia	SRR7174553	274, n = 42767	C:0.0%[S:0.0%,D:0.0%],F:0.1%,M:99.9%	0
Hesperiidae; Hesperiinae	Megathymus ursus violae	SRR7174358	23381, n = 5423	C:90.8%[S:90.3%,D:0.5%],F:7.2%,M:2.0%	73.60522707
Hesperiidae; Heteropterinae	Dalla quadristriga	SRR9330377	4229, n = 21585	C:69.9%[S:69.3%,D:0.6%],F:21.8%,M:8.3%	58.90947481
Hesperiidae; Pyrginae	Parelbella ahira ahira	SRR9330700	10910, n = 9466	C:84.7%[S:84.4%,D:0.3%],F:11.9%,M:3.4%	67.3814198
Hesperiidae; Trapezitinae	Toxidia parvulus	SRR9330370	929, n = 126609	C:21.8%[S:21.6%,D:0.2%],F:30.9%,M:47.3%	40.25363254
Lycaenidae; Polyommatinae	Cyclargus thomasi	SRR6727422	13909, n = 7389	C:91.4%[S:89.5%,D:1.9%],F:5.7%,M:2.9%	69.86777666
Lycaenidae; Theclinae	Calycopis cecrops	GCA_001625245.1	233537, n = 852	C:95.5%[S:93.8%,D:1.7%],F:2.1%,M:2.4%	91.06819032
Nymphalidae; Charaxinae	Charaxes varanes	SRR5175869	1435, n = 97758	C:49.6%[S:49.3%,D:0.3%],F:32.4%,M:18.0%	48.79035834
Nymphalidae; Danainae	Danaus plexippus	GCA_000235995.2	715714, n = 102	C:98.0%[S:96.1%,D:1.9%],F:1.0%,M:1.0%	99.66751795
Nymphalidae; Danainae	Danaus chryssipus	GCA_004959915.1	1465393, n = 63	C:93.9%[S:92.8%,D:1.1%],F:1.4%,M:4.7%	104.3586801
Nymphalidae; Heliconiinae	Heliconius melpomene melpomene	GCA_000313835.2	194302, n = 345	C:95.6%[S:95.1%,D:0.5%],F:1.8%,M:2.6%	89.72588585
Nymphalidae; Nymphalinae	Vanessa tameamea	GCA_002938995.1	2988984, n = 28	C:98.3%[S:97.9%,D:0.4%],F:0.8%,M:0.9%	110.2809423
Nymphalidae; Satyrinae	Bicyclus anynana	GCA_900239965.1	638282, n = 194	C:97.6%[S:96.8%,D:0.8%],F:0.8%,M:1.6%	98.76632831
Papilionidae; Baroniinae	Baronia brevicornis	SRR8954515	1644, n = 75290	C:58.9%[S:58.7%,D:0.2%],F:26.8%,M:14.3%	50.97552924
Papilionidae; Papilioninae	Papilio xuthus	GCA_000836235.1	6198915, n = 16	C:97.6%[S:96.3%,D:1.3%],F:1.0%,M:1.4%	115.5642765
Papilionidae; Parnassiinae	Sericinus montela	SRR8954536	2720, n = 58758	C:59.2%[S:58.8%,D:0.4%],F:29.0%,M:11.8%	54.4818178
Papilionidae; Parnassiinae	Parnassius honrathi	SRR8954539	4207, n = 70776	C:47.5%[S:47.3%,D:0.2%],F:31.5%,M:21.0%	55.64889409
Pieridae; Coliadinae	Phoebis sennae	GCA_001586405.1	299140, n = 267	C:91.1%[S:90.2%,D:0.9%],F:1.1%,M:7.8%	92.29739599
Pieridae; Dismorphiinae	Leptidea sinapis	GCA_900199445.1	112092, n = 1270	C:87.1%[S:86.1%,D:1.0%],F:7.9%,M:5.0%	84.58992605
Pieridae; Pierinae	Pieris rapae	GCA_001856805.1	617301, n = 118	C:98.0%[S:97.5%,D:0.5%],F:0.9%,M:1.1%	98.57389223
Riodinidae; Euselasiinae	Euselasia chrysippe	SRR10158562	1797, n = 73871	C:30.5%[S:29.9%,D:0.6%],F:33.2%,M:36.3%	46.65628804
Riodinidae; Nemeobiinae	Styx infernalis	SRR10158561	1716, n = 81313	C:19.9%[S:18.8%,D:1.1%],F:11.0%,M:69.1%	43.18889063
Riodinidae; Riodininae	Calephelis nemesis	GCA_002245505.1	206312, n = 1057	C:95.6%[S:95.1%,D:0.5%],F:3.0%,M:1.4%	90.16781074
Lycaenidae; Curetinae	Curetis bulis	SRR10158559	1098, n = 98830	C:28.3%[S:28.2%,D:0.1%],F:36.7%,M:35.0%	43.06507458
Hesperiidae; Pyrginae	Cecropterus (Achalarus?) lyciades	GCA_002930495.1	558064, n = 280	C:97.3%[S:96.9%,D:0.4%],F:1.2%,M:1.5%	97.73319179
Nymphalidae; Limentidinae	Limenitis arthemis	SRR1504973	629, n = 131257	C:12.6%[S:12.4%,D:0.2%],F:29.3%,M:58.1%	34.42386586

Table 1. Highest quality genomes by butterfly subfamily, according to N50, BUSCO, and CQS scores. Danainae and Parnassiinae subfamilies have two different assemblies that maximize N50 or BUSCO, so we provide both.

Unique ID	Organism	Score
GCA_000836235.1	Papilio xuthus	115.5642765
GCA_003671415.1	Megathymus ursus violae	112.7137774
GCA_003118415.2	Papilio memnon	112.5401019
GCA_000836215.1	Papilio polytes	110.7695026
GCA_002938995.1	Vanessa tameamea	110.2809423
GCA_004959915.1	Danaus chryssipus	104.3586801
GCA_001298355.1	Papilio machaon	102.9669769
GCA_008963455.1	Hypolimnas misippus	102.2409045
GCA_000235995.2	Danaus plexippus	99.66751795
GCA_900239965.1	Bicyclus anynana	98.76632831
GCA_001856805.1	Pieris rapae	98.57389223
GCA_002930495.1	Cecropterus lyciades	97.73319179
GCA_001278395.1	Lerema accius	96.98575776
GCA_001586405.1	Phoebis sennae	92.29739599
GCA_001625245.1	Calycopis cecrops	91.06819032
GCA_000931545.1	Papilio glaucus	90.98264566
GCA_002245505.1	Calephelis nemesis	90.16781074
GCA_000313835.2	Heliconius melpomene melpomene	89.72588585
GCA_002245475.1	Calephelis virginiensis	88.74279057
GCA_900199445.1	Leptidea sinapis	84.58992605
GCA_000716385.1	Melitaea cinxia	84.48140665
SRR7174358	Megathymus ursus violae	73.60522707
SRR4341246	Delias oraia	71.73605499
SRR8954516	Atrophaneura dixoni	70.46282949
SRR6727422	Cyclargus thomasi	69.86777666
SRR6727440	Eumaeus atala	68.89650343
SRR10158585	Sertania guttata guttata	67.92770324
SRR9330700	Parelbella ahira ahira	67.3814198
DRR087481	Eurema mandarina	65.93387282
SRR8883904	Heliconius erato	65.9287199
SRR8954523	Papilio antimachus	65.56841236
SRR7174453	Phocides pigmalion okeechobee	65.46135929
SRR8954525	Pachliopta kotzebuea	65.05965638
SRR7174410	Asbolis capucinus	64.96191264
SRR8548584	Papilio clytia	64.88052445
SRR8954535	Pharmacophagus antenor	64.75349617
SRR7174573	Cecropterus toxeus	63.83645166
SRR7174519	Erionota thrax	63.52290362
SRR9330717	Agara michaeli	63.44306122
SRR3102172	Heliconius congener	63.29553521
SRR3102337	Heliconius hewitsoni	63.14600995
SRR7174583	Molo mango	62.63386744
SRR7174357	Agathymus mariae mariae	62.2226772
SRR9330669	Myscelus amystis hages	62.20722151
SRR7174409	Calpodes ethlius	62.08680795
SRR7174434	Croniades pieria auraria	62.05052593
SRR7174352	Urbanus proteus proteus	61.95657365
SRR9330724	Jemadia pseudognetus	61.92601012
SRR9330671	Aspitha aspitha aspitha	61.86535105
SRR9330684	Mysoria ambigua	61.77179662

Table 2. Highest quality papilionoid genome assemblies, regardless of subfamily, ranked using CQS

Figure Captions

Figure 1. Pre-assembled and *de-novo* assembled genomes for each butterfly and subfamily shown on phylogeny of Espeland et al. (2018). Species-richness numbers estimates and are presented for comparison only.

Figure 2. Natural log normalized N50 and BUSCO scores plotted for both pre-assembled (black squares) and denovo (grey circles) genome assemblies. Letters correspond to inset images of representative species.

Additional files

Table S1. Sample ID, N50, BUSCO, CQS evaluation and sequencing metadata for *de-novo* assembled genomes.

Table S2. Sample ID, N50, BUSCO, CQS evaluation and sequencing metadata for pre-assembled genomes.

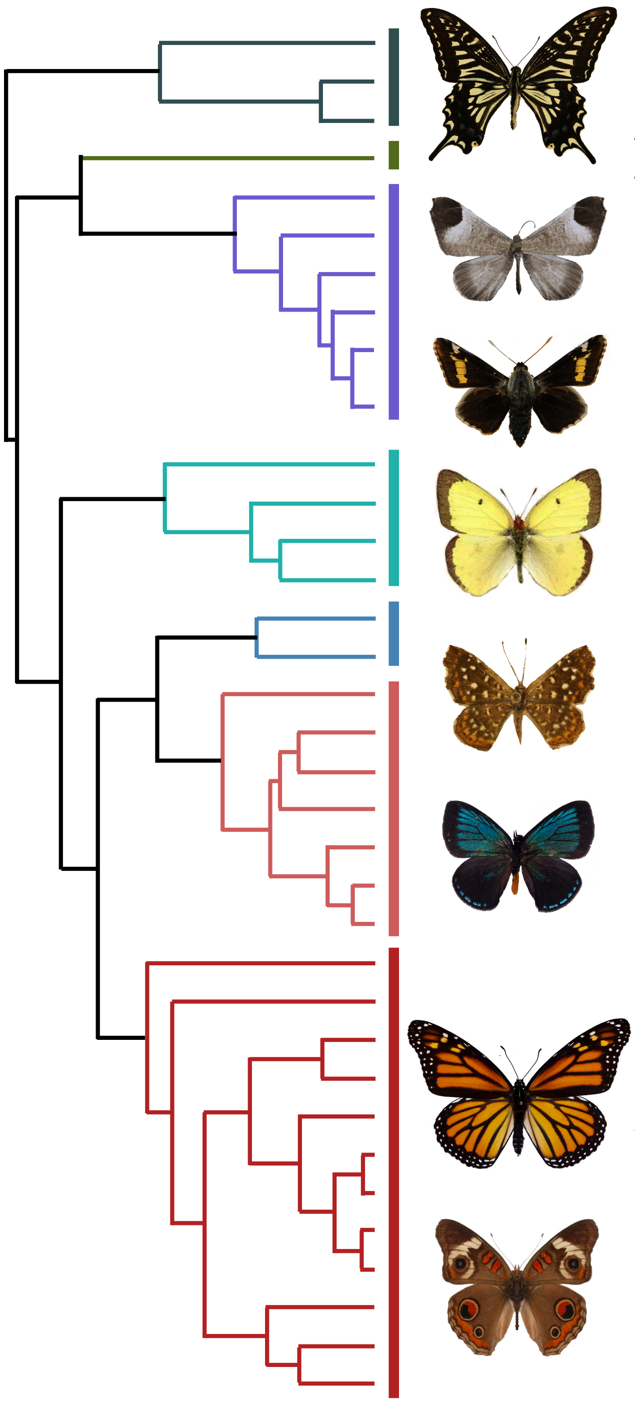

Family	Subfamily	~spp.	Genomes: Pre-Assemb.	Genomes: *De-novo*
Papilionidae (58)	Baroniinae	2	-	1
	Parnassiinae	600	-	13
	Papilioninae	1800	5	39
Hedylidae (0)	(Hedylidae)	36	-	-
Hesperiidae (412)	Coeliadinae	200	-	6
	Euschemoninae	2	-	1
	Eudaminae	600	-	92
	Pyrginae	2000	1	248
	Heteropterinae	300	-	13
	Trapezitinae	100	-	5
	Hesperiinae	3000	2	43
Pieridae (9)	Dismorphiinae	200	1	2
	Coliadinae	700	1	1
	Pseudopontiinae	5	-	-
	Pierinae	2600	1	3
Riodinidae (91)	Nemeobiinae	300	-	3
	Riodininae	1700	2	86
Lycaenidae (10)	Curetinae	60	-	1
	Aphnaeinae	280	-	-
	Portiinae	1000	-	-
	Miletinae	40	-	-
	Lycaeninae	300	-	-
	Theclinae	4200	1	2
	Polytommatinae	3300	-	6
Nymphalidae (61)	Libytheinae	35	-	-
	Danainae	1100	2	4
	Heliconiinae	1700	15	24
	Limenitidinae	2800	-	1
	Pseudergolinae	56	-	-
	Apaturinae	300	-	-
	Biblidinae	800	-	-
	Cyrestinae	200	-	-
	Nymphalinae	1300	3	6
	Calinaginae	26	-	-
	Charaxinae	1000	-	1
	Satyrinae	4700	1	4

Click here to download Figure Figure2_genome_021320_akedit.pdf

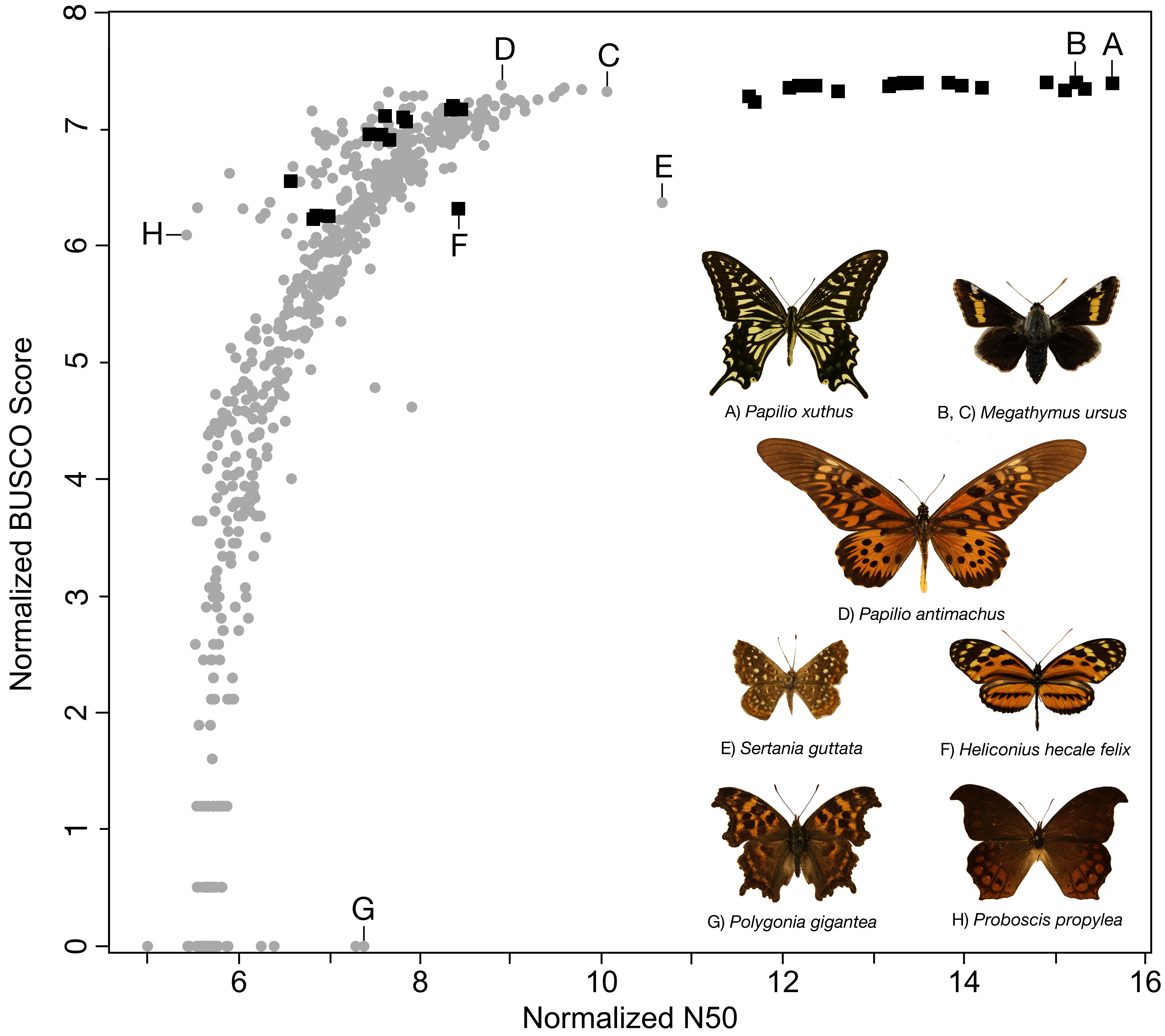

Supplementary Material Table S1

Click here to access/download
Supplementary Material
TableS1Genome_denovo-3.xls

Supplementary Material Table S2

Click here to access/download
Supplementary Material
TableS2_PreAssembledGenomes-2.xlsx

Cover letter Click here to download Personal Cover Cover_letter.doc

Florida Museum of Natural History
Center for Lepidoptera and Biodiversity

3215 Hull Rd.McGuire
Gainesville, FL 32611
352-273-2018

February 18, 2020

Dear Editor:

We are submitting our manuscript titled, "Genome Quality Variation Highlights the Importance of Transparency for Data Reuse". GigaScience is the ideal journal to publish this paper due to the readers' high interest in genome sequencing and reuse. Furthermore, the study advocates for the FAIR Principles and reproducibility in genome science, while providing a useful dataset of genome assemblies.

The dataset we provide in this manuscript includes genome assemblies for all 606 previously unpublished butterfly assemblies. We evaluate each of these *de-novo* genomes, as well as all other pre-assembled genomes, using quality control metrics. To do this, we provide N50, BUSCO, and a novel quality metric, the Composite Quality Score (CQS), which incorporates both N50 and BUSCO, allowing researchers to easily evaluate overall assembly quality. Additionally, we discuss potential re-use cases of the assemblies we provide. We discuss best-practices of genome science to promote the re-use of genomic data. In short, we advocate for the accession of all genome assemblies and quality score information, so that results are reproducible. Using our novel metric, we rank available genomes and identify the best quality butterfly genomes that could be useful for future studies.

Assemblies are unavailable for 95% (606/643) of butterfly genomes. The study was a large computational undertaking, and it makes these genomes available and accessible to many users that may not have the computational resources. Through our collection of these data, we found that genome quality varies widely. We discovered that some genomes are completely unusable due to low quality and low coverage.

Some genome applications may be more affected than others by low quality. As such, for each of the genome assemblies that we created, we provide and evaluate genome quality. **Our main message with this manuscript is to increase transparency in genome publishing protocols by highlighting the pitfalls of omitting genome assemblies and quality metrics.** To that end, we urge researchers to publish the assembly and associated quality metrics, along with the traditionally accessioned raw reads.

Our study is well suited for a Data Note in GigaScience. Specifically, our paper **(1) focuses on a particular dataset, and provides detailed methodology on data production, validation, and potential reuse**. The dataset we *produce* and disseminate here include genome assemblies for all 640 sequenced butterflies. We *validate* these previously unavailable assemblies using two different quality control metrics. We promote their *potential reuse* by discussing how genomics deepens existing research programs, and precautions that researchers might take.

Next, **(2) the paper supports and promotes the FAIR Principles (Findable, Accessible, Interoperable, and Reusable) for scientific data management and stewardship**. Here, we are addressing all important aspect of the FAIR Principles, allowing others to avoid unnecessary

computation, and addressing the fact that many researchers may not have the computational resources available to produce such a dataset.

This dataset has **(3) high reuse potential**. Not only will these assemblies be able to be used immediately for existing research programs, we also provide new potential research avenues. In this discussion, we highlight research avenues that might be most sensitive to particular quality metrics. Lastly, our data are **(4) extremely well-documented** and will set a new and higher standard for genome deposition. We provide **extensive metadata**, as well as provide two important quality metrics for all genome assemblies in our study. First, aggregating all available genomes into a single dataset is useful in and of itself, given that genomes may be accessioned on NCBI, with and without associated publications. It can be difficult for researchers to know what genomes are available. Further, it is currently impossible to determine the quality of a genome without producing an assembly (high computation time and resources) and associated quality metrics. Generally speaking, without these essential resources, it is impossible to verify results from published studies regarding genome evolution. Thus, the assembly and quality scores **add value to existing genomic datasets**.

Below, we provide suggested reviewers that are very knowledgeable in the field of genome science and evolutionary biology. None of them are in conflict with the authors of this paper. We suggest:

- Xin Zhou (China Agricultural University; Beijing, China)
- Andrew D. Young (California Department of Agriculture, Sacramento CA)
- Jessica P. Gillung (McGill University, Sainte-Anne-de-Bellevue, Canada)

The associated dataset (200 Gb) is uploaded to the NCBI WGS database (BioProject PRJNA606954). Thank you for considering *Genome Quality Variation Highlights the Importance of Transparency in Data Reuse* in GigaScience as a Data Note.

Sincerely,

Akito Y. Kawahara, Ph.D.

Associate Professor and Curator
Florida Museum of Natural History
University of Florida
Powell Hall, 3215 Hull Road
Gainesville, FL 32611-2710 USA
Tel: 352.273.2018 / Fax: 352.392.0479
Email: kawahara@flmnh.ufl.edu
http://www.flmnh.ufl.edu/mcguire/kawahara
