# Supplementary material for: De novo genome assemblies of butterflies
Source: Gigascience. 2021 Jun 2;10(6):giab041. doi: 10.1093/gigascience/giab041 (PMC8170690; doi:10.1093/gigascience/giab041)
Supplement: giab041_GIGA-D-20-00047_Revision_1 [file giab041_giga-d-20-00047_revision_1.pdf]

GigaScience

Resource announcement: De-novo genome assemblies of butterflies

--Manuscript Draft--

**Manuscript Number:**	GIGA-D-20-00047R1
**Full Title:**	Resource announcement: De-novo genome assemblies of butterflies
**Article Type:**	Data Note
**Funding Information:**	National Science Foundation (DEB 1541500) — Dr. Akito Y Kawahara<br>National Science Foundation (DEB 1557007) — Dr. Akito Y Kawahara
**Abstract:**	Abstract<br>Background: The current genomic age has led to the availability of thousands of genomes and enabled new advancements in biology. However, as the number of genomes increases, considerable attention should be given to their quality. Here we examine these trends in a taxonomically diverse and well-known group, butterflies, and provide draft, de-novo assemblies for all available butterfly genomes.<br><br>Findings : We provide de-novo assemblies for all 873 available butterfly genomes, interpret their quality, and provide general guidelines for future use. These assemblies will serve as a key resource for papilionoid genomics, especially for researchers without computational resources. We identify the 50 highest quality genomes across butterflies, and conclude that the ringlet Aphantopus hyperantus has the highest quality butterfly genome. Our post-processing of these draft genome assemblies identified 108 butterfly genomes that should not be reused due to contamination or extremely low quality. However, many draft genomes are of high utility, especially because permissibility of low-quality genomes is dependent on the objective of the study.<br><br>Conclusions: Quality metrics and assemblies are typically presented with annotated genome accessions, but rarely with de-novo genomes. We recommend that studies presenting genome sequences provide the assembly and some metrics of quality, as quality will significantly impact downstream results. Transparency in quality metrics are needed to improve the field of genome science and encourage data reuse.
**Corresponding Author:**	Emily A Ellis, Ph.D.<br>University of Florida<br>Gainesville, FL UNITED STATES
**Corresponding Author Secondary Information:**	
**Corresponding Author's Institution:**	University of Florida
**Corresponding Author's Secondary Institution:**	
**First Author:**	Emily A Ellis, Ph.D.
**First Author Secondary Information:**	
**Order of Authors:**	Emily A Ellis, Ph.D.<br>Akito Y Kawahara, PhD
**Order of Authors Secondary Information:**	
**Response to Reviewers:**	Dear Editor Zauner,<br><br>We are re-submitting this paper for review after incorporating feedback from the Editor and both Reviewers. We have revised the framing of the paper and adjusted the title of the paper to better fit a Resource Announcement. We thank both Reviewers for their insightful critiques and interest in our manuscript, which provides de-novo genome assemblies for all butterflies currently available, and evaluates the quality of genomes

Powered by Editorial Manager® and ProduXion Manager® from Aries Systems Corporation

in the field. The discussion of genome quality has been reframed in order to assist in the future use of these de-novo assemblies.

Both reviewers have pointed out the potential issues with creating a new quality score metric, so we have opted to remove mention of the CQS score from the manuscript and all supplementary material.

New Title: Resource announcement: De-novo genome assemblies of butterflies

Below, we respond to each Reviewer, in-line.

Thank you for the opportunity to revise our manuscript and respond to these important reviews. We look forward to your correspondence.

Emily Ellis and Akito Kawahara

Reviewer #1: Gigascience review

MS ID: GIGA-D-20-00047
Title: Genome quality variation highlights the importance of transparency for data reuse

The authors have collected a large dataset of whole genome sequencing reads from butterflies and assembled them using a unified pipeline, then analyzed the genomic quality that resulted. They then report the top 50 and bottom 50 genomes, based upon the standard N50 with BUSCO scores, and a Composite qualtity score (CQS), which is never clearly defined in the manuscript. In sum, the authors have used a lot of computational resources and generated genomes of potential use from minor datasets, to serve the research community.

Thank you for highlighting the utility of the resource that we provide. The CQS score was inadequately defined in the supplementary materials, and we apologize that it was not clear in the main body of the manuscript. However, after reading both reviews, we recognize that it may do more harm than good to the scientific community to introduce a new quality metric, especially one that combines two classically used metrics. As such, we have removed mention of the new score from the text, figures, tables, supplementary material, and our manuscript is stronger for it.

However, I am concerned about the presentation of their efforts. I find that arguing that studies generating genomic data should report quality metrics and more transparency, when the data generated was not intended for such work, problematic.

We appreciate this critique, because genomic data can have many uses, some that do not require an assembly at all. In these cases, we do not argue for their accession, but many results (synteny, gene family diversification, etc) do rely on a particular assembly. Not accessioning the particular assembly used creates an issue for results validation. By providing quality metrics and assemblies up-front (if they are used in the study), the future use of the resource can be more accurately judged. We have clarified the multitude of uses of genomic data in the text, see lines 179-184.

The work here would be more accessible to the community if the focus was presented as a mining of the existing genomic data, while acknowledging that the data was generated for a range of uses, the vast majority of which were never intended for genome assembly, but simply gene mining. Stated another way, the current line of argumentation does more harm than good. Please revise so that this can become a paper welcomed by the community.

We apologize that our original manuscript was not clear in this, our intentions were to provide accessible, mined genomic data and we have further clarified the manuscript to reflect this.

Additionally, the authors need to include currently available genomes, at least those published up through 2019. Currently they are missing many Heliconius and at least 4 Pierid genomes of high quality (e.g. Pieris rapae (Nallu et al. 2018), Pieris napi (Hill et

Powered by Editorial Manager® and ProduXion Manager® from Aries Systems Corporation

	al. 2019), Colias crocea (Woronik et al. 2019), Zerene cesonia (Rodriguez-Caro et al. 2020), Phoebis sennae (Grishin lab; Lepbase). Further, why did the authors not mine genomes off of LepBase, which is a good resource for many genome. Creating yet another database of genomes is problematic, so if you can at least include all of their genomes as well, or say that you did, that will allow you to generate a more inclusive database (Lepbase is no longer being maintained, but saying you are aware of it, and have mined everything off of it, will serve this paper well - and let people know you have included genomic resources Lepbase).<br><br>We wholeheartedly agree with this review in regards to avoiding the creation of yet another database. Thank you for raising this point. As such, we have deposited the genomes on an NCBI sub-database, the TPA (Third PArty) Database. It is our hope that this resource will be easily accessible.<br><br>The genomes of Colias crocea and Zerene cesonia were published after our manuscript was submitted to GigaScience. However, as our position is to provide a comprehensive resource on the state of butterfly genomes, we have updated the manuscript to reflect these new additions (see Supplemental Table S2). We thank the Reviewer for bringing these to our attention. We apologize for excluding these species, Lepbase was under construction leading up to the announcement of the cease of maintenance. We have also included all WGS sequences as of July 1, 2020.<br><br>I also recommend the authors cross reference with the genomes in Hill et al. 2019 (see Fig 3 and supplemental tables for data locations), which assessed genome quality across Lepidoptera and quantitatively assessed genome quality in light of contiguity and chromosomal structure, in a way much more thorough than BUSCOs.<br><br>Thank you for mentioning this important reference. We have cross-referenced the supplemental table and included the missing genomes into our study. We have also referenced this study in the Introduction, lines 52-53.<br><br>Once these issues are properly addressed, I do think this will make a positive contribution to the genomics community.<br><br>We thank the reviewer very much for their thoughtful comments and for recognizing the contribution of these genome assemblies for future use.<br><br><br>Major<br>The authors cite papers that are not peer-reviewed, and that should not be allowed, as such work may be fundamentally flawed. For example:<br>2. Zhang J, Cong Q, Shen J, Opler PA, Grishin NV. Genomics of a complete butterfly continent<br>[Internet]. Available from: http://dx.doi.org/10.1101/829887 [EDITOR'S NOTE: Actually, at GigaScience we do allow , even encourage, citation of preprints. If there is a published version, please cite the journal article, but if the material is only presented as a preprint, citing the preprint is totally fine with GigaScience policy].<br><br>We do not believe this manuscript is published yet and have kept the citation of the biorxiv work.<br><br>BUSCO mention in the introduction fails to discuss the importance of this for diploid genomic content, and this is significant omission.<br><br>Genome quality can also vary significantly in its haploid state, and this also need to be taken into account, but is not mentioned in the introduction<br><br>This is an important point, thank you. We have addressed this comment in the introduction, lines 42-45.<br><br>The authors seem to have entirely missed the online database for Lepidopteran genomes, called LepBase (http://ensembl.lepbase.org/index.html).<br><br>As stated above, Lepbase was unavailable from ~December to ~February (perhaps

Powered by Editorial Manager® and ProduXion Manager® from Aries Systems Corporation

intermittently), we apologize for its omission in this study. We have referenced this important database in-text, as well as included all genomes from the website into our study.

What is CQS? The authors state in their methods "the Composite Quality Score (CQS), which log normalizes N50 and the number of complete BUSCO genes recovered (Table 1-2, S1-2)." But this does not define how this metric is calculated …

The CQS entirely misses the duplicated content of the genome and this therefore not very informative as to genome quality.

Answered above; we have removed this metric from the manuscript.

I find this sentence confusing in the discussion "These Heliconius genomes are useful for the field, but users are not mislead into …" please restate for greater clarity.

Clarified; lines 183-187. Our point here was that although these particular Heliconius genomes are of significantly lower quality than other butterfly genomes on the Assembly database, they are still available and useful for future studies. Because quality metrics are listed in the Assembly database, they can be evaluated quickly by future users, who can then decide whether the quality is adequate in their particular use scenario.

Reviewer #2: Dear Authors

I enjoyed reading this paper because, like you, I also deeply care about assembly quality! Most people don't realise that a genome assembly is just an approximation and the quality of that approximation affects what one does and can do downstream. So thank you for addressing this issue.

Thank you very much for your kind comments, we are glad to hear that our message on genome quality came through in the manuscript. We feel that the term Genome more adequately represents data that is highly contiguous and well-resolved at the chromosomal level. Clarifying this point of language will move the field forward. We have further refined these points in the Discussion, lines 174-176.

I also deeply appreciate the sheer amount of effort and technical nous that has gone into collecting/collating/managing/running 600+ assemblies.

Thank you. We think that this resource will be useful for others in the field, so that they do not have to expend needless computational resources.

However, I have the following concerns:

1. What is the mathematical definition of CQS

I couldn't see the formula for how a CQS is calculated anywhere. Perhaps it was tucked away and I missed it? My apologies if so.

2. How is CQS better than just listing the N50 and Busco score?

One could argue that seeing the two numbers side by side is more useful as someone can choose to use a highly fragmented assembly as long as it is busco complete if all one wants to do is find orthologs for core genes (for a phylogeny, perhaps)

Thank you for raising this point, which was also raised by Reviewer 1. We have addressed this comment above, but to summarize: We now recognize that adding an additional quality metric into the field is less useful than we had previously thought. In fact, it may even complicate matters, since the metric does not incorporate new information on quality, but rather combines to classically used scores. As such, we have removed mention of this score.

3. What pre- or post- processing was done for the SPADES assemblies?

Powered by Editorial Manager® and ProduXion Manager® from Aries Systems Corporation

We have increased our discussion of the SPAdes assemblies and their processing in lines 101-112.

You have done a lot of work, and the protocol you describe (trim-galore + spades with varying k-mers) is technically accurate but I doubt anyone would actually do a genome assembly this way. They would do pre-filters for k-mers perhaps and assess the k-mer plots, or look for contaminants first using blobtoolkit etc

We agree with this comment and have used it to further clarify the manuscript, particularly in lines 213-215. The resource we provide is not intended to be the end-all-be-all for these species' genomes, but rather a starting point. We can imagine one practical use of these genome assemblies as a resource for researchers interested in a particular gene across butterflies, or orthology inference. In this use-case, the particular protocol we used would be sufficient, and we wholeheartedly agree that other use-cases may require a protocol that you describe.

In terms of post-processing, we have now included detailed methods on post-processing of these draft genomes. We also cite useful github pages, so that others can more easily accession their own assemblies to NCBI. During this project, we noticed that the requirements to submit assemblies to NCBI are unknown until after submission, which creates a lot of extra work for the submitter -- modifying and resubmitting assemblies with each new requirement. Our post-processing steps outlined will aid others in their own accessioning of assemblies.

--

Therefore, although I greatly appreciate the authors' efforts to make their data available and their processes transparent, as things stand right now, I feel this paper doesn't contribute much as a new method or as a new insight. Perhaps it would be better rewritten so that the focus is that of an opinion letter, or a resource announcement. Currently I feel the focus is the CQS metric and it is not as well described/justified as it could be.

Thank you for your helpful comments. We have opted to shift the focus to a resource announcement in the form of a Data Note.

Best regards
Sujai Kumar
The University of Edinburgh

Additional Information:	
**Question**	**Response**
Are you submitting this manuscript to a special series or article collection?	No
**Experimental design and statistics**<br><br>Full details of the experimental design and statistical methods used should be given in the Methods section, as detailed in our Minimum Standards Reporting Checklist. Information essential to interpreting the data presented should be made available in the figure legends.<br><br>Have you included all the information requested in your manuscript?	Yes

Powered by Editorial Manager® and ProduXion Manager® from Aries Systems Corporation

**Resources**<br><br>A description of all resources used, including antibodies, cell lines, animals and software tools, with enough information to allow them to be uniquely identified, should be included in the Methods section. Authors are strongly encouraged to cite Research Resource Identifiers (RRIDs) for antibodies, model organisms and tools, where possible.<br><br>Have you included the information requested as detailed in our Minimum Standards Reporting Checklist?	Yes
**Availability of data and materials**<br><br>All datasets and code on which the conclusions of the paper rely must be either included in your submission or deposited in publicly available repositories (where available and ethically appropriate), referencing such data using a unique identifier in the references and in the "Availability of Data and Materials" section of your manuscript.<br><br>Have you have met the above requirement as detailed in our Minimum Standards Reporting Checklist?	Yes

Manuscript

Click here to access/download;Manuscript;07202020_revision_GenomePaper

Abstract

Background: The current genomic age has led to the availability of thousands of genomes and enabled new advancements in biology. However, as the number of genomes increases, considerable attention should be given to their quality. Here we examine these trends in a taxonomically diverse and well-known group, butterflies, and provide draft, *de-novo* assemblies for all available butterfly genomes.

Findings: We provide *de-novo* assemblies for all 873 available butterfly genomes, interpret their quality, and provide general guidelines for future use. These assemblies will serve as a key resource for papilionoid genomics, especially for researchers without computational resources. We identify the 50 highest quality genomes across butterflies, and conclude that the ringlet *Aphantopus hyperantus* has the highest quality butterfly genome. Our post-processing of these draft genome assemblies identified 108 butterfly genomes that should not be reused due to contamination or extremely low quality. However, many draft genomes are of high utility, especially because permissibility of low-quality genomes is dependent on the objective of the study.

Conclusions: Quality metrics and assemblies are typically presented with annotated genome accessions, but rarely with *de-novo* genomes. We recommend that studies presenting genome sequences provide the assembly and some metrics of quality, as quality will significantly impact downstream results. Transparency in quality metrics are needed to improve the field of genome science and encourage data reuse.

Keywords (3-10)

Accessibility, Genomics, Life Sciences, Open Data, Papilionoidea

Introduction

The explosion of available genomes across the Tree of Life has created entirely new fields of science and is changing how we investigate long-standing questions in biology. Studies of gene family evolution and gene mutation have expanded from single genes to mapping the architecture of entire genomes. Macroevolutionary studies using genomic data are now regularly being generated at impressive scales, e.g. complete Class [1], continent [2], and spanning up to 500 million years [3]. As the scope of questions addressed with genomic data continues to expand, determining the impact of read length and genome completeness on results is vital. One metric that is often applied to assembled genomes is an N50 score, a weighted median statistic of contig continuity that describes the distribution of contig lengths. The N50 value indicates that half of the assembly is contained in contigs or scaffolds equal to or larger than the value. Assemblies with low N50s are more fragmented and the contigs or scaffolds have less overlap with one another. Completeness of a draft assembly can also be assessed using BUSCO scores [4]. This measure uses a taxonomically informed set of “core” protein-coding orthologs that are theoretically present in a given taxon to evaluate genomic completeness. BUSCO may detect both haplotypes sequenced from diploid tissue with adequate genome coverage. However, high heterozygosity can lead to more fragmented assemblies (low N50), potentially reducing the number of complete protein coding genes recovered. These scores can be influenced by biological variation, as in natural variation in chromosome length, or in lineage-wide loss of core orthologs, but also by systematic error, as in poor sequencing depth [4]. Genomes may be of low quality, and thus less useful for future studies, in terms of continuity, completeness, or a combination of these two metrics. Understanding how genomes with low quality metrics impact future results is of high importance.

Here, we provide draft *de-novo* genome assemblies for butterflies that will be useful for future studies. Previous studies have reviewed butterfly genomics [5,6], however, the last year

has seen a considerable increase of publicly available data, especially in the form of whole genome sequencing. In order to understand how genome quality varies across taxa, we examine genome assembly quality in this exemplar group of organisms that has more than 935 published genomes. Additionally, we explore potential uses of these data, bearing in mind their draft nature, and discuss the state of butterfly genomics in light of genome quality.

The resources we provide will be useful for those studying Lepidoptera evolution, gene discovery, and genomics, to name a few. Novel genes with important ecological implications arising through gene duplication can be identified, such as in plant detoxification [7]. Expansions of a particular gene copy are often indicative of functional adaptation (e.g. [8,9]), therefore inaccurate assessment of gene copy number will lead to false interpretations. Denton et al. [10] document a pattern of gene misassembly and false gene duplication rates in draft genomes, with gene number either over- or under-estimated in 40% of all gene families. The mechanism of such error is closely tied to N50, such that when genes are fragmented (low N50), and multiple contigs are assembled into non-biological contigs [10]. These types of errors will present as misidentification of gene duplication and loss, as well as non-biological mutations. Gene family evolution and mutation holds immense potential in uncovering the mechanisms behind rapid functional adaptation and potential subsequent speciation [11,12], and significant progress is being made in this area [3]. Including sequences of known identity to identify regions of sequencing artefacts or incorrect annotation, and implementing assembly error estimation [13], may mitigate these challenges.

Phylogenetic studies stand to gain enormous taxonomic ground into the 2020s, primarily due to the explosion of low-coverage genomes that are particularly well-suited for phylogenetic studies. Taxonomic coverage in phylogenetic studies is increasing exponentially with the ability to sequence genomes from historical or museum specimens. Advances in both cost and quality of sequencing, as well as the ability to sequence DNA from degraded museum samples [14–17] allows researchers to now produce phylogenies including all extant, and even extinct species in

a taxonomic group [18]. Stringency standards for including genomes in phylogenetic studies are not well established, and poor-quality genomes can produce erroneous assemblies of genes of interest, as detailed above [10]. Further, quality scores that highlight the completeness of the genome may serve an important quality-control step for the inclusion of genomes in phylogenies, and we recommend researchers to prioritize this quality metric for phylogenetic inference. A more complete genome suggests that the sample possesses common and complete protein coding genes, and thus it is more likely to include the researcher's set of orthologs. By assessing genome completeness, future systematic error due to taxa with low matrix occupancy may be avoided [19].

Despite the challenges that low quality and low coverage genomes present, the 873 *de-novo* genomes we provide can enhance existing research programs at no-cost.

Methods

We obtained all previously published genome assemblies and genomic reads of butterflies (Lepidoptera: Papilionoidea) from the NCBI [20] and Lepbase [21] databases as of July 1, 2020. In the case of NCBI genome assemblies, we searched using the taxonomy database (keywords Papilionoidea and papilionoid) for the latest assemblies, selecting the most recently submitted assembly, when multiple were available (as of July 1, 2019; see Table S1). We also searched the SRA database [20] and published literature for available paired-end, whole-body, whole shotgun genome sequences of papilionoid species [15,22–33] (search terms butterfly genome; papilionoid genome; butterfly shotgun genome; searches concluded on July 1, 2019).

We trimmed reads using TrimGalore requiring a quality score of 20 and read length of 30 [34]. We assembled reads using SPAdes v3.13 [35] using paired reads and allowing values of K to vary based on read length. For the majority of the *de-novo* genomes, 32 threads and 128 Gb of memory were sufficient. Forty genomes required additional memory; we ran these genomes

with 24 threads with 720 Gb of memory, potentially due to deeper sequencing or greater genomic complexity.

Following assembly, we performed several post-processing steps to ensure sequence integrity. First, we identified and removed contigs consisting of less than 200 base pairs using SeqTK [36]. We scanned for evidence of vector contamination using VecScan [37,38] and removed affected contigs. We used the NCBI contaminant screening database [38] to identify common contaminants, such as from fungi or bacteria, and then removed those contaminant sequences.

To assess assembly quality, we first used assembly-stats [39] to quantify the N50 for each cleaned, contaminant-free, assembly. This measure estimates the contiguity of assembly contigs and describes the contig length of half of the genome; i.e., 50% of the genome includes contigs greater than or equal to this length. We also used BUSCO v3.02 [4] to determine the presence of a set of 1,658 core insect single-copy genes (version 9) which are highly conserved across insects and give an approximation of the completeness of the assembly. Herein, we evaluate only the BUSCO Complete score, which requires each of the 1,658 core ortholog genes in the assembly to include both start and stop codons.

Results

We assembled 873 papilionoid genomes from raw reads and downloaded 62 pre-assembled genomes from the SRA database [20]. These 935 butterfly samples with genomic data represent 665 unique species, because some species have multiple subspecies sequenced or have replicate genomes (Table S1). We did not attempt to combine genomic reads from multiple conspecific individuals, as this will artificially increase heterozygosity and inevitably impact assembly quality [40]. All genomes assembled for this study (Table S1) are available for download through the TPA Database (SUB6977541 and SUB6977704) and quality

statistics calculated for each genome are listed in Table S1.

Pre-assembled genomes from GenBank span six butterfly families and twelve subfamilies; our *de-novo* assembled genomes represent six families and twenty-four subfamilies (Figure 1). The only family for which no public genomic data is available are the Hedylidae, a family with only 36 described Neotropical species [41]. Hesperiidae has the greatest number of species with available genomic data (473), over half of which are in subfamily Pyrginae (310), largely due to research by Grishin and colleagues [15,22–29,32,33] (Figure 1). The Nymphalidae, the family the most species-rich family of butterflies, has 287 genomes available, and 210 of these genomes are in the genus *Junonia* (subfamily Nymphalinae, Figure 1). The Lycaenidae has comparatively few genomes available (10), given its high species richness (Figure 1).

The metrics we used revealed large variance in genome assembly quality. N50 and BUSCO scores are often similar (Figure 2), such that the highest quality genomes typically have both high N50 and BUSCO scores, although not always the case (Table 1). These quality statistics measure two different aspects of quality and should be used in conjunction, as length distribution may not be associated with gene content [4].

Pre-assembled genomes downloaded from NCBI and Lepbase generally had high quality scores (Table S2, Figure 2) (N50 = 1,706,589; BUSCO = 81.2%). Of these, five *Heliconius* genomes (*H. hecuba flava, H. hierax, H. wallacei, H. xanthocles,* and *H. doris*) have notably lower mean quality scores (N50 = 996.6; BUSCO = 33.66%). The *Heliconius hierax* genome (GCA_900068475.1) had the lowest quality measures of the pre-assembled genomes we investigated (N50 = 916; BUSCO = 30.5). The satyrine *Aphantopus hyperantus* (GCA_902806685.1) had the highest quality scores of all genomes investigated (N50 = 15,230,192; BUSCO = 97.8%).

Quality scores varied widely among the draft *de-novo* genome assemblies (Figure 2). In fifty-one cases, we found that assemblies were comprised only of short (< 200bp) fragments

and contaminants. In these cases, we removed the assembly and report the N50 score as zero (Table 1). We did not further evaluate the quality of these assemblies. N50 ranged from 249 in *Junonia evarete nigrosuffusa* (SRR10765819; Nymphalidae) to 43,550 in *Sertania guttata guttata* (Figure 2E; SRR10158585; Riodinidae). Sixty-seven *de-novo* genomes resulted in a BUSCO score of 0% (Table S1), meaning that these genomes recovered none of the core insect orthologs. Seven had BUSCO scores of 90% or greater, with the greatest BUSCO score (96.4%) from *Papilio antimachus* (Figure 2D; SRR8954523 [31]). The mean quality scores of the *de-novo* genomes were low (N50 = 15,650; BUSCO = 28.25%; excluding zero values). *Proboscis propylea* (Figure 2H) had a greater than average BUSCO score, but low N50 (N50 = 605; BUSCO = 45.3%). In an effort to evaluate the variation in genome quality and identify the best exemplar genome for each major butterfly lineage, we present the highest quality genomes per subfamily (Table 1). Table 2 summarizes the fifty highest quality butterfly *de*-novo and pre-assembled genomes, regardless of taxonomy.

Discussion

High-quality genomes are required for studies that span the biological sciences, from gene and gene family diversification, to macroevolutionary phylogenetics and population dynamics. Our results show that available genomes vary widely in quality and taxonomic coverage. The significant variance in N50 and BUSCO scores highlight an important message: in the scientific literature, a “genome” can range from genomic fragments to fully annotated chromosomes. Large-scale genomic studies, especially those that sequence species in an entire clade or geographic region represent great scientific feats, but if they are based on low-quality genomes, they may not be useful for subsequent studies. Here, the original study may not even assemble the genomic reads, instead using a technique similar to Iterative Baited Assembly [42] to assemble particular genes directly from sequencing reads. However, in cases where an assembly is produced and the results rely on the particular assembly (e.g., gene

family evolution, sequence identification, ortholog determination), it is imperative to make it available. We found that five pre-assembled *Heliconius* genomes had notably lower N50 and BUSCO scores, when compared to the average pre-assembled genome downloaded from NCBI and Lepbase. Quality metrics of our *de-novo* assembled genomes were, in many cases, comparable to these five *Heliconius* genomes, suggesting that even low-quality genome assemblies can be accessioned. Including quality scores (as Global Statistics) for each draft assembly via the NCBI Assembly Database, would provide a transparent overview of available genomes for future studies. We encourage researchers to report genome quality via N50 and BUSCO, which can be accessioned with the assembly on NCBI as Global Statistics. Doing so provides maximum transparency, reproducibility, and a holistic view of future data reuse. In this way, users can easily evaluate whether the quality of the genome is high enough to investigate gene family diversification (prioritize N50) or phylogenetic systematics (prioritize BUSCO).

Contamination has been shown to be a pervasive pattern in genome and transcriptome sequencing projects, especially those that use multiplexed sequencing approaches [43–45]. In a recent study, Allio et al. [31] found that cross-contamination accounted for 0.26% of assembly contigs. While contaminants were removed from Allio et al. [31] using CroCo [46], and thus do not impact their results, it remains unknown how much these contaminant sequences will impact future studies that reuse these genomic data. The authors did not accession genome assemblies that had contaminants removed, and contaminants remain in accessioned reads. Further, it is impossible to repeat these necessary decontamination steps without detailed information regarding multiplex strategy [46]. Accessioning decontaminated assemblies to public databases is a necessary and easy solution.

Assembling genomes requires considerable computational resources and assessing genome quality simply from raw file size on GenBank can be misleading. Many studies in the biological and medical sciences rely on existing genomes and their annotations (e.g., [47]). If researchers independently assemble genomes, this can lead to duplicated effort and significant

time investment. In some cases, the genomic data fragments may not be intended to form an assembly, but providing further information on quality may clarify this. In our study, we found that at least fifty-one of the 873 genomes that we assembled are ultimately unusable, and another fifty-seven that we caution the reuse of (Table 1). These 108 samples produced assemblies that were either comprised entirely of contamination, contigs less than 200 bp, devoid of core insect genes, or a combination of these factors. An important caveat is that it is possible that alternate assembly methods would produce a greater quality genome assembly, and/or a targeted gene assembly approach could prove fruitful.

Our study provides draft assemblies and quality metrics for all 935 available butterfly genomes at the time of this study (available through NCBI TPA database) (Table S1), including 50 genomes with highest quality scores are listed. This resource can be used to enhance existing research programs by increasing the taxonomic breadth of gene mining studies. However, close attention should be paid to the quality of the genome assemblies that we provide, as low-quality genomes, in the form of low continuity or completeness, can influence future results.

Availability of supporting data

See Tables S1, S2 for genomic read accession numbers used in this study and associated meta-data. The 822 genome assemblies produced using SPAdes v3.13 are available in the NCBI TPA repository, SUB6977541 and SUB6977704.

Additional files

Table S1: TableS1Genome_denovo.xls

Table S2: TableS2_PreAssembledGenomes.xlsx

Abbreviations

bp; Base pair

SRA; Sequence Read Archive

NCBI; National Center for Biotechnology Information

TPA: Third PArty Database

Competing interests

The authors declare that they have no competing interests.

Funding

This work was funded by the National Science Foundation Grants DEB #1541500 and #1557007 to AYK.

Acknowledgements

The authors acknowledge the University of Florida Research Computing (http://researchcomputing.ufl.edu) for providing computational resources and support that have contributed to the research results reported in this publication. We are grateful to Caroline Storer and Xuan-Kun Li, who provided helpful comments. Other members of the Kawahara Lab participated in thoughtful discussions that greatly improved the quality of this manuscript. We thank Laurel Kaminsky, Anupama Priyadarshini, Victoria Tran, Andrew Warren, and the FLMNH Digitization Team for providing butterfly images.

Authors' contributions

AYK conceived of the study. EAE performed data collection, data analysis, and produced the figures, with overall guidance from AYK. EAE and AYK wrote the manuscript.

References

1. Prum RO, Berv JS, Dornburg A, Field DJ, Townsend JP, Lemmon EM, et al. A comprehensive phylogeny of birds (Aves) using targeted next-generation DNA sequencing. Nature. 2015;526:569–73.

2. Zhang J, Cong Q, Shen J, Opler PA, Grishin NV. Genomics of a complete butterfly continent [Internet]. Available from: http://dx.doi.org/10.1101/829887

3. Thomas GWC, Dohmen E, Hughes DST, Murali SC, Poelchau M, Glastad K, et al. Gene content evolution in the arthropods. Genome Biol. 2020;21:15.

4. Simão FA, Waterhouse RM, Ioannidis P, Kriventseva EV, Zdobnov EM. BUSCO: assessing genome assembly and annotation completeness with single-copy orthologs. Bioinformatics. 2015;31:3210–2.

5. Triant DA, Cinel SD, Kawahara AY. Lepidoptera genomes: current knowledge, gaps and future directions. Curr Opin Insect Sci. 2018;25:99–105.

6. Hill J, Rastas P, Hornett EA, Neethiraj R, Clark N, Morehouse N, et al. Unprecedented reorganization of holocentric chromosomes provides insights into the enigma of lepidopteran chromosome evolution. Sci Adv. 2019;5:eaau3648.

7. Edger PP, Heidel-Fischer HM, Bekaert M, Rota J, Glöckner G, Platts AE, et al. The butterfly plant arms-race escalated by gene and genome duplications. Proc Natl Acad Sci. 2015;112:8362–6.

8. Brown CA, Murray AW, Verstrepen KJ. Rapid expansion and functional divergence of subtelomeric gene families in yeasts. Curr Biol. 2010;20:895–903.

9. Gouin A, Bretaudeau A, Nam K, Gimenez S, Aury J-M, Duvic B, et al. Two genomes of highly polyphagous lepidopteran pests (*Spodoptera frugiperda*, Noctuidae) with different host-plant ranges. Sci Rep. 2017;7:11816.

10. Denton JF, Lugo-Martinez J, Tucker AE, Schrider DR, Warren WC, Hahn MW. Extensive error in the number of genes inferred from draft genome assemblies. PLoS Comput Biol. 2014;10:e1003998.

11. Casacuberta E, González J. The impact of transposable elements in environmental adaptation. Mol Ecol. 2013;22:1503–17.

12. Bennetzen JL. Transposable element contributions to plant gene and genome evolution. Plant Mol Biol. 2000;42:251–69.

13. Han MV, Thomas GWC, Lugo-Martinez J, Hahn MW. Estimating gene gain and loss rates in the presence of error in genome assembly and annotation using CAFE 3. Mol Biol Evol. 2013;30:1987–97.

14. Burrell AS, Disotell TR, Bergey CM. The use of museum specimens with high-throughput DNA sequencers. J Hum Evol. 2015;79:35–44.

15. Zhang J, Cong Q, Shen J, Brockmann E, Grishin NV. Genomes reveal drastic and recurrent

phenotypic divergence in firetip skipper butterflies (Hesperiidae: Pyrrhopyginae). Proc Biol Sci. 2019;286:20190609.

16. Gilbert MTP, Moore W, Melchior L, Worobey M. DNA extraction from dry museum beetles without conferring external morphological damage. PLoS One. 2007;2:e272.

17. St Laurent RA, Hamilton CA, Kawahara AY. Museum specimens provide phylogenomic data to resolve relationships of sack-bearer moths (Lepidoptera, Mimallonoidea, Mimallonidae). Systematic Entomology. 2018;43:729–61.

18. Parham JF, Stuart BL, Bour R, Fritz U. Evolutionary distinctiveness of the extinct Yunnan box turtle (*Cuora yunnanensis*) revealed by DNA from an old museum specimen. Proc Biol Sci. 2004;271 Suppl 6:S391–4.

19. Sanderson MJ, McMahon MM, Steel M. Phylogenomics with incomplete taxon coverage: the limits to inference. BMC Evol Biol. 2010;10:155.

20. Leinonen R, Sugawara H, Shumway M, International Nucleotide Sequence Database Collaboration. The sequence read archive. Nucleic Acids Res. 2011;39:D19–21.

21. Challi RJ, Kumar S, Dasmahapatra KK, Jiggins CD, Blaxter M. Lepbase: the Lepidopteran genome database [Internet]. Available from: http://dx.doi.org/10.1101/056994

22. Zhang J, Cong Q, Shen J, Brockmann E, Grishin NV. Three new subfamilies of skipper butterflies (Lepidoptera, Hesperiidae). Zookeys. 2019;861:91–105.

23. Zhang J, Shen J, Cong Q, Grishin NV. Genomic analysis of the tribe Emesidini (Lepidoptera: Riodinidae). Zootaxa. 2019;4668:475–88.

24. Li W, Cong Q, Shen J, Zhang J, Hallwachs W, Janzen DH, et al. Genomes of skipper butterflies reveal extensive convergence of wing patterns. Proc Natl Acad Sci U S A. 2019;116:6232–7.

25. Cong Q, Shen J, Borek D, Robbins RK, Otwinowski Z, Grishin NV. Complete genomes of Hairstreak butterflies, their speciation, and nucleo-mitochondrial incongruence. Sci Rep. 2016;6:24863.

26. Cong Q, Li W, Borek D, Otwinowski Z, Grishin NV. The Bear Giant-Skipper genome suggests genetic adaptations to living inside yucca roots. Molecular Genetics and Genomics. 2019;294:211–26.

27. Cong Q, Shen J, Li W, Borek D, Otwinowski Z, Grishin NV. The first complete genomes of Metalmarks and the classification of butterfly families. Genomics. 2017;109:485–93.

28. Shen J, Cong Q, Borek D, Otwinowski Z, Grishin NV. Complete genome of *Achalarus lyciades*, The first representative of the Eudaminae subfamily of skippers. Current Genomics. 2017;18:366–74.

29. Shen J, Cong Q, Kinch LN, Borek D, Otwinowski Z, Grishin NV. Complete genome of *Pieris rapae*, a resilient alien, a cabbage pest, and a source of anti-cancer proteins. F1000Research. 2016;5:2631.

30. VanKuren NW, Massardo D, Nallu S, Kronforst MR. Butterfly mimicry polymorphisms

highlight phylogenetic limits of gene reuse in the evolution of diverse adaptations. Molecular Biology and Evolution. 2019;36:2842–53.

31. Allio R, Scornavacca C, Benoit N, Clamens A-L, Sperling FAH, Condamine FL. Whole genome shotgun phylogenomics resolves the pattern and timing of swallowtail butterfly evolution. Syst Biol. 2019;69:38–60.

32. Cong Q, Shen J, Warren AD, Borek D, Otwinowski Z, Grishin NV. Speciation in Cloudless Sulphurs Gleaned from Complete Genomes. Genome Biol Evol. 2016;8:915–31.

33. Cong Q, Borek D, Otwinowski Z, Grishin NV. Skipper genome sheds light on unique phenotypic traits and phylogeny. BMC Genomics. 2015;16:639.

34. Krueger F. Trim galore [Internet]. 2015. Available from: https://github.com/FelixKrueger/TrimGalore

35. Bankevich A, Nurk S, Antipov D, Gurevich AA, Dvorkin M, Kulikov AS, et al. SPAdes: a new genome assembly algorithm and its applications to single-cell sequencing. J Comput Biol. 2012;19:455–77.

36. Li H. seqtk Toolkit for processing sequences in FASTA/Q formats. [Internet]. 2012. Available from: https://github.com/lh3/seqtk

37. National Center for Biotechnology Information. VecScreen [Internet]. 2009. Available from: https://www.ncbi.nlm.nih.gov/Web/Newsltr/Fall99/vecscreen.html

38. Tafer H. htafer/remoVecSec [Internet]. GitHub. 2018 [cited 2020 Jul 21]. Available from: https://github.com/htafer/remoVecSec

39. assembly-stats [Internet]. 2016. Available from: https://github.com/sanger-pathogens/assembly-stats

40. Kajitani R, Toshimoto K, Noguchi H, Toyoda A, Ogura Y, Okuno M, et al. Efficient de novo assembly of highly heterozygous genomes from whole-genome shotgun short reads. Genome Res. 2014;24:1384–95.

41. Kawahara AY, Breinholt JW, Espeland M, Storer C, Plotkin D, Dexter KM, et al. Phylogenetics of moth-like butterflies (Papilionoidea: Hedylidae) based on a new 13-locus target capture probe set. Mol Phylogenet Evol. 2018;127:600–5.

42. Breinholt JW, Earl C, Lemmon AR, Lemmon EM, Xiao L, Kawahara AY. Resolving Relationships among the Megadiverse Butterflies and Moths with a Novel Pipeline for Anchored Phylogenomics. Syst Biol. 2018;67:78–93.

43. Ballenghien M, Faivre N, Galtier N. Patterns of cross-contamination in a multispecies population genomic project: detection, quantification, impact, and solutions. BMC Biology. 2017;15:e25.

44. Jun G, Flickinger M, Hetrick KN, Romm JM, Doheny KF, Abecasis GR, et al. Detecting and estimating contamination of human DNA samples in sequencing and array-based genotype data. Am J Hum Genet. 2012;91:839–48.

45. Merchant S, Wood DE, Salzberg SL. Unexpected cross-species contamination in genome

sequencing projects. PeerJ. 2014;2:e675.

46. Simion P, Belkhir K, François C, Veyssier J, Rink JC, Manuel M, et al. A software tool “CroCo” detects pervasive cross-species contamination in next generation sequencing data. BMC Biol. 2018;16:e28.

47. Venter JC, Adams MD, Myers EW, Li PW, Mural RJ, Sutton GG, et al. The sequence of the human genome. Science. 2001;291:1304–51.

Table Captions

Table 1. Highest quality genomes by butterfly subfamily, according to N50 and BUSCO scores.

Table 2. Highest 50 quality papilionoid genome assemblies, regardless of subfamily, ranked using natural log normalized N50 and BUSCO Complete scores.

Figure Captions

Figure 1. Pre-assembled and *de-novo* assembled genomes for each butterfly and subfamily shown on phylogeny of Espeland et al. (2018). Species-richness numbers estimates and are presented for comparison only.

Figure 2. Natural log normalized N50 and BUSCO scores plotted for both pre-assembled (black squares) and *de-novo* (grey circles) genome assemblies. Letters correspond to inset images of representative species.

Additional files

Table S1. Sample ID, N50, BUSCO, and sequencing metadata for *de-novo* assembled genomes.

Table S2. Sample ID, N50, BUSCO, and sequencing metadata for pre-assembled genomes.

Click here to access/download;Table;Table 1 best in subfamily July 16 2020.pdf

Taxonomy	Organism	Accession ID	N50	BUSCO (C%)
Hesperiidae; Coeliadinae	*Choaspes benjaminii*	SRR7174556	2532	87.3
Hesperiidae; Eudaminae	*Phocides pigmalion*	SRR7174453	9497	76.7
Hesperiidae; Hesperiinae	*Megathymus ursus*	GCA_003671415.1	4153133	98.3
Hesperiidae; Heteropterinae	*Dalla quadristriga*	SRR9330377	4259	69.8
Hesperiidae; Pyrginae	*Cecropterus lyciades*	GCA_002930495.1	558064	97.3
Hesperiidae; Trapezitinae	*Toxidia parvulus*	SRR9330370	932	21.7
Lycaenidae; Curetinae	*Curetis bulis*	SRR10158559	1108	28.3
Lycaenidae; Polyommatinae	*Cyclargus thomasi*	SRR6727422	13909	91.3
Lycaenidae; Theclinae	*Calycopis cecrops*	GCA_001625245.1	233537	95.5
Nymphalidae; Charaxinae	*Charaxes varanes*	SRR5175869	1531	49.5
Nymphalidae; Danainae	*Danaus plexippus*	GCA_009731565.1	9209872	98
Nymphalidae; Heliconiinae	*Heliconius erato*	LepBase_Heliconius_era	10688973	97.4
Nymphalidae; Limentidinae	*Limenitis arthemis*	SRR1504973	631	12.6
Nymphalidae; Morphinae	*Taenaris catops*	GCA_009936525.1	1720500	35.2
Nymphalidae; Nymphalinae	*Vanessa tameamea*	GCA_002938995.1	2988984	98.3
Nymphalidae; Satyrinae	*Aphantopus hyperantu*	GCA_902806685.1	15230192	97.8
Papilionidae; Baroniinae	*Baronia brevicornis*	SRR8954515	1886	59
Papilionidae; Papilioninae	*Papilio xuthus*	GCA_000836235.1	6198915	97.6
Papilionidae; Parnassiinae	*Sericinus montela*	SRR8954536	3584	59.4
Pieridae; Coliadinae	*Zerene cesonia*	GCA_012273895.1	9214832	95.6
Pieridae; Dismorphiinae	*Leptidea sinapis*	GCA_900199415.2	857189	97.2
Pieridae; Pierinae	*Pieris napi*	LepBase_Pieris_napi_v1	12597868	94.4
Riodinidae; Nemeobiinae	*Euselasia chrysippe*	SRR10158562	1806	30.3
Riodinidae; Riodininae	*Calephelis nemesis*	GCA_002245505.1	206312	95.6

 Click here to access/download;Table;Table2 top 50 genomes.pdf

Rank	Accession ID	Organism	N50	BUSCO (C%)
1	GCA_902806685.1	*Aphantopus hyperantus*	15230192	97.8
2	LepBase_Pieris_napi_v1.1	*Pieris napi*	12597868	94.4
3	LepBase_Heliconius_erato_demophoon	*Heliconius erato*	10688973	97.4
4	GCA_009731565.1	*Danaus plexippus*	9209872	98
5	GCA_012273895.1	*Zerene cesonia*	9214832	95.6
6	GCA_000836235.1	*Papilio xuthus*	6198915	97.6
7	GCA_011763625.1	*Papilio bianor*	13111833	65
8	GCA_003671415.1	*Megathymus ursus*	4153133	98.3
9	GCA_003118415.2	*Papilio memnon*	4560862	92.9
10	GCA_000836215.1	*Papilio polytes*	3672263	91.8
11	GCA_002938995.1	*Vanessa tameamea*	2988984	98.3
12	LepBase_Junonia_coenia_JC_v1.0	*Junonia coenia*	1571165	98.2
13	GCA_004959915.1	*Danaus chryssipus*	1465393	93.9
14	GCA_001298355.1	*Papilio machaon*	1174287	95.5
15	GCA_008963455.1	*Hypolimnas misippus*	1011763	98.1
16	GCA_900199415.2	*Leptidea sinapsis*	857189	97.2
17	GCA_010014825.1	*Danaus melanippus*	889656	89.4
18	GCA_900239965.1	*Bicyclus anynana*	638282	97.6
19	GCA_001856805.1	*Pieris rapae*	617301	98
20	GCA_013186455.1	*Papilio dardanus*	596599	94.3
21	GCA_002930495.1	*Cecropterus lyciades*	558064	97.3
22	GCA_001278395.1	*Lerema accius*	525349	95.1
23	GCA_001586405.1	*Phoebis sennae*	299140	91.1
24	GCA_009936525.1	*Taenaris catops*	1720500	35.2
25	GCA_001625245.1	*Calycopis cecrops*	233537	95.5
26	GCA_000931545.1	*Papilio glaucus*	230841	95.5
27	GCA_002245505.1	*Calephelis nemesis*	206312	95.6
28	GCA_010014985.1	*Delias pasithoe*	193720	96.5
29	GCA_000313835.2	*Heliconius melpomene*	194302	95.6
30	GCA_009667785.1	*Maniola jurtina*	212945	88.3
31	GCA_002245475.1	*Calephelis virginiensis*	175106	93.9
32	LepBase_Heliconius_burneyi_helico3	*Heliconius burneyi*	106325	96.5
33	GCA_000716385.1	*Melitaea cinxia*	119328	83
34	GCA_009982905.1	*Colias croceus*	95765	92.5
35	LepBase_Heliconius_hecalesia_helico3	*Heliconius hecalesia*	68855	96.5
36	Lepbase_Heliconius_demeter_helico3	*Heliconius demeter*	67995	96.8
37	LepBase_Heliconius_besckei_helico3	*Heliconius besckei*	64778	95.8
38	LepBase_Heliconius_himera_helico3	*Heliconius himera*	48684	96.5
39	LepBase_Heliconius_sara_helico3	*Heliconius sara*	43390	94.3
40	LepBase_Heliconius_telesiphe_helico3	*Heliconius telesiphe*	42672	94.7
41	LepBase_Eueides_tales_helico3	*Eueides tales*	32552	94.7
42	SRR7174358	*Megathymus ursus*	24120	90.7
43	LepBase_Agraulis_vanillae_helico3	*Agraulis vanillae*	21413	94.6
44	LepBase_Dryas_iulia_helico3	*Dryas iulia*	21916	92.3
45	SRR4341246	*Delias oraia*	18269	92.3
46	GCA_900499025.1	*Pararge aegeria*	16525	88
47	SRR8954516	*Atrophaneura dixoni*	14618	93.5
48	SRR6727422	*Cyclargus thomasi*	13909	91.3
49	SRR6727440	*Eumaeus atala*	13611	87
50	SRR10158585	*Sertania guttata*	43550	35.1

Figure 1
Click here to access/download;Figure;figure1_072120.pdf

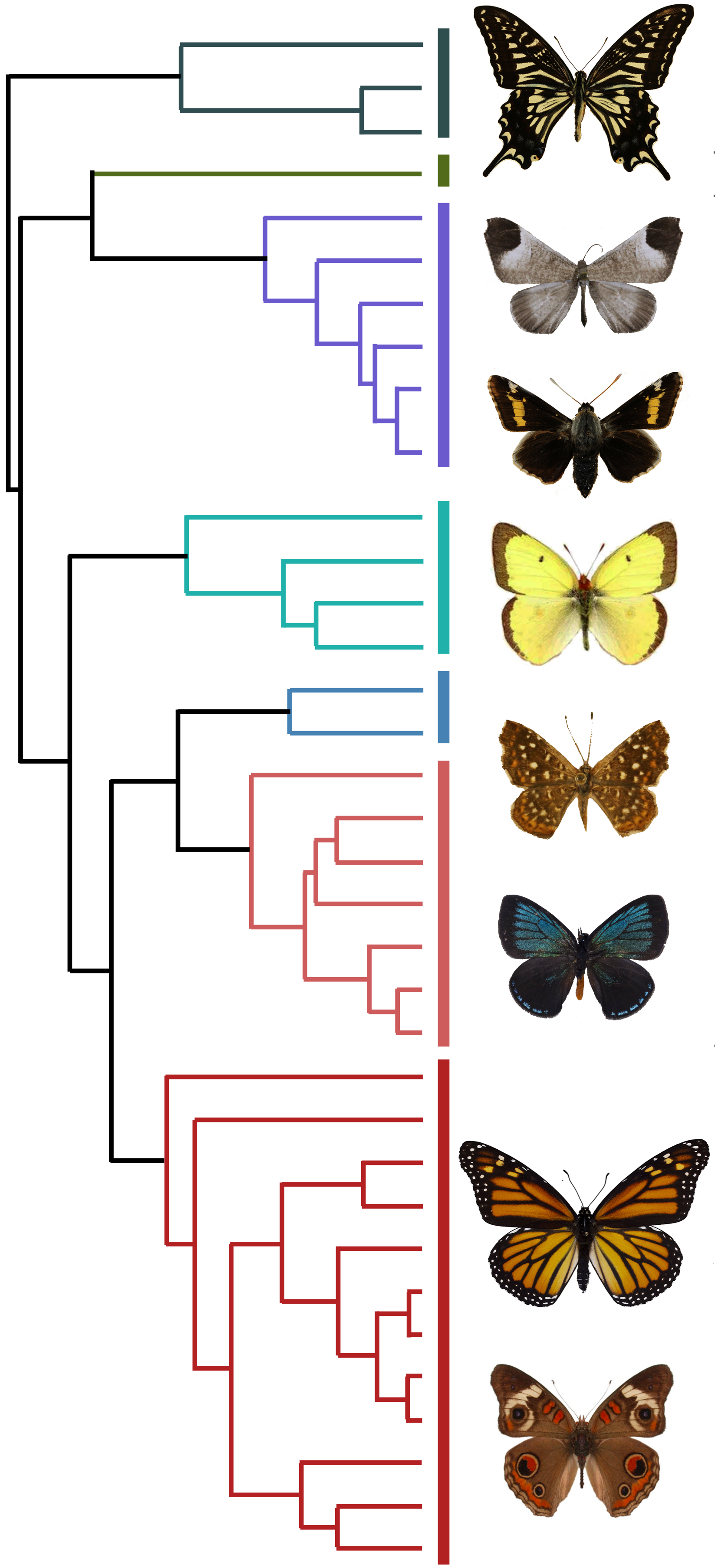

Family	Subfamily	~spp.	Genomes: Pre-Assemb.	Genomes: *De-novo*
Papilionidae (61)	Baroniinae	2	-	1
	Parnassiinae	600	13	13
	Papilioninae	1800	8	39
Hedylidae (0)	(Hedylidae)	36	-	-
Hesperiidae (472)	Coeliadinae	200	-	6
	Euschemoninae	2	-	1
	Eudaminae	600	-	92
	Pyrginae	2000	1	309
	Heteropterinae	300	-	13
	Trapezitinae	100	-	5
	Hesperiinae	3000	2	43
Pieridae (13)	Dismorphiinae	200	1	2
	Coliadinae	700	1	3
	Pseudopontiinae	5	-	-
	Pierinae	2600	3	3
Riodinidae (91)	Nemeobiinae	300	-	3
	Riodininae	1700	2	86
Lycaenidae (10)	Curetinae	60	-	1
	Aphnaeinae	280	-	-
	Portiinae	1000	-	-
	Miletinae	40	-	-
	Lycaeninae	300	-	-
	Theclinae	4200	1	2
	Polytommatinae	3300	-	6
Nymphalidae (287)	Libytheinae	35	-	-
	Danainae	1100	3	4
	Heliconiinae	1700	28	24
	Limenitidinae	2800	-	1
	Pseudergolinae	56	-	-
	Apaturinae	300	-	-
	Biblidinae	800	-	-
	Cyrestinae	200	-	-
	Nymphalinae	1300	5	212
	Calinaginae	26	-	-
	Charaxinae	1000	-	1
	Satyrinae	4700	4	4

Figure 2

Click here to access/download;Figure;Rplot.qualityscore_071620

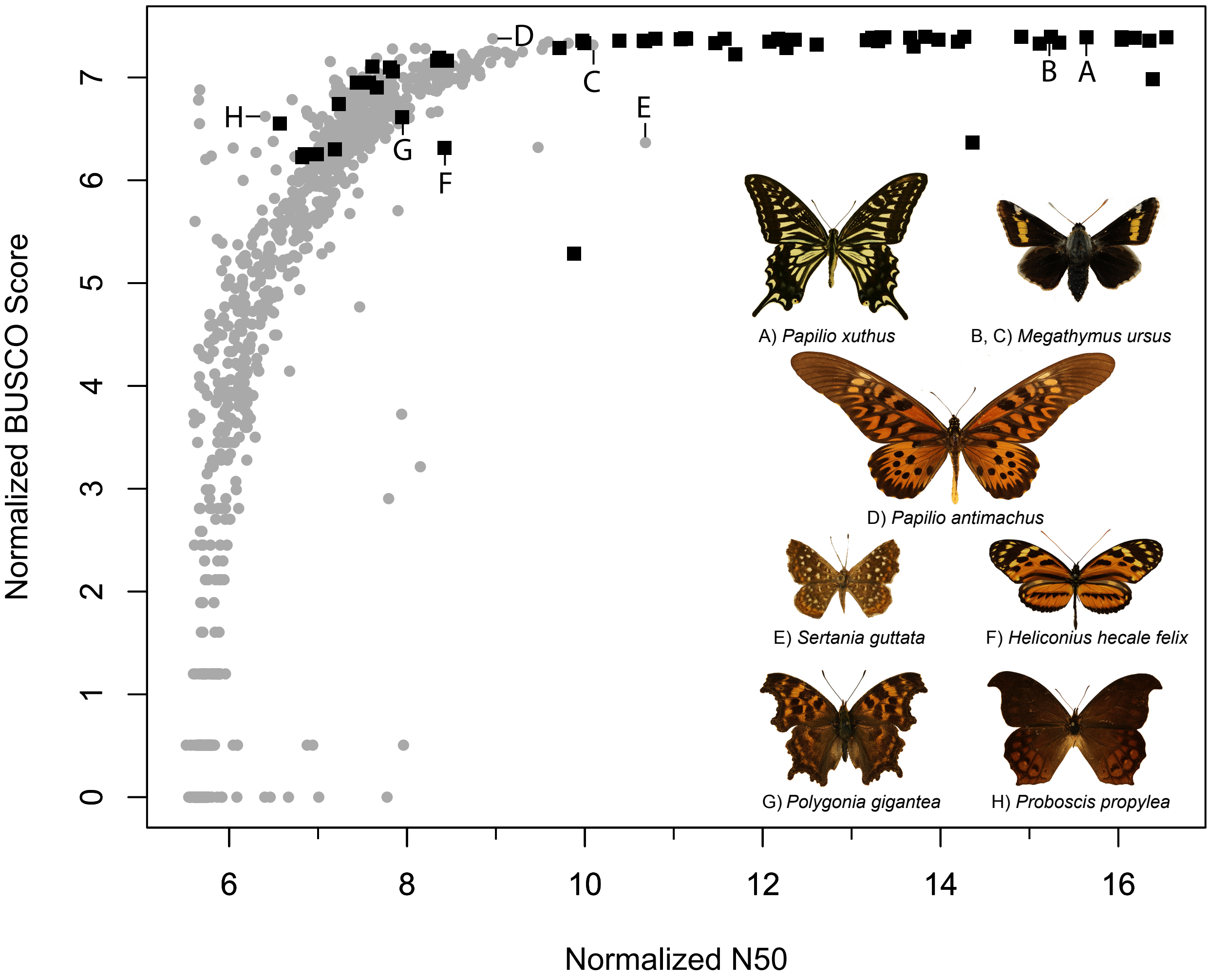

Supplementary Material Table S1

Click here to access/download
Supplementary Material
TableS1Genome_071520.xls

Supplementary Material Table S2

Click here to access/download
Supplementary Material
TableS2_PreAssembledGenomes_071620.xls
