# Supplementary material for: De novo genome assemblies of butterflies
Source: Gigascience. 2021 Jun 2;10(6):giab041. doi: 10.1093/gigascience/giab041 (PMC8170690; doi:10.1093/gigascience/giab041)
Supplement: giab041_GIGA-D-20-00047_Revision_4 [file giab041_giga-d-20-00047_revision_4.pdf]

GigaScience

De-novo genome assemblies of butterflies

--Manuscript Draft--

**Manuscript Number:**	GIGA-D-20-00047R4
**Full Title:**	De-novo genome assemblies of butterflies
**Article Type:**	Data Note
**Funding Information:**	National Science Foundation (DEB 1541500) — Dr. Akito Y Kawahara<br>National Science Foundation (DEB 1557007) — Dr. Akito Y Kawahara
**Abstract:**	Abstract<br>Background: The current genomic age has led to the availability of thousands of genomes and enabled new advancements in biology. However, as the number of genomes increases, considerable attention should be given to their quality. Here we examine these trends in a taxonomically diverse and well-known group, butterflies, and provide draft, de-novo assemblies for all available butterfly genomes.<br><br>Findings : We provide de-novo assemblies for all 873 available butterfly genomes, interpret their quality, and provide general guidelines for future use. These assemblies will serve as a key resource for papilionoid genomics, especially for researchers without computational resources. We identify the 50 highest quality genomes across butterflies, and conclude that the ringlet Aphantopus hyperantus has the highest quality butterfly genome. Our post-processing of these draft genome assemblies identified 108 butterfly genomes that should not be reused due to contamination or extremely low quality. However, many draft genomes are of high utility, especially because permissibility of low-quality genomes is dependent on the objective of the study.<br><br>Conclusions: Quality metrics and assemblies are typically presented with annotated genome accessions, but rarely with de-novo genomes. We recommend that studies presenting genome sequences provide the assembly and some metrics of quality, as quality will significantly impact downstream results. Transparency in quality metrics are needed to improve the field of genome science and encourage data reuse.
**Corresponding Author:**	Emily A Ellis, Ph.D.<br>University of Florida<br>Gainesville, FL UNITED STATES
**Corresponding Author Secondary Information:**	
**Corresponding Author's Institution:**	University of Florida
**Corresponding Author's Secondary Institution:**	
**First Author:**	Emily A Ellis, Ph.D.
**First Author Secondary Information:**	
**Order of Authors:**	Emily A Ellis, Ph.D.<br>Caroline G Storer, PhD<br>Akito Y Kawahara, PhD
**Order of Authors Secondary Information:**	
**Response to Reviewers:**	Dear Editor Zauner,<br><br>All assemblies associated with this Data Note are now uploaded and accepted into the NCBI TPA Database under the BioProject PRJNA606954. These assemblies do not have an associated accession number, instead the assembly is linked to the proper

Powered by Editorial Manager® and ProduXion Manager® from Aries Systems Corporation

	BioSample ID. So that you and the reviewers can see how this works, I released 35 assemblies and ready to release the remaining 787 assemblies.<br><br>Thank you very much for all of your help with this manuscript.<br><br>All my best,<br>Emily<br>I am ready to release all assemblies,
**Additional Information:**	
**Question**	**Response**
Are you submitting this manuscript to a special series or article collection?	No
**Experimental design and statistics**<br><br>Full details of the experimental design and statistical methods used should be given in the Methods section, as detailed in our Minimum Standards Reporting Checklist. Information essential to interpreting the data presented should be made available in the figure legends.<br><br>Have you included all the information requested in your manuscript?	Yes
**Resources**<br><br>A description of all resources used, including antibodies, cell lines, animals and software tools, with enough information to allow them to be uniquely identified, should be included in the Methods section. Authors are strongly encouraged to cite Research Resource Identifiers (RRIDs) for antibodies, model organisms and tools, where possible.<br><br>Have you included the information requested as detailed in our Minimum Standards Reporting Checklist?	Yes
**Availability of data and materials**<br><br>All datasets and code on which the conclusions of the paper rely must be either included in your submission or	Yes

deposited in publicly available repositories (where available and ethically appropriate), referencing such data using a unique identifier in the references and in the "Availability of Data and Materials" section of your manuscript.<br><br>Have you have met the above requirement as detailed in our Minimum Standards Reporting Checklist?	

Powered by Editorial Manager® and ProduXion Manager® from Aries Systems Corporation

Manuscript Click here to access/download;Manuscript;Butterfly-assemblies_Forproduction.docx

De novo genome assemblies of butterflies

Emily A Ellis*, Caroline G. Storer, Akito Y. Kawahara

Florida Museum of Natural History, University of Florida

Powell Hall, 3215 Hull Road Gainesville, FL 32611-2710 USA

*Corresponding author: Emily A Ellis emilyellis13@gmail.com, ORCID 0000-0003-3343-0923

Abstract

Background: The current genomic age has led to the availability of thousands of genomes and enabled new advancements in biology. However, as the number of genomes increases, considerable attention should be given to their quality. Here we examine these trends in a taxonomically diverse and well-known group, butterflies, and provide draft, *de-novo* assemblies for all available butterfly genomes. Due to massive genome sequencing investment and taxonomic curation, this is an excellent group to explore genome quality.

Findings: We provide *de-novo* assemblies for all 822 available butterfly genomes and interpret their quality in terms of completeness and continuity. Further, we discuss reuse cases and provide general guidelines in these cases. These assemblies will serve as a key resource for papilionoid genomics, especially for researchers without computational resources. We identify the 50 highest quality genomes across butterflies, and conclude that the ringlet, *Aphantopus hyperantus* has the highest quality papilionoid genome. Our post-processing of these draft genome assemblies identified 118 butterfly genomes that should not be reused due to contamination or extremely low quality. However, many draft genomes are of high utility, especially because permissibility of low-quality genomes is dependent on the objective of the study.

Conclusions: Quality metrics and assemblies are typically presented with annotated genome accessions, but rarely with *de-novo* genomes. We recommend that studies presenting genome sequences provide the assembly and some metrics of quality, as quality will significantly impact downstream results. Transparency in quality metrics are needed to improve the field of genome science and encourage data reuse.

Keywords (3-10)

Accessibility, Genomics, Life Sciences, Open Data, Papilionoidea

Introduction

The explosion of available genomes across the Tree of Life has created entirely new fields of science and is changing how we investigate long-standing questions in biology. Studies of gene family evolution and gene mutation have expanded from single genes to mapping the architecture of entire genomes. Macroevolutionary studies using genomic data are now regularly being generated at impressive scales, e.g. complete Class [1], continent [2], and spanning up to 500 million years [3]. As the scope of questions addressed with genomic data continues to expand, determining the impact of read length and genome completeness on results is vital. One metric that is often applied to assembled genomes is an N50 score, a weighted median statistic of contig continuity that describes the distribution of contig lengths. The N50 value indicates that half of the assembly is contained in contigs or scaffolds equal to or larger than the value. Assemblies with low N50s are more fragmented and the contigs or scaffolds have less overlap with one another. Completeness of a draft assembly can also be assessed using BUSCO scores [4]. This measure uses a taxonomically informed set of "core" protein-coding orthologs that are theoretically present in a given taxon to evaluate genomic completeness. BUSCO may detect both haplotypes sequenced from diploid tissue with adequate genome coverage. However, high heterozygosity can lead to more fragmented assemblies (low N50), potentially reducing the number of complete protein coding genes recovered. These scores can be influenced by biological variation through natural variation in chromosome length or lineage-wide loss of core orthologs, but also by systematic error, as in poor sequencing depth [4]. Genomes may be of low quality in terms of continuity, completeness, or a combination of these two metrics. Understanding how genomes with low quality metrics impact downstream analyses is critical.

Here, we provide draft *de-novo* genome assemblies and quality metrics for butterflies that will be useful for studying Lepidoptera evolution, gene discovery, and genomics. In order to understand how genome quality varies across taxa, we examine genome assembly quality in

this exemplar group of organisms that has more than 935 published genomes. Additionally, we explore potential uses of these data, bearing in mind their draft nature, and discuss the state of butterfly genomics in light of genome quality.

Using lepidopteran genomes, novel genes with important ecological implications arising through gene duplication can be identified, such as in plant detoxification [5]. Additionally, expansions of a particular gene copy can be indicative of functional adaptation (e.g. [6,7]). However, inaccurate assessment of gene copy number will lead to false interpretations. Denton et al. [8] document a pattern of gene misassembly and false gene duplication rates in draft genomes, with gene number either over- or under-estimated in 40% of all gene families. The mechanism of such error is closely tied to N50, such that when genes are fragmented (low N50), and multiple contigs are assembled into non-biological contigs [8]. These types of errors will present as misidentification of gene duplication and loss, as well as non-biological mutations. Gene family evolution and mutation holds immense potential in uncovering the mechanisms behind rapid functional adaptation and potential subsequent speciation [9,10], and significant progress is being made in this area with the inclusion of genomic data [3]. We recommend including sequences of known identity to identify regions of sequencing artefacts or incorrect annotation, and implementing assembly error estimation (Han et al. 2013), which may mitigate these challenges.

Phylogenetic studies stand to gain enormous taxonomic ground into the 2020s, primarily due to the explosion of low-coverage genomes that are particularly well-suited for phylogenetic studies. Taxonomic coverage in phylogenetic studies is increasing exponentially with the ability to sequence genomes from historical or museum specimens. Advances in both cost and quality of sequencing, as well as the ability to sequence DNA from degraded museum samples [15–18] allows researchers to now produce phylogenies including all extant, and even extinct species in a taxonomic group [19]. Stringency standards for including genomes in phylogenetic studies are not well established, and poor-quality genomes can produce erroneous assemblies of genes of

interest, as detailed above [8]. Further, quality scores that highlight the completeness of the genome may serve an important quality-control step for the inclusion of genomes in phylogenies, and we recommend researchers to prioritize this quality metric for phylogenetic inference. A more complete genome suggests that the sample possesses common and complete protein coding genes, and thus it is more likely to include the researcher's set of orthologs. By assessing genome completeness, future systematic error due to taxa with low matrix occupancy may be avoided [20].

Despite the challenges that low quality and low coverage genomes present, the 822 *de-novo* genomes we provide can enhance existing research programs at no-cost.

Methods

We obtained all previously published genome assemblies and genomic reads of butterflies (Lepidoptera: Papilionoidea) from the NCBI [21] and LepBase [38] databases as of July 1, 2020. In the case of NCBI genome assemblies, we searched using the taxonomy database (keywords Papilionoidea and papilionoid) for the latest assemblies, selecting the most recently submitted assembly, when multiple were available (as of July 1, 2020; see Table S1). We also searched the SRA database [21] and published literature for available paired-end, whole-body, whole shotgun genome sequences of papilionoid species [16,22–33] (search terms butterfly genome; papilionoid genome; butterfly shotgun genome; searches concluded on July 1, 2020).

We trimmed reads using TrimGalore [45] requiring a quality score of 20 and read length of 30. We assembled reads using SPAdes (SPAdes, RRID:SCR_000131) v3.13 [34] using paired reads and allowing values of K to vary based on read length. For the majority of the *de-novo* genomes, 32 CPU and 128 Gb of memory were sufficient. Forty genomes required additional memory; we ran these genomes with 24 threads with 720 Gb of memory, potentially due to deeper sequencing or greater genomic complexity.

Following assembly, we performed several post-processing steps to ensure sequence integrity. First, we identified and removed contigs comprised of less than 200 base pairs using SeqTK (Seqtk, RRID:SCR_018927) [46]. We scanned for evidence of vector contamination using VecScreen (VecScreen, RRID:SCR_016577) [47] and removed affected contigs. Then, we used the NCBI contaminant screening database to identify common contaminants, such as from fungi or bacteria, and removed those contaminant sequences.

To assess assembly quality, we first used assembly-stats [48] to quantify Scaffold N50 for each cleaned, contaminant-free, assembly. This measure estimates the contiguity of assembly contigs and describes the contig length of half of the genome; i.e., 50% of the genome includes contigs greater than or equal to this length. We also used BUSCO (BUSCO, RRID:SCR_015008) v3.02 [4] to determine the presence of a set of 1,658 core insect single-copy genes (version 9) which are highly conserved across insects and give an approximation of the completeness of the assembly. Herein, we evaluate only the BUSCO Complete score, which requires each of the 1,658 core ortholog genes in the assembly to include both start and stop codons. For the full BUSCO score report, please see Supplemental Tables S1-S3. At this point, we submitted the assemblies to the NCBI TPA database, and minor changes were requested by NCBI. We created a custom script to automate the edits requested by NCBI, filter_seqs_by_NCBI.py (Supplemental File 1). This script uses the text feedback file from NCBI and will be useful for others willing to make their assembly available for future researchers.

Results

We assembled 873 papilionoid genomes using raw reads from the NCBI SRA database and downloaded 62 pre-assembled genomes from the NCBI Assembly database [21]. These 935 butterfly samples with genomic data represent 665 unique species, because some species have multiple subspecies sequenced or have replicate genomes (Table S1). We did not attempt to combine genomic reads from multiple conspecific individuals, as this will artificially increase

heterozygosity and inevitably impact assembly quality [35]. All genomes assembled for this study (Table S1) are available for download through the TPA Database (BioProject PRJNA606954) and quality statistics calculated for each genome are listed in Table S1.

Pre-assembled genomes from NCBI and LepBase span six butterfly families and twelve subfamilies; our *de-novo* assembled genomes represent six families and twenty-four subfamilies (Figure 1). The only family for which no public genomic data available is the Hedylidae, a family with only 36 described Neotropical species [36]. Hesperiidae has the greatest number of species with available genomic data (473), over half of which are in subfamily Pyrginae (310), largely due to research by Grishin and colleagues [16,22–29,32,33] (Figure 1). The Nymphalidae, the family the most species-rich family of butterflies has 287 genomes available, and 210 of these genomes are in the genus *Junonia* (Figure 1). The Lycaenidae has comparatively few genomes available (10), given its high species richness (Figure 1).

The metrics we used revealed large variance in genome assembly quality. N50 and BUSCO scores are often similar, such that the highest quality genomes typically have both high N50 and BUSCO scores, although this was not always the case (Figure 2). These quality statistics measure two different aspects of quality and should be used in conjunction, as length distribution may not be associated with gene content [4]. Pre-assembled genomes downloaded from NCBI and LepBase on average had high quality scores (Table S2, Figure 2) (Scaffold N50 = 1,706,589; BUSCO = 81.2%). Of these, five *Heliconius* genomes (*H. hecuba flava, H. hierax, H. wallacei, H. xanthocles,* and *H. doris*) have notably lower mean quality scores (N50 = 996.6; BUSCO = 33.66%). The *Heliconius hierax* genome (GCA_900068475.1) had the lowest quality measures of the pre-assembled genomes we investigated (N50 = 916; BUSCO = 30.5). The satyrine *Aphantopus hyperantus* (GCA_902806685.1) had the highest quality scores of all genomes investigated (N50 = 15,230,192; BUSCO = 97.8%).

Quality scores varied widely among the draft *de-novo* genome assemblies (Figure 2). In fifty-one cases, we found that assemblies were comprised only of short (< 200bp) fragments

and contaminants. In these cases, we removed the assembly and report the N50 score as zero (Table S1, S3). We did not further evaluate the quality of these assemblies. N50 ranged from 249 in *Junonia evarete nigrosuffusa* (SRR10765819; Nymphalidae) to 43,550 in *Sertania guttata guttata* (Figure 2E; SRR10158585; Riodinidae). One hundred and seven *de-novo* genomes resulted in a BUSCO score of 0% (Table S1, S3), meaning that these genomes recovered none of the core insect orthologs. Seven had BUSCO scores of 90% or greater, with the greatest BUSCO score (96.4%) from *Papilio antimachus* (Figure 2D; SRR8954523 [31]). The mean quality scores of the *de-novo* genomes were low (N50 = 15,650; BUSCO = 28.25%; excluding zero values). *Proboscis propylea* (Figure 2H) had a greater than average BUSCO score, but low N50 (N50 = 605; BUSCO = 45.3%). In an effort to evaluate the variation in genome quality and identify the best exemplar genome for each major butterfly lineage, we present the highest quality genomes per subfamily (Table 1). Table 2 summarizes the fifty highest quality butterfly *de*-novo and preassembled genomes, regardless of taxonomy.

Discussion

High-quality genomes are required for studies that span the biological sciences, from gene family, mutation research to macroevolutionary phylogenetics and population dynamics. Our results show that available genomes vary widely in quality and taxonomic coverage. The significant variance in N50 and BUSCO scores highlight an important message: in the scientific literature, a “genome” can range from genomic fragments to fully annotated chromosomes. Large-scale genomic studies, especially those that sequence species in an entire clade or geographic region represent great scientific feats, but if they are based on many low-quality genomes, they may not be useful for subsequent studies. We encourage peer-reviewed journals and public databases to require authors to report genome quality via N50 and BUSCO, which can be accessioned with the assembly on NCBI as Global Statistics. Doing so provides maximum transparency, reproducibility, and a holistic view of future data reuse. In this way,

users can easily evaluate whether the quality of the genome is high enough to investigate gene family diversification (prioritize N50) or phylogenetic systematics (prioritize BUSCO).

Our analyses highlight the extensive variation in the quality of genomes. Part of this discrepancy may be alleviated with changes in language. Perhaps we should begin referring to low quality genomes, such as *Junonia evarete nigrosuffusa* (SRR10765819; N50 = 249; BUSCO = 0.1%) as 'genomic data', as opposed to the potentially misleading term, 'genome'. Next, accessioning all assemblies would save countless hours of computation time and allow for the validation of results. In addition, assemblies would also allow results (e.g., gene family evolution, sequence identification, ortholog determination) from previous studies to be validated. Accessioning should include low coverage draft genome assemblies which can also be deposited in the NCBI's Assembly database. These assemblies have notably lower N50 and BUSCO scores, when compared to the average assembly from NCBI and LepBase. Quality metrics of our *de-novo* assembled genomes were, in many cases, comparable to these five *Heliconius* genomes, suggesting that even low-quality genome assemblies can and should be accessioned. Including quality scores (as Global Statistics) for each draft assembly via the NCBI Assembly Database (in addition to taxon-specific genome databases, such as Lepbase [38]), would provide a transparent overview of available genomes for future studies.

Assembling genomes requires considerable computational resources and assessing genome quality simply from raw file size on GenBank can be misleading. Many studies in the biological and medical sciences rely on existing genomes and their annotations (e.g., [39]. If researchers independently assemble genomes, this can lead to duplicated effort and significant time investment. Further, if initial raw data quality is poor, assemblies may not be useful. In our study, we found that at least 51 of the 873 genomes that we assembled are ultimately unusable, and another 67 that we caution their reuse of (Table S1). These 118 samples produced assemblies that were either entirely comprised of contamination, contigs less than 200 bp, devoid of core insect genes, or a combination of these factors. However, it is possible that

alternate assembly methods could produce a greater quality assembly. Low N50 and low BUSCO assemblies are likely comprised of fragmented genes and, most likely, the contigs that are present are the result of very low sequence coverage. This low coverage is indicative of a high error rate and greater likelihood of incorrect sequence frame. As such, while we provide these extremely low-quality genomes, users should exercise caution in mining genes from these samples due to the high probability of error. Reporting N50 and BUSCO, as well as genome assemblies in manuscripts and databases, promotes transparency and discourages needless computation.

Contamination has been shown to be a pervasive pattern in genome and transcriptome sequencing projects, especially those that use multiplexed sequencing approaches [40–42]. In a recent study, Allio et al. [31] found that cross-contamination accounted for 0.26% of assembly contigs. While contaminants were removed from Allio et al. [31] using CroCo [43], and thus do not impact their results, it remains unknown how much these contaminant sequences will impact future studies that reuse these genomic data. The authors did not accession genome assemblies that had contaminants removed, and contaminants remain in accessioned reads. Further, it is impossible to repeat these necessary decontamination steps without detailed information regarding multiplex strategy [43]. Accessioning decontaminated assemblies to NCBI is a necessary and easy solution.

Our study reveals a significant lack of standardization and reporting across genomic studies as many do not provide genome assemblies and necessary quality metrics. Our main conclusions are that:

1. We provide draft assemblies and quality metrics for all butterfly genomes available at the time of this study (available through NCBI TPA database) (Table S1). We synthesize these data into tables of the 50 highest quality genomes, as well as exemplar genomes for each subfamily.

2. Long and contiguous reads, indicated by high N50 values, are one quality metric that should be reported in all studies, especially those of gene mutation, duplication or genomic architecture.
3. Phylogenetic studies are strengthened when genomes with a high completeness score, such as BUSCO, are used.
4. Researchers should present quality scores, and provide draft assemblies, in all genome publications and databases. Accessioning quality scores will enhance transparency and avoid unnecessary use of computational resources. Accessioning assemblies further promotes the FAIR Principles of interoperability and reuse by limiting contaminant sequences and allowing results to be confirmed.

Availability of supporting data

See Tables S1-S3 for genomic read accession numbers used in this study and associated meta-data. The 822 viable genome assemblies produced using SPAdes v3.13 are available in the NCBI TPA repository, BioProject PRJNA606954. Sequence assemblies, BUSCO files, scripts, and other supporting data are also available via the GigaScience database GigaDB [44].

Additional files

Table S1: TableS1Genome_020421 (1).xls

Table S2: TableS2_PreAssembledGenomes.csv

Table S3: TableS3Genome_042121.xlsx

File S1: filter_seqs_by_NCBI.py

Abbreviations

bp; Base pair

SRA; Sequence Read Archive

NCBI; National Center for Biotechnology Information

TPA: Third PArty Database

Competing interests

The authors declare that they have no competing interests.

Funding

This work was funded by the National Science Foundation Grants DEB #1541500 and #1557007 to AYK.

Acknowledgements

The authors acknowledge the University of Florida Research Computing (http://researchcomputing.ufl.edu) for providing computational resources and support that have contributed to the research results reported in this publication. We are grateful to two reviewers, Hans Zauner, and Xuan-Kun Li who provided helpful comments. Other members of the Kawahara Lab participated in thoughtful discussions that greatly improved the quality of this manuscript. We thank Laurel Kaminsky, Anupama Priyadarshini, Victoria Tran, Andrew Warren, and the FLMNH Digitization Team for providing butterfly images.

Authors' contributions

AYK conceived of the study. EAE performed data collection, data analysis, and produced the figures and scripts, with overall guidance from AYK. EAE, CGS, and AYK wrote the manuscript. CGS and EAE deposited the data.

References

1. Prum RO, Berv JS, Dornburg A, Field DJ, Townsend JP, Lemmon EM, et al. A

comprehensive phylogeny of birds (Aves) using targeted next-generation DNA sequencing. *Nature*. 2015;526:569–73.

2. Zhang J, Cong Q, Shen J, Opler PA, Grishin NV. Genomics of a complete butterfly continent [Internet]. Available from: http://dx.doi.org/10.1101/829887

3. Thomas GWC, Dohmen E, Hughes DST, Murali SC, Poelchau M, Glastad K, et al. Gene content evolution in the arthropods. *Genome Biol*. 2020;21:15.

4. Simão FA, Waterhouse RM, Ioannidis P, Kriventseva EV, Zdobnov EM. BUSCO: assessing genome assembly and annotation completeness with single-copy orthologs. *Bioinformatics*. 2015;31:3210–2.

5. Edger PP, Heidel-Fischer HM, Bekaert M, Rota J, Glöckner G, Platts AE, et al. The butterfly plant arms-race escalated by gene and genome duplications. *Proc Natl Acad Sci*. 2015;112:8362–6.

6. Brown CA, Murray AW, Verstrepen KJ. Rapid expansion and functional divergence of subtelomeric gene families in yeasts. *Curr Biol.* 2010;20:895–903.

7. Gouin A, Bretaudeau A, Nam K, Gimenez S, Aury J-M, Duvic B, et al. Two genomes of highly polyphagous lepidopteran pests (*Spodoptera frugiperda*, Noctuidae) with different host-plant ranges. *Sci Rep.* 2017;7:11816.

8. Denton JF, Lugo-Martinez J, Tucker AE, Schrider DR, Warren WC, Hahn MW. Extensive error in the number of genes inferred from draft genome assemblies. *PLoS Comput Biol.* 2014;10:e1003998.

9. Casacuberta E, González J. The impact of transposable elements in environmental adaptation. *Mol Ecol.* 2013;22:1503–17.

10. Bennetzen JL. Transposable element contributions to plant gene and genome evolution. *Plant Mol Biol.* 2000;42:251–69.

11. Zhang B, Horvath S. A general framework for weighted gene co-expression network analysis. *Stat Appl Genet Mol Biol*. 2005;4:e17.

12. Cong L, Ran FA, Cox D, Lin S, Barretto R, Habib N, et al. Multiplex genome engineering using CRISPR/Cas systems. *Science*. 2013;339:819–23.

13. Xu W, Fu W, Zhu P, Li Z, Wang C, Wang C, et al. Comprehensive analysis of CRISPR/Cas9-mediated mutagenesis in by genome-wide sequencing. *Int J Mol Sci.* 2019;20:4125.

14. Chakrabarti AM, Henser-Brownhill T, Monserrat J, Poetsch AR, Luscombe NM, Scaffidi P. Target-specific precision of CRISPR-mediated genome editing. *Mol Cell.* 2019;73:699–713.e6.

15. Burrell AS, Disotell TR, Bergey CM. The use of museum specimens with high-throughput DNA sequencers. *J Hum Evol.* 2015;79:35–44.

16. Zhang J, Cong Q, Shen J, Brockmann E, Grishin NV. Genomes reveal drastic and recurrent phenotypic divergence in firetip skipper butterflies (Hesperiidae: Pyrrhopyginae). *Proc Biol Sci.* 2019;286:20190609.

17. Gilbert MTP, Moore W, Melchior L, Worobey M. DNA extraction from dry museum beetles without conferring external morphological damage. *PLoS One.* 2007;2:e272.

18. St Laurent RA, Mielke CGC, Herbin D, Dexter KM, Kawahara AY. A new target capture phylogeny elucidates the systematics and evolution of wing coupling in sack- bearer moths. *Syst Entomol.* 2020;3:17.

19. Parham JF, Stuart BL, Bour R, Fritz U. Evolutionary distinctiveness of the extinct Yunnan

box turtle (*Cuora yunnanensis*) revealed by DNA from an old museum specimen. *Proc Biol Sci.* 2004;271 Suppl 6:S391–4.

20. Sanderson MJ, McMahon MM, Steel M. Phylogenomics with incomplete taxon coverage: the limits to inference. *BMC Evol Biol.* 2010;10:155.

21. Leinonen R, Sugawara H, Shumway M, International Nucleotide Sequence Database Collaboration. The sequence read archive. *Nucleic Acids Res.* 2011;39:D19–21.

22. Zhang J, Cong Q, Shen J, Brockmann E, Grishin NV. Three new subfamilies of skipper butterflies (Lepidoptera, Hesperiidae). *Zookeys*. 2019;861:91–105.

23. Zhang J, Shen J, Cong Q, Grishin NV. Genomic analysis of the tribe Emesidini (Lepidoptera: Riodinidae). *Zootaxa*. 2019;4668:475–88.

24. Li W, Cong Q, Shen J, Zhang J, Hallwachs W, Janzen DH, et al. Genomes of skipper butterflies reveal extensive convergence of wing patterns. *Proc Natl Acad Sci U S A.* 2019;116:6232–7.

25. Cong Q, Shen J, Borek D, Robbins RK, Otwinowski Z, Grishin NV. Complete genomes of Hairstreak butterflies, their speciation, and nucleo-mitochondrial incongruence. *Sci Rep.* 2016;6:24863.

26. Cong Q, Li W, Borek D, Otwinowski Z, Grishin NV. The Bear Giant-Skipper genome suggests genetic adaptations to living inside yucca roots. *Mol Genet Genomics.* 2019;294:211–26.

27. Cong Q, Shen J, Li W, Borek D, Otwinowski Z, Grishin NV. The first complete genomes of Metalmarks and the classification of butterfly families. *Genomics*. 2017;109:485–93.

28. Shen J, Cong Q, Borek D, Otwinowski Z, Grishin NV. Complete genome of *Achalarus*

lyciades, The first representative of the Eudaminae subfamily of skippers. *Current Genomics.* 2017;18:366–74.

29. Shen J, Cong Q, Kinch LN, Borek D, Otwinowski Z, Grishin NV. Complete genome of *Pieris rapae*, a resilient alien, a cabbage pest, and a source of anti-cancer proteins. *F1000Research.* 2016;5:2631.

30. VanKuren NW, Massardo D, Nallu S, Kronforst MR. Butterfly mimicry polymorphisms highlight phylogenetic limits of gene reuse in the evolution of diverse adaptations. *Molecular Biology and Evolution.* 2019;36:2842–53.

31. Allio R, Scornavacca C, Benoit N, Clamens A-L, Sperling FAH, Condamine FL. Whole genome shotgun phylogenomics resolves the pattern and timing of swallowtail butterfly evolution. *Syst Biol*. 2019;69:38–60.

32. Cong Q, Shen J, Warren AD, Borek D, Otwinowski Z, Grishin NV. Speciation in Cloudless Sulphurs Gleaned from Complete Genomes. *Genome Biol Evol.* 2016;8:915–31.

33. Cong Q, Borek D, Otwinowski Z, Grishin NV. Skipper genome sheds light on unique phenotypic traits and phylogeny. *BMC Genomics*. 2015;16:639.

34. Bankevich A, Nurk S, Antipov D, Gurevich AA, Dvorkin M, Kulikov AS, et al. SPAdes: a new genome assembly algorithm and its applications to single-cell sequencing. *J Comput Biol.* 2012;19:455–77.

35. Kajitani R, Toshimoto K, Noguchi H, Toyoda A, Ogura Y, Okuno M, et al. Efficient de novo assembly of highly heterozygous genomes from whole-genome shotgun short reads. *Genome Res*. 2014;24:1384–95.

36. Kawahara AY, Breinholt JW, Espeland M, Storer C, Plotkin D, Dexter KM, et al.

Phylogenetics of moth-like butterflies (Papilionoidea: Hedylidae) based on a new 13-locus target capture probe set. *Mol Phylogenet Evol.* 2018;127:600–5.

37. Nishikawa H, Iijima T, Kajitani R, Yamaguchi J, Ando T, Suzuki Y, et al. A genetic mechanism for female-limited Batesian mimicry in *Papilio* butterfly. *Nat Genet*. 2015;47:405–9.

38. Challi RJ, Kumar S, Dasmahapatra KK, Jiggins CD, Blaxter M. Lepbase: the Lepidopteran genome database [Internet]. Available from: http://dx.doi.org/10.1101/056994

39. Venter JC, Adams MD, Myers EW, Li PW, Mural RJ, Sutton GG, et al. The sequence of the human genome. *Science.* 2001;291:1304–51.

40. Ballenghien M, Faivre N, Galtier N. Patterns of cross-contamination in a multispecies population genomic project: detection, quantification, impact, and solutions. *BMC Biology.* 2017;15:e25.

41. Jun G, Flickinger M, Hetrick KN, Romm JM, Doheny KF, Abecasis GR, et al. Detecting and estimating contamination of human DNA samples in sequencing and array-based genotype data. *Am J Hum Genet.* 2012;91:839–48.

42. Merchant S, Wood DE, Salzberg SL. Unexpected cross-species contamination in genome sequencing projects. *PeerJ.* 2014;2:e675.

43. Simion P, Belkhir K, François C, Veyssier J, Rink JC, Manuel M, et al. A software tool “CroCo” detects pervasive cross-species contamination in next generation sequencing data. *BMC Biol.* 2018;16:e28.

44. Ellis EA; Storer CG; Kawahara AY Supporting data for "De-novo genome assemblies of butterflies" GigaScience Database. 2021. http://dx.doi.org/10.5524/100856

45. TrimGalore. https://github.com/FelixKrueger/TrimGalore).

46. SeqTK https://github.com/lh3/seqtk

47. VecScreen https://github.com/aaschaffer/generate_vecscreen_candidates

48. assembly-stats https://github.com/sanger-pathogens/assembly-stats

Table and Figure captions below.

Table 1. Highest quality genomes by butterfly subfamily, according to N50 and BUSCO scores.

Table 2. Highest 50 quality papilionoid genome assemblies, regardless of subfamily, ranked using natural log normalized N50 and BUSCO Complete scores.

Figure Captions

Figure 1. Pre-assembled and *de-novo* assembled genomes for each butterfly and subfamily shown on a topological sketch of Espeland et al. (2018). Species-richness estimates and topology are presented for comparison only.

Figure 2. Natural log normalized N50 and BUSCO scores plotted for both pre-assembled (squares) and *de-novo* (circles) genome assemblies. Colors denote Taxonomic Family designation, as in Figure 1. Letters correspond to inset images of representative species.

Additional files

Table S1. Sample ID, N50, BUSCO, and sequencing metadata for *de-novo* assembled genomes.

Table S2. Sample ID, N50, BUSCO, and sequencing metadata for pre-assembled genomes.

Table S3. Sample ID, N50, BUSCO, and sequencing metadata for *de-novo* genomes resulting in extremely poor quality assemblies.

File S1. Filter_seqs_by_NCBI.py script used to automatically update assemblies with the feedback file from NCBI during the NCBI Accession process.

Click here to access/download;Table;Table 1 best in subfamily 042121.pdf

Taxonomy	Organism	Accession ID	N50	BUSCO 3 (C%)
Hesperiidae; Coeliadinae	*Choaspes benjaminii*	SRR7174556	2,532	87.3
Hesperiidae; Eudaminae	*Phocides pigmalion*	SRR7174453	9,497	76.7
Hesperiidae; Hesperiinae	*Megathymus ursus*	GCA_003671415.1	4,153,133	98.3
Hesperiidae; Heteropterinae	*Dalla quadristriga*	SRR9330377	4,259	69.8
Hesperiidae; Pyrginae	*Cecropterus lyciades*	GCA_002930495.1	558,064	97.3
Hesperiidae; Trapezitinae	*Toxidia parvulus*	SRR9330370	932	21.7
Lycaenidae; Curetinae	*Curetis bulis*	SRR10158559	1,108	28.3
Lycaenidae; Polyommatinae	*Cyclargus thomasi*	SRR6727422	13,909	91.3
Lycaenidae; Theclinae	*Calycopis cecrops*	GCA_001625245.1	233,537	95.5
Nymphalidae; Charaxinae	*Charaxes varanes*	SRR5175869	1,531	49.5
Nymphalidae; Danainae	*Danaus plexippus*	GCA_009731565.1	9,209,872	93.9
Nymphalidae; Heliconiinae	*Heliconius erato*	LepBase_Heliconius_era	10,688,973	97.4
Nymphalidae; Limentidinae	*Limenitis arthemis*	SRR1504973	631	12.6
Nymphalidae; Morphinae	*Taenaris catops*	GCA_009936525.1	1,720,500	35.2
Nymphalidae; Nymphalinae	*Vanessa tameamea*	GCA_002938995.1	2,988,984	98.3
Nymphalidae; Satyrinae	*Aphantopus hyperantus*	GCA_902806685.1	15,230,192	97.8
Papilionidae; Baroniinae	*Baronia brevicornis*	SRR8954515	1,886	59
Papilionidae; Papilioninae	*Papilio xuthus*	GCA_000836235.1	6,198,915	97.6
Papilionidae; Parnassiinae	*Sericinus montela*	SRR8954536	3,584	59.4
Pieridae; Coliadinae	*Zerene cesonia*	GCA_012273895.1	9,214,832	95.6
Pieridae; Dismorphiinae	*Leptidea sinapis*	GCA_900199415.2	857,189	97.2
Pieridae; Pierinae	*Pieris napi*	LepBase_Pieris_napi_v1	12,597,868	94.4
Riodinidae; Nemeobiinae	*Euselasia chrysippe*	SRR10158562	1,806	30.3
Riodinidae; Riodininae	*Calephelis nemesis*	GCA_002245505.1	206,312	95.6

Rank	Accession ID	Organism	N50	BUSCO (C%)
1	GCA_902806685.1	*Aphantopus hyperantus*	15,230,192	97.8
2	LepBase_Pieris_napi_v1.1	*Pieris napi*	12,597,868	94.4
3	LepBase_Heliconius_erato_demophoon	*Heliconius erato*	10,688,973	97.4
4	GCA_009731565.1	*Danaus plexippus*	9,209,872	98
5	GCA_012273895.1	*Zerene cesonia*	9,214,832	95.6
6	GCA_000836235.1	*Papilio xuthus*	6,198,915	97.6
7	GCA_011763625.1	*Papilio bianor*	13,111,833	65
8	GCA_003671415.1	*Megathymus ursus*	4,153,133	98.3
9	GCA_003118415.2	*Papilio memnon*	4,560,862	92.9
10	GCA_000836215.1	*Papilio polytes*	3,672,263	91.8
11	GCA_002938995.1	*Vanessa tameamea*	2,988,984	98.3
12	LepBase_Junonia_coenia_JC_v1.0	*Junonia coenia*	1,571,165	98.2
13	GCA_004959915.1	*Danaus chryssipus*	1,465,393	93.9
14	GCA_001298355.1	*Papilio machaon*	1,174,287	95.5
15	GCA_008963455.1	*Hypolimnas misippus*	1,011,763	98.1
16	GCA_900199415.2	*Leptidea sinapsis*	857,189	97.2
17	GCA_010014825.1	*Danaus melanippus*	889,656	89.4
18	GCA_900239965.1	*Bicyclus anynana*	638,282	97.6
19	GCA_001856805.1	*Pieris rapae*	617,301	98
20	GCA_013186455.1	*Papilio dardanus*	596,599	94.3
21	GCA_002930495.1	*Cecropterus lyciades*	558,064	97.3
22	GCA_001278395.1	*Lerema accius*	525,349	95.1
23	GCA_001586405.1	*Phoebis sennae*	299,140	91.1
24	GCA_009936525.1	*Taenaris catops*	1,720,500	35.2
25	GCA_001625245.1	*Calycopis cecrops*	233,537	95.5
26	GCA_000931545.1	*Papilio glaucus*	230,841	95.5
27	GCA_002245505.1	*Calephelis nemesis*	206,312	95.6
28	GCA_010014985.1	*Delias pasithoe*	193,720	96.5
29	GCA_000313835.2	*Heliconius melpomene*	194,302	95.6
30	GCA_009667785.1	*Maniola jurtina*	212,945	88.3
31	GCA_002245475.1	*Calephelis virginiensis*	175,106	93.9
32	LepBase_Heliconius_burneyi_helico3	*Heliconius burneyi*	106,325	96.5
33	GCA_000716385.1	*Melitaea cinxia*	119,328	83
34	GCA_009982905.1	*Colias croceus*	95,765	92.5
35	LepBase_Heliconius_hecalesia_helico3	*Heliconius hecalesia*	68,855	96.5
36	Lepbase_Heliconius_demeter_helico3	*Heliconius demeter*	67,995	96.8
37	LepBase_Heliconius_besckei_helico3	*Heliconius besckei*	64,778	95.8
38	LepBase_Heliconius_himera_helico3	*Heliconius himera*	48,684	96.5
39	LepBase_Heliconius_sara_helico3	*Heliconius sara*	43,390	94.3
40	LepBase_Heliconius_telesiphe_helico3	*Heliconius telesiphe*	42,672	94.7
41	LepBase_Eueides_tales_helico3	*Eueides tales*	32,552	94.7
42	SRR7174358	*Megathymus ursus*	24,120	90.7
43	LepBase_Agraulis_vanillae_helico3	*Agraulis vanillae*	21,413	94.6
44	LepBase_Dryas_iulia_helico3	*Dryas iulia*	21,916	92.3
45	SRR4341246	*Delias oraia*	18,269	92.3
46	GCA_900499025.1	*Pararge aegeria*	16,525	88
47	SRR8954516	*Atrophaneura dixoni*	14,618	93.5
48	SRR6727422	*Cyclargus thomasi*	13,909	91.3
49	SRR6727440	*Eumaeus atala*	13,611	87
50	SRR10158585	*Sertania guttata*	43,550	35.1

Figure 1 Click here to access/download;Figure;figure1_072120.pdf

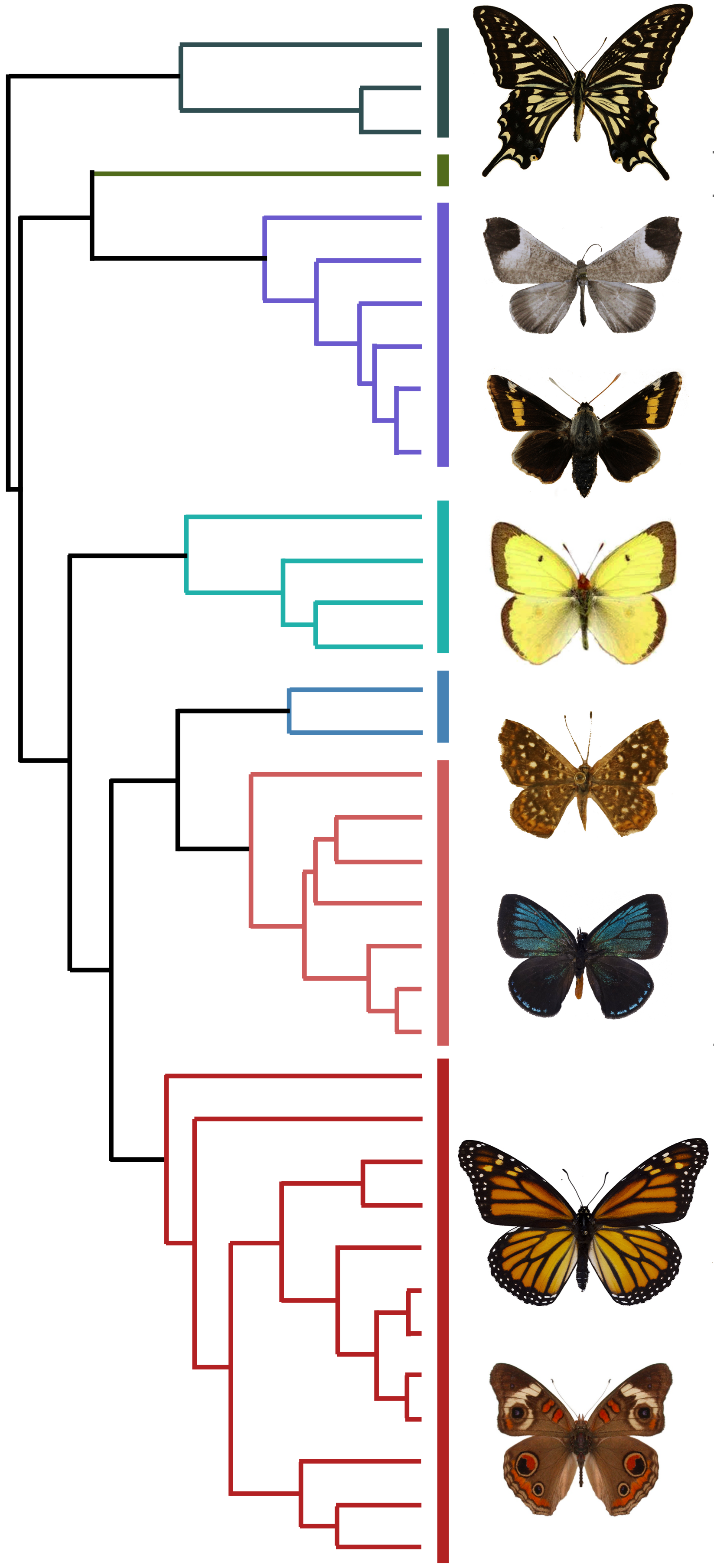

Family	Subfamily	~spp.	Genomes: Pre-Assemb.	Genomes: *De-novo*
Papilionidae (61)	Baroniinae	2	-	1
	Parnassiinae	600	13	13
	Papilioninae	1800	8	39
Hedylidae (0)	(Hedylidae)	36	-	-
Hesperiidae (472)	Coeliadinae	200	-	6
	Euschemoninae	2	-	1
	Eudaminae	600	-	92
	Pyrginae	2000	1	309
	Heteropterinae	300	-	13
	Trapezitinae	100	-	5
	Hesperiinae	3000	2	43
Pieridae (13)	Dismorphiinae	200	1	2
	Coliadinae	700	1	3
	Pseudopontiinae	5	-	-
	Pierinae	2600	3	3
Riodinidae (91)	Nemeobiinae	300	-	3
	Riodininae	1700	2	86
Lycaenidae (10)	Curetinae	60	-	1
	Aphnaeinae	280	-	-
	Portiinae	1000	-	-
	Miletinae	40	-	-
	Lycaeninae	300	-	-
	Theclinae	4200	1	2
	Polytommatinae	3300	-	6
Nymphalidae (287)	Libytheinae	35	-	-
	Danainae	1100	3	4
	Heliconiinae	1700	28	24
	Limenitidinae	2800	-	1
	Pseudergolinae	56	-	-
	Apaturinae	300	-	-
	Biblidinae	800	-	-
	Cyrestinae	200	-	-
	Nymphalinae	1300	5	212
	Calinaginae	26	-	-
	Charaxinae	1000	-	1
	Satyrinae	4700	4	4

Figure 2

Click here to access/download;Figure;Rplot_022521.pdf

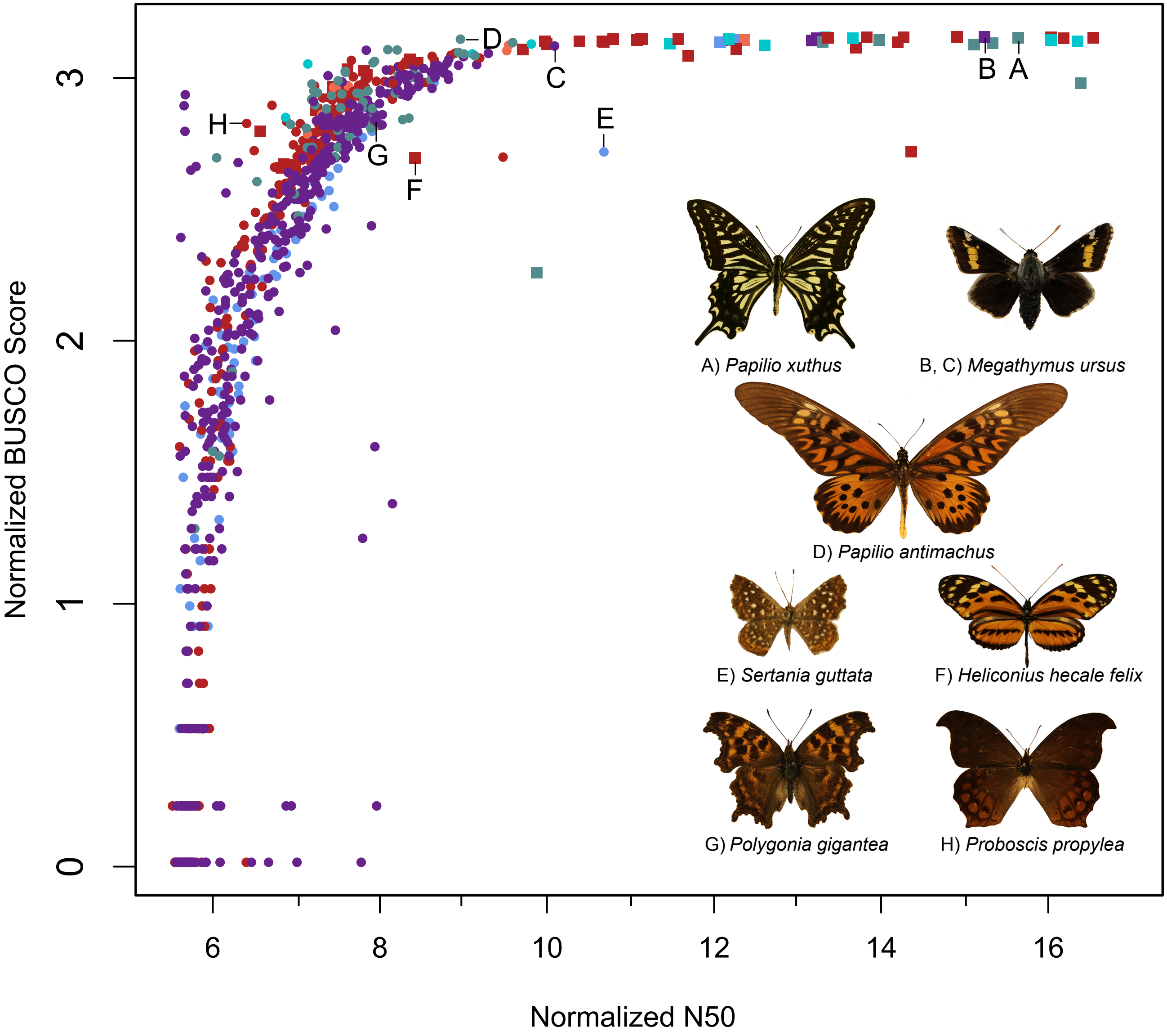

Supplementary Material File S1

Click here to access/download
Supplementary Material
filter_seqs_by_NCBI.py

Supplementary Table S1

Click here to access/download
Supplementary Material
TableS1Genome_050321.xls

Supplementary Table S2

Click here to access/download
Supplementary Material
TableS2_PreAssembledGenomes.csv

Supplementary Table S3

Click here to access/download
Supplementary Material
TableS3Genome_050321.xlsx
