# Supplementary material for: De novo genome assemblies of butterflies
Source: Gigascience. 2021 Jun 2;10(6):giab041. doi: 10.1093/gigascience/giab041 (PMC8170690; doi:10.1093/gigascience/giab041)
Supplement: giab041_Response_to_Reviewer_Comments_Revision_1 [file giab041_response_to_reviewer_comments_revision_1.pdf]

Author's Response To Reviewer Comments

Close

Dear Editor Zauner,

We are re-submitting this paper for review after incorporating feedback from the Editor and both Reviewers. We have revised the framing of the paper and adjusted the title of the paper to better fit a Resource Announcement. We thank both Reviewers for their insightful critiques and interest in our manuscript, which provides de-novo genome assemblies for all butterflies currently available, and evaluates the quality of genomes in the field. The discussion of genome quality has been reframed in order to assist in the future use of these de-novo assemblies.

Both reviewers have pointed out the potential issues with creating a new quality score metric, so we have opted to remove mention of the CQS score from the manuscript and all supplementary material. We have also revised the title of the manuscript to better reflect the product of our work. New Title: "Resource announcement: De-novo genome assemblies of butterflies"

Below, we respond to each Reviewer, in-line.

Thank you for the opportunity to revise our manuscript and respond to these important reviews. We look forward to your correspondence.

We have made the 822 de-novo genome assemblies available on GigaDB (user22@parrot.genomics.cn) as requested by reviewers for our manuscript.

We have been awaiting NCBI approval into the TPA Database, but we have decided to make the assemblies available to you and the reviewers in the interim because the process is taking a very long time (only 10% has been completed thus far). We believe that NCBI may give priority to our genome assemblies if/when our manuscript is accepted.

Emily Ellis and Akito Kawahara

Reviewer #1: Gigascience review

MS ID: GIGA-D-20-00047
Title: Genome quality variation highlights the importance of transparency for data reuse

The authors have collected a large dataset of whole genome sequencing reads from butterflies and assembled them using a unified pipeline, then analyzed the genomic quality that resulted. They then report the top 50 and bottom 50 genomes, based upon the standard N50 with BUSCO scores, and a Composite qualtity score (CQS), which is never clearly defined in the manuscript. In sum, the authors have used a lot of computational resources and generated genomes of potential use from minor datasets, to serve the research community.

Thank you for highlighting the utility of the resource that we provide. The CQS score was inadequately defined in the supplementary materials, and we apologize that it was not clear in the main body of the manuscript. However, after reading both reviews, we recognize that it may do more harm than good to the scientific community to introduce a new quality metric, especially one that combines two classically used metrics. As such, we have removed mention of the new score from the text, figures, tables, supplementary material, and our manuscript is stronger for it.

However, I am concerned about the presentation of their efforts. I find that arguing that studies generating genomic data should report quality metrics and more transparency, when the data generated was not intended for such work, problematic.

We appreciate this critique, because genomic data can have many uses, some that do not require an assembly at all. In these cases, we do not argue for their accession, but many results (synteny, gene family diversification, etc) do rely on a particular assembly. Not accessioning the particular assembly used creates an issue for results validation. By providing quality metrics and assemblies up-front (if they are used in the study), the future use of the resource can be more accurately judged. We have clarified the multitude of uses of genomic data in the text, see lines 179-184.

The work here would be more accessible to the community if the focus was presented as a mining of the existing genomic data, while acknowledging that the data was generated for a range of uses, the vast majority of which were never intended for genome assembly, but simply gene mining. Stated another way, the current line of argumentation does more harm than good. Please revise so that this can become a paper welcomed by the community.

We apologize that our original manuscript was not clear in this, our intentions were to provide accessible, mined genomic data and we have further clarified the manuscript to reflect this.

Additionally, the authors need to include currently available genomes, at least those published up through 2019. Currently they are missing many Heliconius and at least 4 Pierid genomes of high quality (e.g. Pieris rapae (Nallu et al. 2018), Pieris napi (Hill et al. 2019), Colias crocea (Woronik et al. 2019), Zerene cesonia (Rodriguez-Caro et al. 2020), Phoebis sennae (Grishin lab; Lepbase). Further, why did the authors not mine genomes off of LepBase, which is a good resource for many genome. Creating yet another database of genomes is problematic, so if you can at least include all of their genomes as well, or say that you did, that will allow you to generate a more inclusive database (Lepbase is no longer being maintained, but saying you are aware of it, and have mined everything off of it, will serve this paper well - and let people know you have included genomic resources Lepbase).

We wholeheartedly agree with this review in regards to avoiding the creation of yet another database. Thank you for raising this point. As such, we have deposited the genomes on an NCBI sub-database, the TPA (Third PArty) Database. It is our hope that this resource will be easily accessible.

The genomes of Colias crocea and Zerene cesonia were published after our manuscript was submitted to GigaScience. However, as our position is to provide a comprehensive resource on the state of butterfly genomes, we have updated the manuscript to reflect these new additions (see Supplemental Table S2). We thank the Reviewer for bringing these to our attention. We apologize for excluding these species, Lepbase was under construction leading up to the announcement of the cease of maintenance. We have also included all WGS sequences as of July 1, 2020.

I also recommend the authors cross reference with the genomes in Hill et al. 2019 (see Fig 3 and supplemental tables for data locations), which assessed genome quality across Lepidoptera and quantitatively assessed genome quality in light of contiguity and chromosomal structure, in a way much more thorough than BUSCOs.

Thank you for mentioning this important reference. We have cross-referenced the supplemental table and included the missing genomes into our study. We have also referenced this study in the Introduction, lines 52-53.

Once these issues are properly addressed, I do think this will make a positive contribution to the genomics community.

We thank the reviewer very much for their thoughtful comments and for recognizing the contribution of these genome assemblies for future use.

Major
The authors cite papers that are not peer-reviewed, and that should not be allowed, as such work may be fundamentally flawed. For example:
2. Zhang J, Cong Q, Shen J, Opler PA, Grishin NV. Genomics of a complete butterfly continent [Internet]. Available from: http://dx.doi.org/10.1101/829887 [EDITOR'S NOTE: Actually, at GigaScience we do allow , even encourage, citation of preprints. If there is a published version, please cite the journal article, but if the material is only presented as a preprint, citing the preprint is totally fine with GigaScience policy].

We do not believe this manuscript is published yet and have kept the citation of the biorxiv work.

BUSCO mention in the introduction fails to discuss the importance of this for diploid genomic content, and this is significant omission.

Genome quality can also vary significantly in its haploid state, and this also need to be taken into account, but is not mentioned in the introduction

This is an important point, thank you. We have addressed this comment in the introduction, lines 42-45.

The authors seem to have entirely missed the online database for Lepidopteran genomes, called LepBase (http://ensembl.lepbase.org/index.html).

As stated above, Lepbase was unavailable from ~December to ~February (perhaps intermittently), we apologize for its omission in this study. We have referenced this important database in-text, as well as included all genomes from the website into our study.

What is CQS? The authors state in their methods "the Composite Quality Score (CQS), which log normalizes N50 and the number of complete BUSCO genes recovered (Table 1-2, S1-2)." But this does not define how this metric is calculated …

The CQS entirely misses the duplicated content of the genome and this therefore not very informative as to genome quality.

Answered above; we have removed this metric from the manuscript.

I find this sentence confusing in the discussion "These Heliconius genomes are useful for the field, but users are not mislead into …" please restate for greater clarity.

Clarified; lines 183-187. Our point here was that although these particular Heliconius genomes are of significantly lower quality than other butterfly genomes on the Assembly database, they are still available and useful for future studies. Because quality metrics are listed in the Assembly database, they can be evaluated quickly by future users, who can then decide whether the quality is adequate in their particular use scenario.

Reviewer #2: Dear Authors

I enjoyed reading this paper because, like you, I also deeply care about assembly quality! Most people don't realise that a genome assembly is just an approximation and the quality of that approximation affects what one does and can do downstream. So thank you for addressing this issue.

Thank you very much for your kind comments, we are glad to hear that our message on genome quality came through in the manuscript. We feel that the term Genome more adequately represents data that is highly contiguous and well-resolved at the chromosomal level. Clarifying this point of language will move the field forward. We have further refined these points in the Discussion, lines 174-176.

I also deeply appreciate the sheer amount of effort and technical nous that has gone into collecting/collating/managing/running 600+ assemblies.

Thank you. We think that this resource will be useful for others in the field, so that they do not have to expend needless computational resources.

However, I have the following concerns:

1. What is the mathematical definition of CQS

I couldn't see the formula for how a CQS is calculated anywhere. Perhaps it was tucked away and I missed it? My apologies if so.

2. How is CQS better than just listing the N50 and Busco score?

One could argue that seeing the two numbers side by side is more useful as someone can choose to use a highly fragmented assembly as long as it is busco complete if all one wants to do is find orthologs for core genes (for a phylogeny, perhaps)

Thank you for raising this point, which was also raised by Reviewer 1. We have addressed this comment above, but to summarize: We now recognize that adding an additional quality metric into the field is less useful than we had previously thought. In fact, it may even complicate matters, since the metric does not incorporate new information on quality, but rather combines to classically used scores. As such, we have removed mention of this score.

3. What pre- or post- processing was done for the SPADES assemblies?

We have increased our discussion of the SPAdes assemblies and their processing in lines 101-112.

You have done a lot of work, and the protocol you describe (trim-galore + spades with varying k-mers) is technically accurate but I doubt anyone would actually do a genome assembly this way. They would do pre-filters for k-mers perhaps and assess the k-mer plots, or look for contaminants first using blobtoolkit etc

We agree with this comment and have used it to further clarify the manuscript, particularly in lines 213-215. The resource we provide is not intended to be the end-all-be-all for these species' genomes, but rather a starting point. We can imagine one practical use of these genome assemblies as a resource for researchers interested in a particular gene across butterflies, or orthology inference. In this use-case, the particular protocol we used would be sufficient, and we wholeheartedly agree that other use-cases may require a protocol that you describe.

In terms of post-processing, we have now included detailed methods on post-processing of these draft genomes. We also cite useful github pages, so that others can more easily accession their own assemblies to NCBI. During this project, we noticed that the requirements to submit assemblies to NCBI are unknown until after submission, which creates a lot of extra work for the submitter -- modifying and resubmitting assemblies with each new requirement. Our post-processing steps outlined will aid others in their own accessioning of assemblies.

--

Therefore, although I greatly appreciate the authors' efforts to make their data available and their processes transparent, as things stand right now, I feel this paper doesn't contribute much as a new method or as a new insight. Perhaps it would be better rewritten so that the focus is that of an opinion letter, or a resource announcement. Currently I feel the focus is the CQS metric and it is not as well described/justified as it could be.

Thank you for your helpful comments. We have opted to shift the focus to a resource announcement in the form of a Data Note.

Best regards
Sujai Kumar
The University of Edinburgh

Close
