# Supplementary material for: De novo genome assemblies of butterflies
Source: Gigascience. 2021 Jun 2;10(6):giab041. doi: 10.1093/gigascience/giab041 (PMC8170690; doi:10.1093/gigascience/giab041)
Supplement: giab041_Response_to_Reviewer_Comments_Revision_2 [file giab041_response_to_reviewer_comments_revision_2.pdf]

Author's Response To Reviewer Comments

Close

Dear Editor Zauner and Reviewers,

We thank you for your continued dedication and patience to our manuscript during a truly exceptional year. We have responded to Dr. Chris Wheat's comments below and provide an updated manuscript with these concerns addressed (using Track Changes). I will also provide the supplemental tables in an email because he noted he was unable to access these materials.

In addition to the changes suggested by the reviewer, we detail the use of a custom script we provide. This script will be useful for researchers accessioning assemblies on NCBI, which is something we advocate in our manuscript. We hope that by making accession easier, future researchers will make their assemblies available for data reuse and validation, in accordance with the FAIR principles.

We have also added an additional author to our study. Dr. Caroline Storer has assisted in depositing the main product of our study, the butterfly genomes, as well as participated in the editing of the manuscript. We felt that this substantial contribution constituted authorship and have updated the author information as such.

Below, we respond to the specific points of the review (our responses are marked with lines beginning with +) and we look forward to hearing from you.

Best wishes,
Emily Ellis

Re-review

Lep genome review

Great job on the revisions, its certainly much better now and close to being ready. Just some questions and minor editorial issues below.

+Thank you very much for your assistance in clarifying our message. We appreciate the attention to detail and the shared commitment to genome quality and data reuse.

Pre-assembled genomes downloaded from NCBI and Lepbase generally had high quality scores - the metrics after this, are these average values, or medians, or ….? Be more clear please.

+Fixed, thank you.

I could never open Table S2, but many of the issues below likely apply. I also hope the authors included not just the data generated per species, but the assembler sortware, etc.

+We apologize that the reviewer could not access our supplemental table 2! We are not certain how this happened, but we have updated the Excel version type in this resubmission, as well as include supplemental table 2 in an email to the Editor. Yes, in Supplemental Tables 1, 2, and 3 we have include full and detailed set of information regarding each specimen and assembly (if available).

Also, what N50 are they reporting? Scaffold or contig or pruned to a standardized min cutoff?

+Thank you for raising this important clarification. We are reporting Scaffold N50 and have now referenced it as such in the Manuscript, Main Tables and Figures, as well as Supplemental Materials.

"We found that five pre-assembled Heliconius genomes had notably lower N50 and BUSCO scores, when compared to the average pre-assembled genome downloaded from NCBI and Lepbase" - pre-assembles vs. pre-assembles … where did the first set come from? Add more clarity here, this is confusing as it stands.

+Yes, this is a confusingly worded sentence, particularly from the overuse of the word 'pre-assembled'. We have edited it in the text to better illustrate our point. To elaborate here: The average genome downloaded from LepBase and NCBI have high N50 and BUSCO scores, but the five Heliconius genomes that we are referencing here have notably lower scores.

Pre-assembled genomes: you did not polish your genomes before running BUSCO, so your BUSCO scores are going to be uniformly lower due to this. State as much to show you know and care.

+We think the reviewer is referencing the fact that we did not polish our de-novo genomes prior to running BUSCO and N50. That is correct, and a point that the second reviewer raised in the previous round of review. We have further clarified this in the Results and Discussion.

"in our study, we found that at least fifty-one of the 873 genomes that we assembled are ultimately unusable, and another fifty-seven that we caution the reuse of (Table 1)", but Table1 is of the best genomes. So something is wrong here and I couldn'd find a list of the warning genomes … which is needed.

+We apologize, Table S1 is the table that should have been referenced in this sentence, not Table 1. We have now made that change, thank you for bringing this to our attention. Additionally, we have opted to include a third supplemental table of warning genomes (those that have N50 and/or BUSCO Complete score of 0%). We thank the reviewer for this idea, as it is an important contribution of our paper to caution the re-use of these particular samples.

You should also end the paragraph references above with a discussion about the dangers of using highly fragmented, low BUSCO data. There are likely to be mostly fragmented genes, with > 1 copy since scaffolds are not long enough to resolve the diploid copies, etc. Also data difficult to clean and each assembled contig likely has very low sequence coverage, and thus a higher error rater compared to high quality genome data which is effectively error corrected when polished due to high depth of read coverage. This then places your findings with the relevant context and warns the readers more efficevly. Also, without polishing, frame is very likely off in many genes, so mining these likely mines out junk.

+Thank you for mentioning this, we are similarly concerned with the hazards of using low quality genomes. Our first submission had a more substantial discussion of these issues. We have added some of this discussion back into the manuscript and feel it is a substantial improvement. Along these same lines, by including the Supplemental Table 3, also suggested by this reviewer, we are further highlighting how prevalent low quality genome assemblies are. Future users of these data need to be aware this!

Figure 2 would be more informative to have a bit more jitter and color dots by family

+We agree that the amount of overlap in the scatterplot makes the plot difficult to uncover particular data points. Thank you for the idea to color the dots by family. This is an informative change, and also potentially alleviates the over-plotting issue by introducing some variation in dot color.

Minor issues:

Table 1 … add thousands commas please.

+Added, thank you.

Supplemental table S1.
What are Avergae? MBases, Mbytes?
Put N50 in its own column, adding thousands commas
Put n scaffolds in its own column, adding thousands commas
Put family in its own column
Put subfamily in its own column s.

+Fixed, thank you for these helpful clarifications, and we have further updated Table S2 with these same changes.

Close
