# Supplementary material for: De novo genome assemblies of butterflies
Source: Gigascience. 2021 Jun 2;10(6):giab041. doi: 10.1093/gigascience/giab041 (PMC8170690; doi:10.1093/gigascience/giab041)
Supplement: giab041_Response_to_Reviewer_Comments_Revision_3 [file giab041_response_to_reviewer_comments_revision_3.pdf]

Author's Response To Reviewer Comments

Close

Dear Editor Zauner,

All assemblies associated with this Data Note are now uploaded and accepted into the NCBI TPA Database under the BioProject PRJNA606954. These assemblies do not have an associated accession number, instead the assembly is linked to the proper BioSample ID. So that you and the reviewers can see how this works, I released 35 assemblies and ready to release the remaining 787 assemblies.

Thank you very much for all of your help with this manuscript.

All my best,
Emily
I am ready to release all assemblies,

Close
