# Supplementary material for: De novo genome assemblies of butterflies
Source: Gigascience. 2021 Jun 2;10(6):giab041. doi: 10.1093/gigascience/giab041 (PMC8170690; doi:10.1093/gigascience/giab041)
Supplement: giab041_Reviewer_1_Report_Original_Submission — Chris Wheat, PhD -- 3/31/2020 Reviewed [file giab041_reviewer_1_report_original_submission.pdf]

**Reviewer Report**

**Title: De-novo genome assemblies of butterflies**

**Version: Original Submission** **Date:** 3/31/2020

**Reviewer name: Chris Wheat, PhD**

**Reviewer Comments to Author:**

Gigascience review
MS ID: GIGA-D-20-00047
Title: Genome quality variation highlights the importance of transparency for data reuse
The authors have collected a large dataset of whole genome sequencing reads from butterflies and assembled them using a unified pipeline, then analyzed the genomic quality that resulted. They then report the top 50 and bottom 50 genomes, based upon the standard N50 with BUSCO scores, and a Composite qualtity score (CQS), which is never clearly defined in the manuscript. In sum, the authors have used a lot of computational resources and generated genomes of potential use from minor datasets, to serve the research community.
However, I am concerned about the presentation of their efforts. I find that arguing that studies generating genomic data should report quality metrics and more transparency, when the data generated was not intended for such work, problematic.
The work here would be more accessible to the community if the focus was presented as a mining of the existing genomic data, while acknowledging that the data was generated for a range of uses, the vast majority of which were never intended for genome assembly, but simply gene mining. Stated another way, the current line of argumentation does more harm than good. Please revise so that this can become a paper welcomed by the community.
Additionally, the authors need to include currently available genomes, at least those published up through 2019. Currently they are missing many Heliconius and at least 4 Pierid genomes of high quality (e.g. Pieris rapae (Nallu et al. 2018), Pieris napi (Hill et al. 2019), Colias crocea (Woronik et al. 2019), Zerene cesonia (Rodriguez-Caro et al. 2020), Phoebis sennae (Grishin lab; Lepbase). Further, why did the authors not mine genomes off of LepBase, which is a good resource for many genome. Creating yet another database of genomes is problematic, so if you can at least include all of their genomes as well, or say that you did, that will allow you to generate a more inclusive database (Lepbase is no longer being maintained, but saying you are aware of it, and have mined everything off of it, will serve this paper well - and let people know you have included genomic resources Lepbase).

I also recommend the authors cross reference with the genomes in Hill et al. 2019 (see Fig 3 and supplemental tables for data locations), which assessed genome quality across Lepidoptera and quantitatively assessed genome quality in light of contiguity and chromosomal structure, in a way much more thorough than BUSCOs.
Once these issues are properly addressed, I do think this will make a positive contribution to the genomics community.

Major
The authors cite papers that are not peer-reviewed, and that should not be allowed, as such work may be fundamentally flawed. For example:
2. Zhang J, Cong Q, Shen J, Opler PA, Grishin NV. Genomics of a complete butterfly continent [Internet]. Available from: http://dx.doi.org/10.1101/829887
BUSCO mention in the introduction fails to discuss the importance of this for diploid genomic content, and this is significant omission.
Genome quality can also vary significantly in its haploid state, and this also need to be taken into account, but is not mentioned in the introduction
The authors seem to have entirely missed the online database for Lepidopteran genomes, called LepBase (http://ensembl.lepbase.org/index.html).
What is CQS? The authors state in their methods "the Composite Quality Score (CQS), which log normalizes N50 and the number of complete BUSCO genes recovered (Table 1-2, S1-2)." But this does not define how this metric is calculated …
The CQS entirely misses the duplicated content of the genome and this therefore not very informative as to genome quality.
I find this sentence confusing in the discussion "These Heliconius genomes are useful for the field, but users are not mislead into …" please restate for greater clarity.

**Level of Interest**

Please indicate how interesting you found the manuscript: Choose an item.

**Quality of Written English**

Please indicate the quality of language in the manuscript: Choose an item.

**Declaration of Competing Interests**

Please complete a declaration of competing interests, considering the following questions:

- Have you in the past five years received reimbursements, fees, funding, or salary from an organisation that may in any way gain or lose financially from the publication of this manuscript, either now or in the future?
- Do you hold any stocks or shares in an organisation that may in any way gain or lose financially from the publication of this manuscript, either now or in the future?
- Do you hold or are you currently applying for any patents relating to the content of the manuscript?
- Have you received reimbursements, fees, funding, or salary from an organization that holds or has applied for patents relating to the content of the manuscript?
- Do you have any other financial competing interests?

- Do you have any non-financial competing interests in relation to this paper?

If you can answer no to all of the above, write 'I declare that I have no competing interests' below. If your reply is yes to any, please give details below.

none, blind review

I agree to the open peer review policy of the journal. I understand that my name will be included on my report to the authors and, if the manuscript is accepted for publication, my named report including any attachments I upload will be posted on the website along with the authors' responses. I agree for my report to be made available under an Open Access Creative Commons CC-BY license (http://creativecommons.org/licenses/by/4.0/). I understand that any comments which I do not wish to be included in my named report can be included as confidential comments to the editors, which will not be published.

Choose an item.

To further support our reviewers, we have joined with Publons, where you can gain additional credit to further highlight your hard work (see: https://publons.com/journal/530/gigascience). On publication of this paper, your review will be automatically added to Publons, you can then choose whether or not to claim your Publons credit. I understand this statement.

Yes Choose an item.
