# Supplementary material for: De novo genome assemblies of butterflies
Source: Gigascience. 2021 Jun 2;10(6):giab041. doi: 10.1093/gigascience/giab041 (PMC8170690; doi:10.1093/gigascience/giab041)
Supplement: giab041_Reviewer_1_Report_Revision_1 — Chris Wheat, PhD -- 8/5/2020 Reviewed [file giab041_reviewer_1_report_revision_1.pdf]

Reviewer Report

Title: De-novo genome assemblies of butterflies

Version: Revision 1 **Date:** 8/5/2020

Reviewer name: Chris Wheat, PhD

Reviewer Comments to Author:

Lep genome review
Great job on the revisions, its certainly much better now and close to being ready. Just some questions and minor editorial issues below.
Pre-assembled genomes downloaded from NCBI and Lepbase generally had high quality scores - the metrics after this, are these average values, or medians, or ….? Be more clear please.
I could never open Table S2, but many of the issues below likely apply. I also hope the authors included not just the data generated per species, but the assembler sortware, etc.
Also, what N50 are they reporting? Scaffold or contig or pruned to a standardized min cutoff?
"We found that five pre-assembled Heliconius genomes had notably lower N50 and BUSCO scores, when compared to the average pre-assembled genome downloaded from NCBI and Lepbase" - pre-assembles vs. pre-assembles … where did the first set come from? Add more clarity here, this is confusing as it stands.
Pre-assembled genomes: you did not polish your genomes before running BUSCO, so your BUSCO scores are going to be uniformly lower due to this. State as much to show you know and care.
"in our study, we found that at least fifty-one of the 873 genomes that we assembled are ultimately unusable, and another fifty-seven that we caution the reuse of (Table 1)", but Table1 is of the best genomes. So something is wrong here and I couldn'd find a list of the warning genomes … which is needed.
You should also end the paragraph references above with a discussion about the dangers of using highly fragmented, low BUSCO data. There are likely to be mostly fragmented genes, with > 1 copy since scaffolds are not long enough to resolve the diploid copies, etc. Also data difficult to clean and each assembled contig likely has very low sequence coverage, and thus a higher error rater compared to high quality genome data which is effectively error corrected when polished due to high depth of read coverage. This then places your findings with the relevant context and warns the readers more efficevly. Also, without polishing, frame is very likely off in many genes, so mining these likely mines out junk.
Figure 2 would be more informative to have a bit more jitter and color dots by family
Minor issues:
Table 1 … add thousands commas please.
Supplemental table S1.
What are Avergae? MBases, Mbytes?
Put N50 in its own column, adding thousands commas
Put n scaffolds in its own column, adding thousands commas
Put family in its own column

Put subfamily in its own column

Level of Interest

Please indicate how interesting you found the manuscript: Choose an item.

Quality of Written English

Please indicate the quality of language in the manuscript: Choose an item.

Declaration of Competing Interests

Please complete a declaration of competing interests, considering the following questions:

- Have you in the past five years received reimbursements, fees, funding, or salary from an organisation that may in any way gain or lose financially from the publication of this manuscript, either now or in the future?
- Do you hold any stocks or shares in an organisation that may in any way gain or lose financially from the publication of this manuscript, either now or in the future?
- Do you hold or are you currently applying for any patents relating to the content of the manuscript?
- Have you received reimbursements, fees, funding, or salary from an organization that holds or has applied for patents relating to the content of the manuscript?
- Do you have any other financial competing interests?
- Do you have any non-financial competing interests in relation to this paper?

If you can answer no to all of the above, write 'I declare that I have no competing interests' below. If your reply is yes to any, please give details below.

No competing interests. Dr. Kawahara has supplied a couple of bugs to me for work, but that is stand museum practice and not a competing interest.

I agree to the open peer review policy of the journal. I understand that my name will be included on my report to the authors and, if the manuscript is accepted for publication, my named report including any attachments I upload will be posted on the website along with the authors' responses. I agree for my report to be made available under an Open Access Creative Commons CC-BY license (http://creativecommons.org/licenses/by/4.0/). I understand that any comments which I do not wish to be included in my named report can be included as confidential comments to the editors, which will not be published.

Choose an item.

To further support our reviewers, we have joined with Publons, where you can gain additional credit to further highlight your hard work (see: https://publons.com/journal/530/gigascience). On publication of this paper, your review will be automatically added to Publons, you can then choose whether or not to claim your Publons credit. I understand this statement.

Yes Choose an item.
