# Supplementary material for: De novo genome assemblies of butterflies
Source: Gigascience. 2021 Jun 2;10(6):giab041. doi: 10.1093/gigascience/giab041 (PMC8170690; doi:10.1093/gigascience/giab041)
Supplement: giab041_Reviewer_2_Report_Original_Submission — Sujai Kumar -- 4/20/2020 Reviewed [file giab041_reviewer_2_report_original_submission.pdf]

Reviewer Report

Title: De-novo genome assemblies of butterflies

Version: Original Submission **Date:** 4/20/2020

Reviewer name: Sujai Kumar

Reviewer Comments to Author:

Dear Authors
I enjoyed reading this paper because, like you, I also deeply care about assembly quality! Most people don't realise that a genome assembly is just an approximation and the quality of that approximation affects what one does and can do downstream. So thank you for addressing this issue.
I also deeply appreciate the sheer amount of effort and technical nous that has gone into collecting/collating/managing/running 600+ assemblies.
However, I have the following concerns:
1. What is the mathematical definition of CQS
I couldn't see the formula for how a CQS is calculated anywhere. Perhaps it was tucked away and I missed it? My apologies if so.
2. How is CQS better than just listing the N50 and Busco score?
One could argue that seeing the two numbers side by side is more useful as someone can choose to use a highly fragmented assembly as long as it is busco complete if all one wants to do is find orthologs for core genes (for a phylogeny, perhaps)
3. What pre- or post- processing was done for the SPADES assemblies?
You have done a lot of work, and the protocol you describe (trim-galore + spades with varying k-mers) is technically accurate but I doubt anyone would actually do a genome assembly this way. They would do pre-filters for k-mers perhaps and assess the k-mer plots, or look for contaminants first using blobtoolkit etc
--
Therefore, although I greatly appreciate the authors' efforts to make their data available and their processes transparent, as things stand right now, I feel this paper doesn't contribute much as a new method or as a new insight. Perhaps it would be better rewritten so that the focus is that of an opinion letter, or a resource announcement. Currently I feel the focus is the CQS metric and it is not as well described/justified as it could be.
Best regards
Sujai Kumar
The University of Edinburgh

Level of Interest

Please indicate how interesting you found the manuscript: Choose an item.

Quality of Written English

Please indicate the quality of language in the manuscript: Choose an item.

Declaration of Competing Interests

Please complete a declaration of competing interests, considering the following questions:

- Have you in the past five years received reimbursements, fees, funding, or salary from an organisation that may in any way gain or lose financially from the publication of this manuscript, either now or in the future?
- Do you hold any stocks or shares in an organisation that may in any way gain or lose financially from the publication of this manuscript, either now or in the future?
- Do you hold or are you currently applying for any patents relating to the content of the manuscript?
- Have you received reimbursements, fees, funding, or salary from an organization that holds or has applied for patents relating to the content of the manuscript?
- Do you have any other financial competing interests?
- Do you have any non-financial competing interests in relation to this paper?

If you can answer no to all of the above, write 'I declare that I have no competing interests' below. If your reply is yes to any, please give details below.

I declare that I have no competing interests

I agree to the open peer review policy of the journal. I understand that my name will be included on my report to the authors and, if the manuscript is accepted for publication, my named report including any attachments I upload will be posted on the website along with the authors' responses. I agree for my report to be made available under an Open Access Creative Commons CC-BY license (http://creativecommons.org/licenses/by/4.0/). I understand that any comments which I do not wish to be included in my named report can be included as confidential comments to the editors, which will not be published.

Choose an item.

To further support our reviewers, we have joined with Publons, where you can gain additional credit to further highlight your hard work (see: https://publons.com/journal/530/gigascience). On publication of this paper, your review will be automatically added to Publons, you can then choose whether or not to claim your Publons credit. I understand this statement.

Yes Choose an item.
